# ﻿Unravelling *Amegilla* (*Glossamegilla*) diversity across the Wallace Line: new species, wing morphometrics, and biogeographic boundaries (Hymenoptera, Apidae)

**DOI:** 10.3897/zookeys.1256.162903

**Published:** 2025-10-16

**Authors:** Frédéric Carion, Maxence Gérard, Guillaume Ghisbain, Thomas J. Wood

**Affiliations:** 1 Laboratory of Zoology, Research Institute for Biosciences, University of Mons, Place du Parc 20, B – 7000 Mons, Belgium University of Mons Mons Belgium; 2 Naturalis Biodiversity Center, Darwinweg 2, 2333 CR, Leiden, Netherlands Naturalis Biodiversity Center Leiden Netherlands

**Keywords:** Anthophila, bees, geometric morphometrics, island biogeography, new species

## Abstract

The Indo-Australian Archipelago is a large and biologically complex region that straddles the famous transitionary border between the Indo-Malayan and Australasian biogeographic realms called the Wallace Line. This archipelago contains much of the diversity of Amegilla (Glossamegilla) Brooks, 1988 that was previously revised during the 20^th^ century by the Dutch entomologist Maurits Lieftinck, but no modern works on the subject have been made. We present a new revision of the subgenus Glossamegilla in the Indo-Australian Archipelago using wing morphometrics to validate existing species concepts, as well as more complete type revision and examination of undetermined material. These efforts result in the new synonymy of *A. bouwmani* (Lieftinck, 1944), **syn. nov.** with *A. amymone* (Bingham, 1896) and unveiled five novelties, namely *A. floresiana* Carion & Wood, **sp. nov.** (Indonesia: Flores), *A. suzanneae* Carion & Wood, **sp. nov.** (Malaysia: Sabah), *A. celineae* Carion, **sp. nov.** (Indonesia: East Kalimantan), and the unknown males of *A. gigas* (Friese, 1922) and *A. vigilans* (Smith, 1860). In total, 20 species are currently known in the Indo-Australian Archipelago. Wing morphometrics were found to strongly support most of the existing species concepts. No individual *Glossamegilla* species were found to occur on both sides of the Wallace Line, and the subgenus as a whole is not known to the east of the Lydddeker Line. Overall, wing morphometrics can help to determine *Glossamegilla* species in the Indo-Australian Archipelago but need to be coupled with other characters such as additional morphological features or biogeography. Future research involving new collections of specimens are needed to thoroughly understand the evolutionary history and biogeography of the subgenus in the Indo-Australian Archipelago.

## ﻿Introduction

The wild bee subfamily Anthophorinae is a medium-sized subfamily of the family Apidae containing seven genera, with more than 750 recorded species worldwide. This subfamily was previously treated as Anthophorini, a tribe of the subfamily Apinae (Dubitzky 2007; Michener 2007; Bossert et al. 2019; Ascher and Pickering 2020). Within Anthophorinae, the two largest genera are *Anthophora* Latreille, 1803, with more than 420 described species, and *Amegilla* Friese, 1897, with more than 220 described species, *Amegilla* being the most closely related genus to *Anthophora* (Dubitzky 2007; Michener 2007; Ascher and Pickering 2020; Orr et al. 2022). Numerous *Amegilla* were first described as *Anthophora* due to the close resemblance between the two genera and these similarities were only finally resolved during the 20^th^ century (Brooks 1988); some examples of these changing taxonomic concepts are found in the works of Lieftinck (1944, 1956).

Brooks (1988) was the first to attempt ordering both *Anthophora* and *Amegilla* into a system of subgenera, leading to the description of multiple new subgenera, especially for *Amegilla*. These subgenera are still currently in use and are solely based on both morphological characteristics and geographic distributions. This system is challenging to use in practical terms, in large part because it is based primarily on male characters, with female specimens of multiple species remaining difficult to distinguish (Brooks 1988; Michener 2007). Overall, in the system of Brooks (1988), it is sometimes easier to directly place females to species than subgenera. Within *Amegilla*, Brooks (1988) recognised 11 subgenera and one group of uncertain species (Dubitzky 2007). One of the subgenera established by Brooks is *Glossamegilla* Brooks, 1988, which the most important characteristic is the presence of a long galea (Brooks 1988). *Glossamegilla* includes approximately 30 species worldwide, all with an Indomalayan, East Palaearctic, or Australasian distribution. Most of the diversity is found in the Indomalayan region but the subgenus is more broadly distributed from India and China to south-eastern Asia (Brooks 1988; Ascher and Pickering 2020).

The present article is geographically centred on Indonesia but also includes the Malaysian part of Borneo, Brunei, and the country of Timor-Leste (thus, the whole island of Timor is considered). This region is also known as the Indo-Australian Archipelago, which consists of approximately 20,000 islands, covering a total of more than 6 million square kilometres (Lohman et al. 2011; Tsang et al. 2019). This archipelago is biologically complex and interesting due to the transition between the Indo-Malayan and the Australasian biogeographic realms as well as the presence of four major tectonic plates converging in the region, namely the Eurasian, Indo-Australian, Philippine Sea and Pacific plates (Bird 2003; Procheş and Ramdhani 2012; Holt et al. 2013; Rueda et al. 2013; Ali et al. 2020; Ali and Heaney 2021). Many biological “lines of separation” have been drawn in this region, mainly based on works concerning vertebrates, attempting to separate the Indo-Malayan and Australasian biogeographic realms, with one of the most well-known called the Wallace Line. The line, originally drawn between the islands of Bali and Lombok as well as between the islands of Borneo and Sulawesi is also one of the most western lines to separate these two realms. However, the precise delineation of this boundary has evolved substantially since the first observations by Salomon Müller (1846), the introduction of the concept of a division between the Indo-Malayan and Australasian faunas by Wallace (1863), and its subsequent reinterpretation by Huxley (1868) – a revision, never accepted by Wallace himself, which used for the first time the term “Wallace’s Line” (Brown et al. 2013; Ali et al. 2020; Ali and Heaney 2021). The current literature does not have a consensus on where the ultimate border between these realms should be and, in fact, the placement of the border seems to depend on the studied group and the authors (Ali and Heaney 2021). The lack of consensus for the placement of an ultimate border led to the hypothesis of a transition area, such as “Wallacea”, rather than a strict border, although this is also debated (Ali and Heaney 2021).

Concerning the subgenus Glossamegilla of the Indo-Australian Archipelago, only two revisionary works are known, both by the Dutch entomologist Maurits Lieftinck (Lieftinck 1944; 1956). The former work (in 1944) was a first attempt to provide a revision of the anthophorine bees of the Malaysian and Indonesian region; all the *Glossamegilla* considered in this work were then treated as *Anthophora*, and only nine species of what came to be considered *Glossamegilla* were included, although seven of these taxa were newly reported for the region. The latter work (in 1956) reassigned all the species previously treated as *Anthophora* to the genus *Amegilla* and established new synonymies inside the genus. It included 17 species, of which three are not found in the Indo-Australian Archipelago, while *A. vigilans* (Smith, 1860) (described from Sulawesi) and *A. gigas* (Friese, 1922) (described from Sumatra and Wetar) were not included without any explanation.

Morphometric analysis is characterised by the use of statistical methods to study a form, measuring variations in the shape and size of biological structures, as well as their covariations with other variables (Bookstein 1991; Claude 2008; Adams et al. 2013). More specifically, geometric morphometrics analyses the shape of anatomical structures by using Cartesian coordinates of anatomical landmarks after mathematically removing the effects of non-shape variations (Bookstein 1991; Claude 2008; Mitteroecker and Gunz 2009; Adams and Otárola‐Castillo 2013). This approach was already tested as a tool to discriminate taxa among various taxonomic groups in both Vertebrates and Invertebrates, including bees (Cardini et al. 2009; Ahmad et al. 2022; Casaubon and Riehl 2024; Özkan et al. 2024; Shukri et al. 2024), for which the landmarks are plotted on vein intersections (Francoy et al. 2008; Dehon et al. 2019; Martinet et al. 2019; Gérard et al. 2020; Ghisbain et al. 2021; Soipijit and Sopaladawan 2024). Wing morphometrics offers a valuable method to separate taxa, as the approach is less affected by specimen degradation over time compared to ethological, molecular, or chemical methods, which may require costly reagents, specialised laboratory equipment or rely on traits that can deteriorate or disappear in pinned specimens. In contrast, wings are still well-conserved if the collections are maintained in good condition and are cheaper to study (Francoy et al. 2008; Gérard et al. 2020; Soipijit and Sopaladawan 2024). Moreover, wing morphometrics has the advantage of being a completely non-destructive method (Gérard et al. 2020), making it a highly valuable approach for studying entomological collections.

Our current work aims to introduce the use of wing morphometrics in a revision of the subgenus Glossamegilla in the Indo-Australian Archipelago for the first time as well using this approach as a complementary argument within a taxonomic framework. This revision more broadly aims to consider the two species overlooked by Lieftinck (namely *A. gigas* and *A. vigilans*), reconsider morphological characters, and provide a revised key with wider consideration of the variation displayed by members of this group. We also take the opportunity to provide distribution maps in order to characterise the distribution of the subgenus and to observe the degree to which these distributions conform to the Wallace Line.

### ﻿Abbreviations

**MSNG**Museo Civico di Storia Naturale “Giacomo Doria” Genoa, Italy

**NHMUK**Natural History Museum, London, United Kingdom

**OÖLM** Oberösterreiches Landesmuseum, Linz, Austria

**OUMNH**Oxford University Museum of Natural History, Oxford, United Kingdom

**RMNH**Naturalis Biodiversity Center, Leiden, the Netherlands

**ZMHB** Museum für Naturkunde, Berlin, Germany

## ﻿Materials and methods

### ﻿Morphometric analyses

Six species of *Glossamegilla*, all hosted in the RMNH collection, were studied using geometric morphometrics. These species were chosen because their abundance in the collection was sufficient for ensuring robust statistical analyses (Cardini et al. 2015). The number of specimens studied per sex and per species varies from 13 to 20 (Table 1). The other species considered in this revision were insufficiently abundant to obtain a minimum of 10 male and 10 female specimens, and were therefore excluded from the geometric morphometrics analyses. All the specimens come from the Indo-Malayan region, mainly from Indonesia (all the data underlying the analyses are available on Zenodo following Carion et al. 2025).

**Table 1.** Number of specimens per species and per sex used for the morphometric analyses.

Species	Females	Males
*Amegilla cinnyris* (Lieftinck, 1944)	14	20
*Amegilla cyrtandrae* (Lieftinck, 1944)	20	20
*Amegilla feronia* (Lieftinck, 1944)	13	17
*Amegilla insularis* (Smith, 1857)	20	19
*Amegilla pendleburyi* (Cockerell, 1929)	20	20
*Amegilla sumatrana* Lieftinck, 1956	16	20

The right forewing of the specimens was photographed with a standardised millimetre scale placed under the wing. Specimens with wings in poor condition (i.e. broken, missing, or folded) were not used. When the right forewing could not be used, the left forewing was photographed and a mirror symmetry was applied with GIMP 2.10.36 to mimic the shape of a right forewing (~3.6% of wings used were the left forewing). All the pictures of the wings were taken with a Dino-Lite Edge numeric microscope coupled with the software DinoCapture 3.0 v. 1.1.0.0.

**Figure 1.**
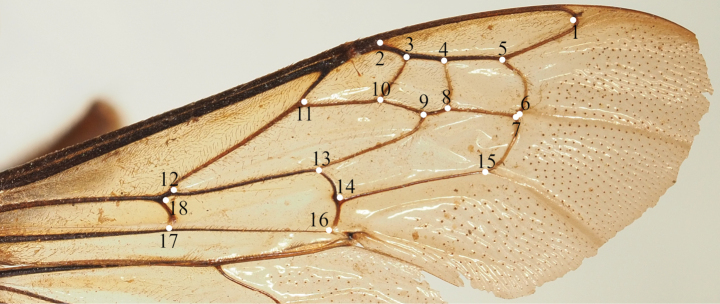
Right forewing of *Amegilla vigilans* (Smith, 1860) with the eighteen landmarks digitised in order to study the wing morphometrics.

The “Tps” software^©^ produces a .tps file with the images. First, we created and merged the tps files using tpsUtil32 v. 1.83. We then digitalised eighteen landmarks following a specific pattern (Fig. 1) and set the scale for each wing using tpsDig232 v. 2.31. At this stage, each wing has its own set of Cartesian coordinates, defined as its landmark configuration.

The analyses were conducted using Rstudio v. 2024.09.1 and R v. 4.4.2 as well as the packages geomorph, RRPP, ggplot2, MASS, and car. First, a Procrustes superimposition of the data was performed. The Procrustes approach involved standardising size, preserving it as a distinct variable, as well as minimising positional discrepancies. In practice, the landmark configuration of each wing is first scaled to unit centroid size and then translated to a common centroid. The landmark configurations are finally optimally rotated around their common centroid based on the sum of squared distances between related landmarks across specimens. Wing size was estimated using centroid size, which is defined as the square root of the sum of squared distances between each landmark and the centroid of the landmark configuration (Gérard et al. 2018). Wing sizes were then compared using a one-way analysis of variance (ANOVA), followed by a Tukey’s Honest Significant Difference (post-hoc) test to identify pairwise differences between species. To assess wing shape variation a principal component analysis (PCA) was conducted, allowing the visualisation of species clustering within the subgenus. Finally, a linear discriminant analysis (LDA) was performed to assess the discriminating power of the wing among the studied species. This was followed by a Procrustes ANOVA to determine if significant differences in wing morphometry existed among species. Pairwise comparisons were then conducted to identify which species pairs were significantly different from each other.

To evaluate the classification accuracy of the linear discriminant analysis (LDA), we performed a leave-one-out cross-validation (LOO-CV) procedure. In this method, each observation was iteratively excluded from the dataset and used as a test case, while the remaining data were used to build the model. The percentage of correct classification for each species was then calculated by comparing the predicted species identity to the true identity of each observation. This approach provides an estimate of the model’s ability to correctly assign individuals to their respective species based on wing morphometry.

### ﻿Taxonomic changes

Morphological terminology follows Michener (2007). The following abbreviations are used in the species descriptions: A = antennal segments, S = metasomal sterna, and T = metasomal terga. Specimens were measured using a Zeiss SteREO Discovery.V8 binocular coupled with a Zeiss objective Plan Apo S 1.0 × FWD 60 mm. The scale of the binocular was calibrated using the same calibrated scale as for geometrics morphometrics at the lowest zoom. All the measurements were then taken at the same zoom for all the specimens. Ratio measurements of the labrum are given using the following format “width:length” where the width is measured from the right side (near the right compound eye, when viewed frontally) to the left side of the labrum (near the left compound eye, when viewed frontally) while the length is measured from the base (at the junction with the clypeus) to the apex of the labrum. Both width and length were measured on the central axis of the labrum. Length of the specimens was measured with the same equipment in profile view from the apex of the clypeus to the apex of the last tergum. Interalar width was measured with the same equipment by measuring the shortest distance between the inner margins of the tegulae.

Geometric morphometric analyses were conducted on the wings of the males of *A. amymone* and *A. bouwmani*, using the same method as for the other species (*vide supra*) within the framework of the synonymy between these two species. Nine males of *A. amymone* and 20 males of *A. bouwmani* were used for these analyses, all the specimens come from the island of Sumatra, in Indonesia and are hosted in the RMNH collection.

### ﻿Checklist of the Indo-Australian species of *Glossamegilla* and specimen photography

The update to the checklist of the Indo-Australian *Glossamegilla* was compiled by examining the RMNH collection, leading to the discovery of undescribed species, and integrating this information with published works (Smith 1857; 1860; Bingham 1896; Friese 1922; Lieftinck 1944, 1956). The RMNH collection was studied because it contains the collection of Lieftinck as well as part of the collection of R. Desmier de Chenon who collected in Indonesia, providing a nearly complete and comprehensive overview of the *Glossamegilla* known to the Indo-Australian Archipelago.

Photographs of the specimens (including the types of the new species found in the RMNH collection) were taken with using an Olympus E-M1 Mark II with a 60 mm macro lens. Additional close-ups were taken with the addition of a Mitutoyo M Plan Apo 10X infinity corrected objective lens in combination with an Olympus M.Zuiko 2x teleconverter lens, a 10-mm Kenko DG extension tube and a Meike MK-P-AF3B 10 mm extension tube. Photographs were stacked using Helicon Focus B (HeliconSoft, Ukraine) and plates were prepared in GNU Image Manipulation Program (GIMP) 2.10.32. Post-processing of some images was made in Photoshop Elements (Adobe Systems, USA) to improve lighting to highlight specific characters.

### ﻿Biogeography of the subgenus Glossamegilla

Label information of *Glossamegilla* species of the RMNH (entire collection), NHMUK (partim, type of *Anthophora amymone* Bingham, 1896 and type of Anthophora himalajensis var. pahangensis Meade-Waldo, 1914), MSNG (partim, type of *Anthophora proserpina* Gribodo, 1893, Anthophora violacea var. anthracina Gribodo, 1894 and *Anthophora tetrataeniata* Gribodo, 1894), OÖLM (partim, a single female of *Anthophora cyrtandrae* Lieftinck, 1944), and OUMNH (partim, a single female of *Anthophora himalajensis* Radoszkowski, 1882, the holotype of *Anthophora vigilans* Smith, 1860, the holotype of *Anthophora insularis* Smith, 1857, and three females of *A. insularis*) collections (see abbreviations above) were first collected and digitised (dataset available on RMNH repository of Bakker and Creuwels 2025). The specimens without coordinates provided on the labels were then georeferenced based on the location information available using Google Maps, Google Earth, OpenStreetMap, and FloodMap in order to identify the locations of as many specimens as possible. However, some localities could not be found, and were thus not georeferenced and do not appear in distribution maps.

Once the specimens were georeferenced, the distribution maps were produced using Rstudio 2024.09.1 and R v. 4.4.2 as well as the packages cowplot, googleway, ggplot2, ggrepel, ggspatial, libwgeom, sf, rnaturalearth, and rnaturalearthdata.

During the production of distribution maps, some species were grouped together based on their morphological similarities and thus their supposed affinities to have a better overview of the subgeneral biogeography. These groups are therefore: (i) *A. pendleburyi* group, composed of *A. pendleburyi* (Cockerell, 1929), *A. feronia* (Lieftinck, 1944), *A. celineae* sp. nov. and *A. suzanneae* sp. nov.; (ii) *A. cinnyris* group, composed of *A. cinnyris*, *A. insularis* and *A. pagdeni*; (iii) *A. sumatrana* group, composed of *A. sumatrana* and *A. jacobi*. Other species were not classified into groups as these are generally too dissimilar morphologically for this kind of grouping.

## ﻿Results

### ﻿Wing size analyses

**Figure 2.**
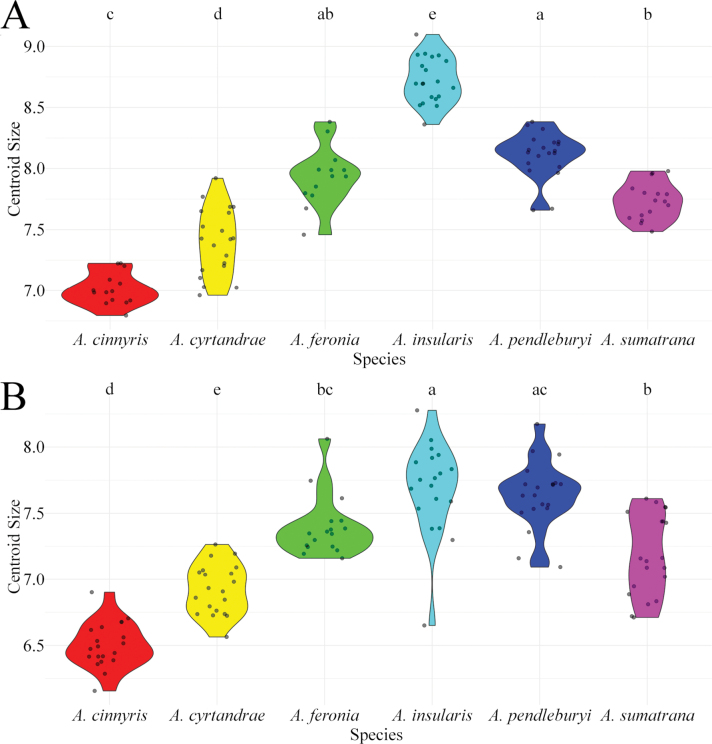
Distribution of the centroid sizes for the wings in both males and females. A. Violin plot for females; B. Violin plot for males. Letters above the boxplots indicate significant differences when the letters are different.

Regarding females, the six studied species significantly differ in wing size (p-value < 0.05; Table 2, Fig. 2A). When compared pair by pair, all the species significantly differ in wing size (all p-values < 0.001, Suppl. material 1), except for *A. pendleburyi* - *A. feronia* (p-value = 0.161) and *A. sumatrana* Lieftinck, 1956 – *A. feronia* (p-value = 0.100).

**Table 2.** Results of the ANOVA test for the differentiation of female’s centroid sizes from the subgenus Glossamegilla in Indonesia. Df is the Degree of Freedom. Sum Sq is the sum of squared differences between observed data and averages. Mean Sq is the sum of squared divided by the corresponding degree of freedom. F value is the ratio of variance explained to residual variance. ** indicates p < 0.01.

	Df	Sum Sq	Mean Sq	F value	P-value (>F)
Species	5	30.725	6.145	147.3	<0.001 **
Residuals	97	4.046	0.042		

For males, the six studied species significantly differ in wing size as well (p-value < 0.05; Table 3, Fig. 2B). When compared pair by pair, all the species significantly differ in wing size (all p-value < 0.05; Suppl. material 2) except for *A. pendleburyi* – *A. feronia* (p-value = 0.056), *A. sumatrana* – *A. feronia* (p-value = 0.127) and *A. pendleburyi* – *A. insularis* (Smith, 1857) (p-value = 0.992). *Amegilla sumatrana* – *A. cyrtandrae* (Lieftinck, 1944) (p-value = 0.023) and *A. insularis* – *A. feronia* (p-value = 0.013) show only weakly significant differences in terms of the size of the wings (Suppl. material 2).

**Table 3.** Results of the ANOVA test for the differentiation of male’s centroid sizes from the subgenus Glossamegilla in Indonesia. Df is the Degree of Freedom. Sum Sq is the sum of squared differences between observed data and averages. Mean Sq is the sum of squared divided by the corresponding degree of freedom. F value is the ratio of variance explained to residual variance. ** indicates p < 0.01.

	Df	Sum Sq	Mean Sq	F value	P-value (>F)
Species	5	20.276	4.055	61.330	<0.001**
Residuals	110	7.273	0.066		

### ﻿Wing shape analyses

**Figure 3.**
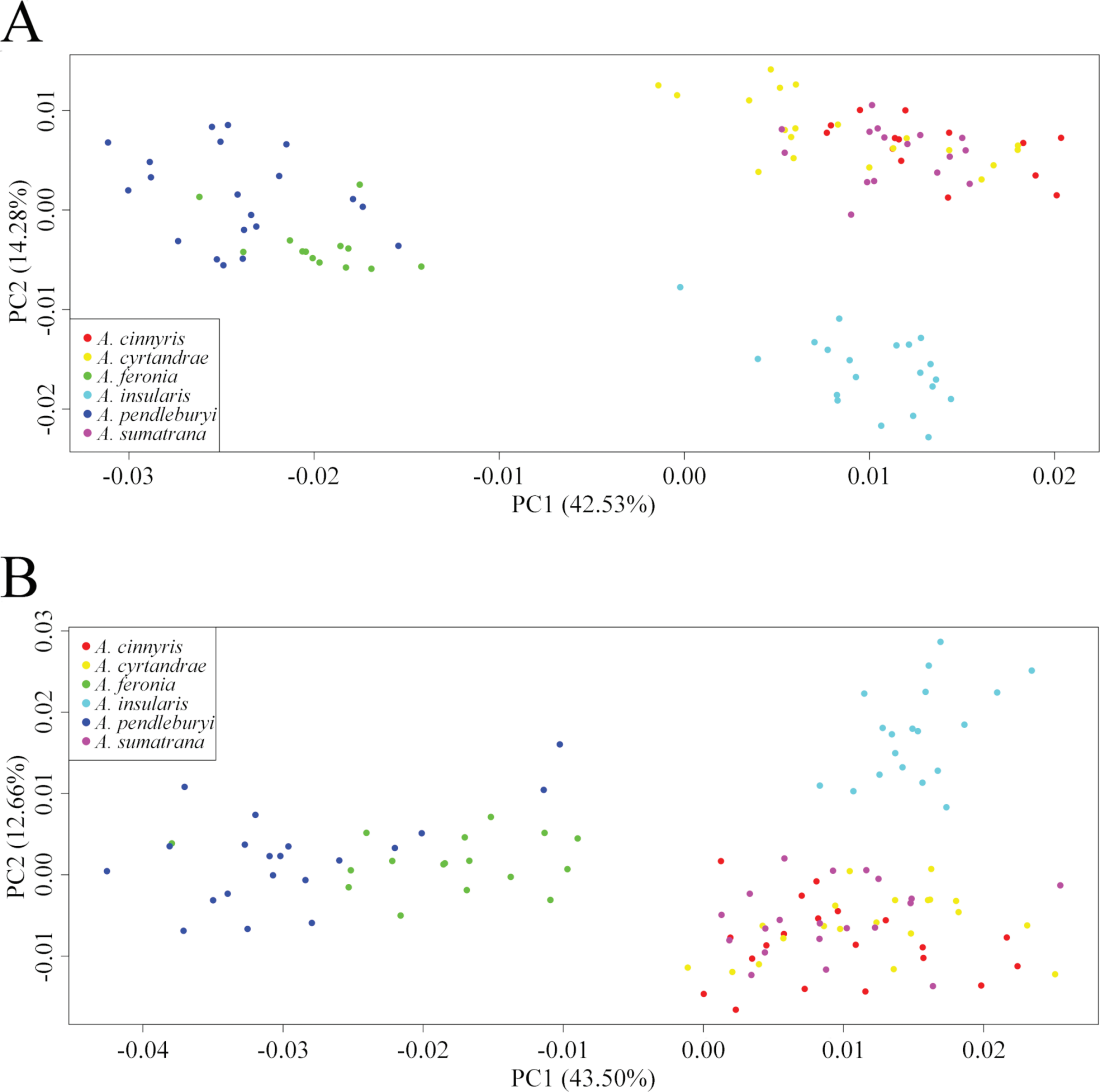
Ordination of the studied taxa along the two first axes of the Principal Component Analyses (PCA) (explaining 42.53% and 14.28% of the variance respectively for females and 43.50% and 12.66% for males). A. PCA for the females; B. PCA for the males.

The PCA plot for the females (Fig. 3A) shows the presence of three main clusters based on wing shape. One group, located in top-left of the plot consists of *A. feronia* and *A. pendleburyi*, two species that are morphologically similar outside of wing shape and closely aligned in terms of wing shape. A second group, located in top-right of the plot, includes more morphologically distinct species - *A. cinnyris* (Lieftinck, 1944), *A. cyrtandrae*, and *A. sumatrana* with considerable overlap between them. Finally, a third group, positioned in the bottom-right, is composed of only *A. insularis*.

**Figure 4.**
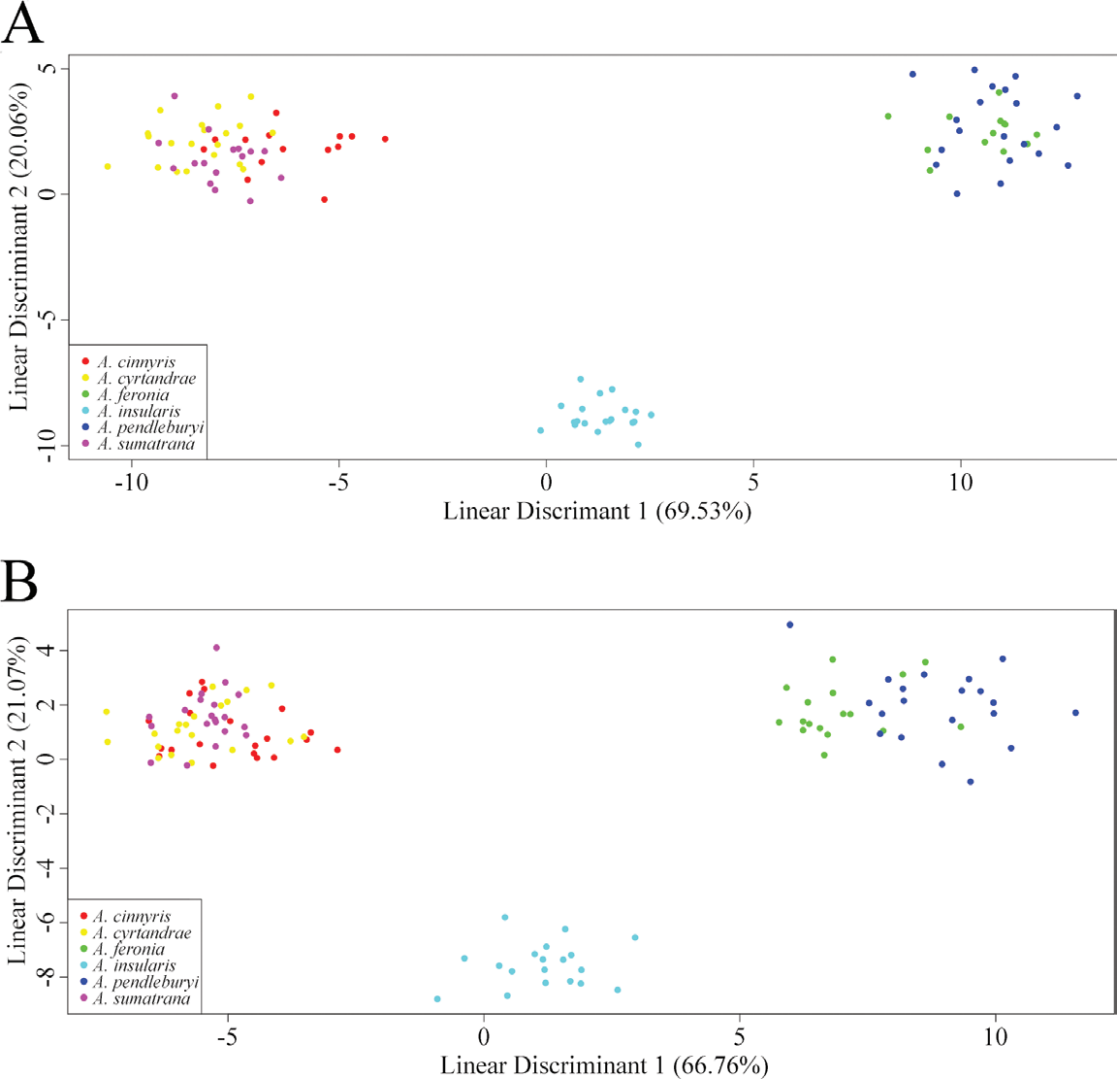
Ordination of the studied taxa along the two first axes of the Linear Discriminant Analyses (LDA) (explaining 69.53% and 20.06% of the variance respectively for females and 66.76% and 21.07% for males). A. LDA for the females; B. LDA for the males.

The Procrustes ANOVA for females shows significant differences in wing shape among the groups (p-value = 0.001, Table 4). Pairwise comparisons show that all groups are significantly different (all p-value < 0.04; Fig. 4A), with the exception of *A. cyrtandrae* and *A. sumatrana* (p-value = 0.181; Fig. 4A; Suppl. material 3). *Amegilla cinnyris* – *A. sumatrana* (p-value = 0.025) and *A. feronia* – *A. pendleburyi* (p-value = 0.034) show weakly significant differences in wing shape (Fig. 4A, Suppl. material 3). The accuracy of the LDA is 94.17%, meaning that 94.17% of specimens were correctly classified to their respective species based on wing shape (Suppl. material 4). This high accuracy suggests that the LDA effectively discriminates between species with minimal misclassification.

**Table 4.** Results of the Procrustes ANOVA for the differentiation of female’s wing shape from the subgenus Glossamegilla in Indonesia. Df is the Degree of Freedom. SS is the sum of squared differences between observed data and averages. MS is the sum of squared divided by the corresponding degree of freedom. Rsq is the proportion of total variance explained by the statistical model. F is the ratio of variance explained to residual variance. Z is the difference between the means of the two groups being compared divided by the standard deviation of this difference. ** indicates p < 0.01.

	Df	SS	MS	Rsq	F	Z	P-value
Species	5	0.037	0.007	0.596	28.669	9.414	0.001**
Residuals	97	0.025	0.000	0.404			
Total	102	0.062					

Similarly to the female analysis, the PCA plot for males shows three distinct clusters based on wing shape (Fig. 3B). First, in the left half of the plot, there is the group comprising *A. feronia* and *A. pendleburyi*, two morphologically similar species that show some overlap. Second, in the top-right of the plot, *A. insularis* forms a distinct cluster, separated from all other species. Finally, in bottom-right, a group of non-morphologically similar species – *A. cinnyris*, *A. cyrtandrae*, and *A. sumatrana* – is observed, with considerable overlap between them.

The Procrustes ANOVA for males shows significant differences in wing shape among groups (p-value = 0.001; Table 5). Similar to the females, pairwise comparisons show that all groups are significantly different (all p-value < 0.04; Fig. 4B), except for *A. cyrtandrae* and *A. sumatrana* (p-value = 0.112; Fig. 4B, Suppl. material 5). *Amegilla cinnyris* – *A. cyrtandrae* (p-value = 0.018), *A. cinnyris* – *A. sumatrana* (p-value = 0.033) and *A. feronia* – *A. pendleburyi* (p-value = 0.017) show weakly significant differences in wing shape (Fig. 4B, Suppl. material 5). The accuracy of the LDA is 87.07%, indicating that 87.07% of specimens were correctly classified to their respective species based on wing shape (Suppl. material 6). This suggests that the LDA made a relatively low proportion of misclassifications.

**Table 5.** Results of the Procrustes ANOVA for the differentiation of male’s wing shape from the subgenus Glossamegilla in Indonesia. Df is the Degree of Freedom. SS is the sum of squared differences between observed data and averages. MS is the sum of squared divided by the corresponding degree of freedom. Rsq is the proportion of total variance explained by the statistical model. F is the ratio of variance explained to residual variance. Z is the difference between the means of the two groups being compared divided by the standard deviation of this difference. ** indicates p < 0.01.

	Df	SS	MS	Rsq	F	Z	P-value
Species	5	0.048	0.010	0.547	26.520	8.651	0.001**
Residuals	110	0.040	0.000	0.453			
Total	115	0.089					

### ﻿Taxonomic changes

#### ﻿Diagnosis of the subgenus Glossamegilla

Taxon classificationAnimaliaHymenopteraApidae

Brooks, 1988

787EDBB5-1AC0-54B4-B48D-533E58CD5089

##### Diagnosis.

Male: The male is the most straightforward sex to determine the subgenus but the eighth sternum and the genitalia must be extracted to ensure correct identifications.

The revised diagnosis, based on a revision of Brooks’ (1988) characters, is: species restricted to south-east or eastern Asia, mainly distributed in the Indo-Malayan region (no species of this subgenus are found in Australia). The subgenus Glossamegilla can be distinguished from the subgenus Micramegilla Brooks, 1988 by (i) the long galea which reaches at least the middle of the midcoxa when resting, the galea is ≥ 3 × as long as the foretibia when measured from the apex to the maxillary palpus (*Micramegilla* have a moderately long galea which reach at most the anterior edge of the midcoxa when resting; the galea, with the same measurement method, is ≤ 2.5 × as long as the foretibia) and (ii) the S8 that is apicomedially emarginate between a pair of large obtuse lobes (while *Micramegilla* have a S8 apicomedially relatively narrowly emarginate between a pair of small obtuse lobes or with only a single median lobe). The subgenera *Glossamegilla* and *Micramegilla* also have different geographical distribution with *Glossamegilla* containing mainly Indo-Malayan species and *Micramegilla* containing mainly Palaearctic and Afrotropical species. These two subgenera only overlap in India, where the three Indian *Glossamegilla* have at least a length of 13 mm and entirely black haired terga or at least the anterior 1/2 orange haired but the Indian *Micramegilla* species have a length of ≤ 12 mm and terga with pale hair bands apically. *Glossamegilla* can be differentiated from the subgenera *Amegilla* Friese, 1897 sensu stricto, *Notomegilla* Brooks, 1988, *Asaropoda* Cockerell, 1926, *Zonamegilla* Popov, 1950, *Dizonamegilla* Brooks, 1988, and *Zebramegilla* Brook, 1988 by a generally wide apex of S8, that is also emarginate and bilobed with two blunt lobes, leading to a generally rectangular-shaped sternum in dorsal or ventral view (Fig. 6H) (while the other subgenera cited have a narrower apex of S8, that is either bilobed or rounded, leading to a triangle-shaped sternum in dorsal or ventral view). *Glossamegilla* can be differentiated from *Ackmonopsis* Brooks, 1988, *Aframegilla* Popov, 1950 and *Megamegilla* Brooks, 1988 by the absent gonostyli (Figs 6G, 9G, 10H, 11G) (while the three other subgenera have gonostyli that are various in size and shape but always present).

Usually, the long galea is a good character to determine the subgenus Glossamegilla, as the galea is ≥ 3 × as long as the foretibia (reaching to at least the middle of the midcoxa when resting), and this can help a lot for the determination as no other subgenera (especially in the Indo-Malayan region) have such a long galea.

**Female**: There is a lack of strong morphological diagnostic characters in the females, and the main characters used are based on colouration, which varies strongly. Michener (2007) described the subgenera of *Amegilla* as “largely indistinguishable in females, and in males differ from one another considerably less than do most subgenera of *Anthophora*”. He however stated that the high species richness of the genus would support the recognition of subgenera, and that names were available for use by workers. A complete morphologic and genetic revision of *Amegilla* subgenera should therefore be made in order to validate the concepts used by Brooks and find stronger characters to diagnose them.

The revised diagnosis, based on a revision of Brooks’ (1988) characters, is: Firstly, some species of the subgenus Glossamegilla have metallic hairs of various colours on the metasoma (e.g., *A. hanitschi* (Meade-Waldo, 1914) with the terga entirely and evenly covered by green metallic hairs), these species can be differentiated from the subgenera *Aframegilla* (partim), *Notomegilla*, and *Zonamegilla* (partim) by a long galea that reaches at least to the middle of the hind coxa when resting, the galea is ~3 × as long as the foretibia when measured from the apex to the maxillary palpus (while the three other subgenera have a short to moderate galea that reach at most the anterior edge of the hind coxa and is ≤ 2.5 × as long as the foretibia with the same type of measurement), the absence of paraocular marks (while these marks are present in the three other subgenera) as well as the biogeography (indeed, the *Glossamegilla* with metallic hairs like *A. hanitschi* are an Indo-Malayan group while *Aframegilla* is an African group, *Notomegilla* is an Australian group and *Zonamegilla* is a more widespread group distributed in the Indo-Malayan and Australian regions).

The second group of *Glossamegilla* does not have any metallic hairs on the terga. The latter either show pale hair bands that contrast black hairs on the tergal discs, are entirely covered by pale pubescence, or show another type of black and pale hairs mixing (Figs 5F, 7F, 8F, 9F, 10F, 11F, 12D, 13D, 14E, F, 17F, 18F, 19F, 20F, 21F, 22F, 23F, 24F). The pale hairs can vary from white (e.g., *A. sumatrana* and *A. jacobi* (Lieftinck, 1944) (Figs 21F, 24F)) to bright orange (e.g., *A. feronia*, Fig. 20F). In this group, the paraocular marks can be either absent or present but the maxillary palpi always have six segments.

**Figure 5.**
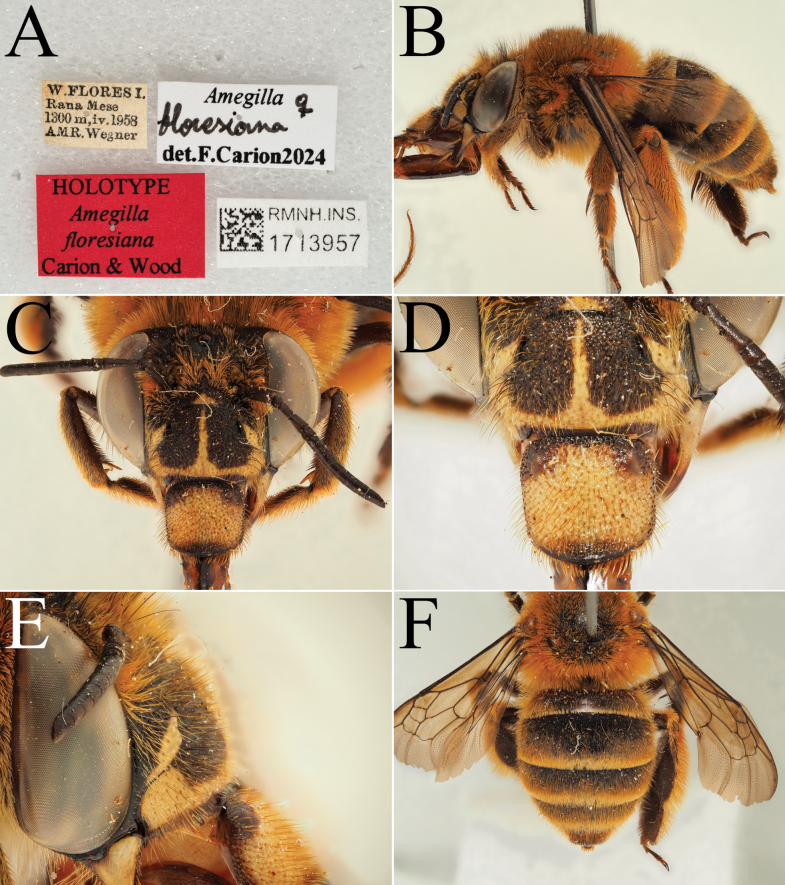
Holotype of *A. floresiana* Carion & Wood, sp. nov. (RMNH). A. Labels of the specimen; B. Habitus in profile view; C. Face in frontal view; D. Labrum in ventral view; E. Protuberance of the clypeus in profile view; F. Terga in dorsal view.

**Figure 6.**
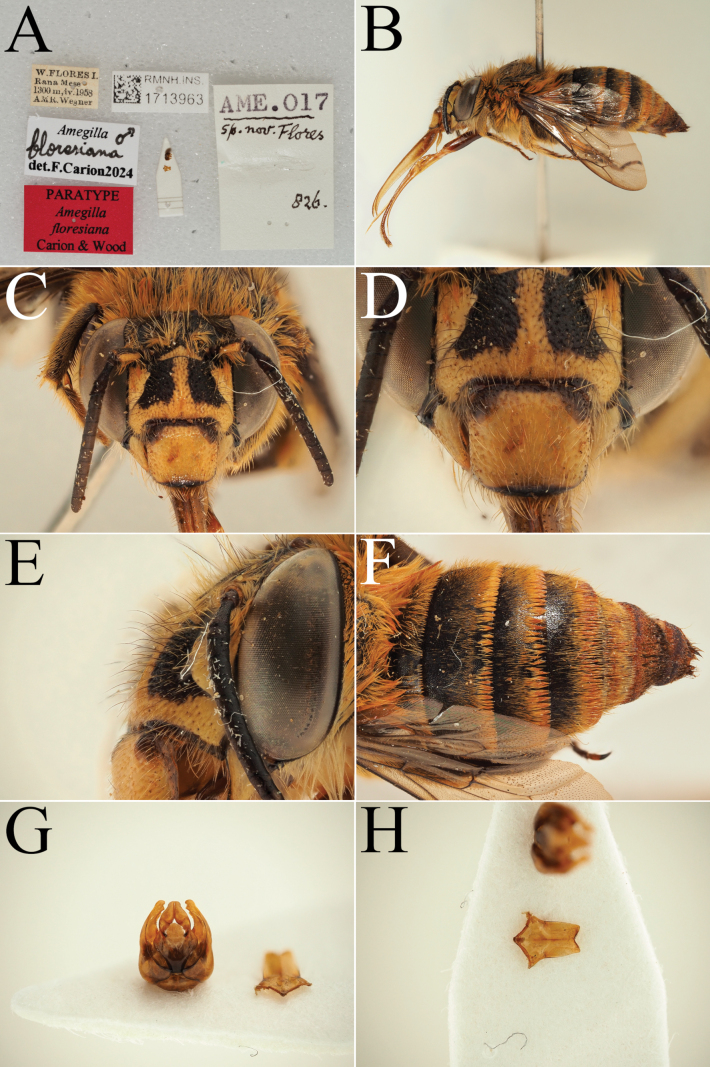
Male paratype of *A. floresiana* Carion & Wood, sp. nov. (RMNH). A. Labels of the specimen; B. Habitus in profile view; C. Face in frontal view; D. Labrum in ventral view; E. Protuberance of the clypeus in profile view; F. Terga in dorsal view; G. Genitalia in frontal view; H. Sterna 7 in dorsal view.

**Figure 7.**
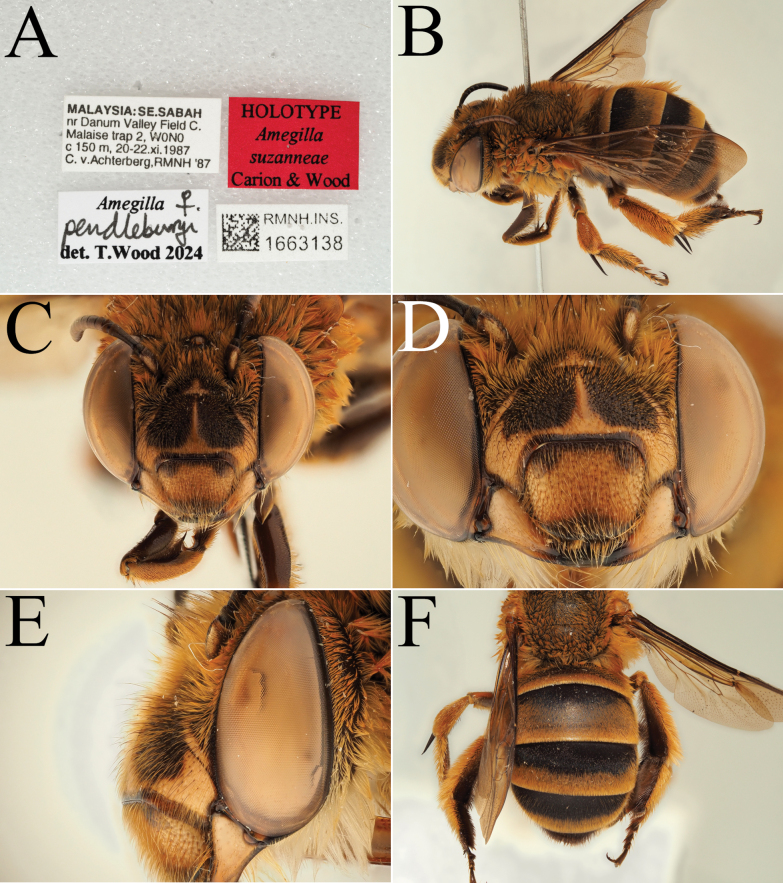
Holotype of *A. suzanneae* Carion & Wood, sp. nov. (RMNH). A. Labels of the specimen; B. Habitus in profile view; C. Face in frontal view; D. Labrum in ventral view; E. Protuberance of the clypeus in profile view; F. Terga in dorsal view.

**Figure 8.**
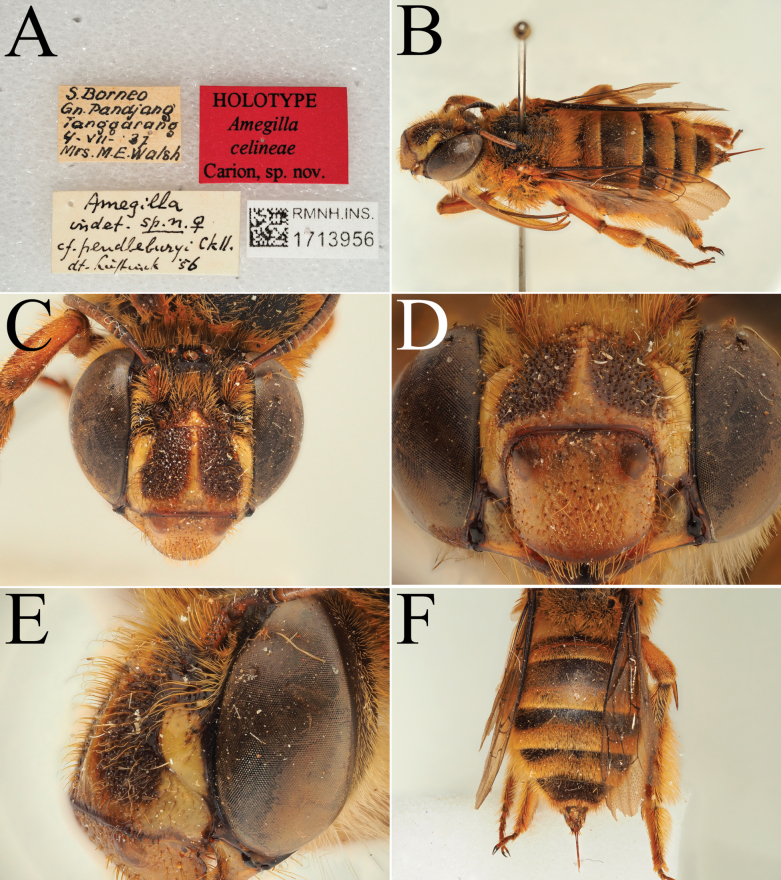
Holotype of *A. celineae* Carion, sp. nov. (RMNH). A. Labels of the specimen; B. Habitus in profile view; C. Face in frontal view; D. Labrum in ventral view; E. Protuberance of the clypeus in profile view; F. Terga in dorsal view.

**Figure 9.**
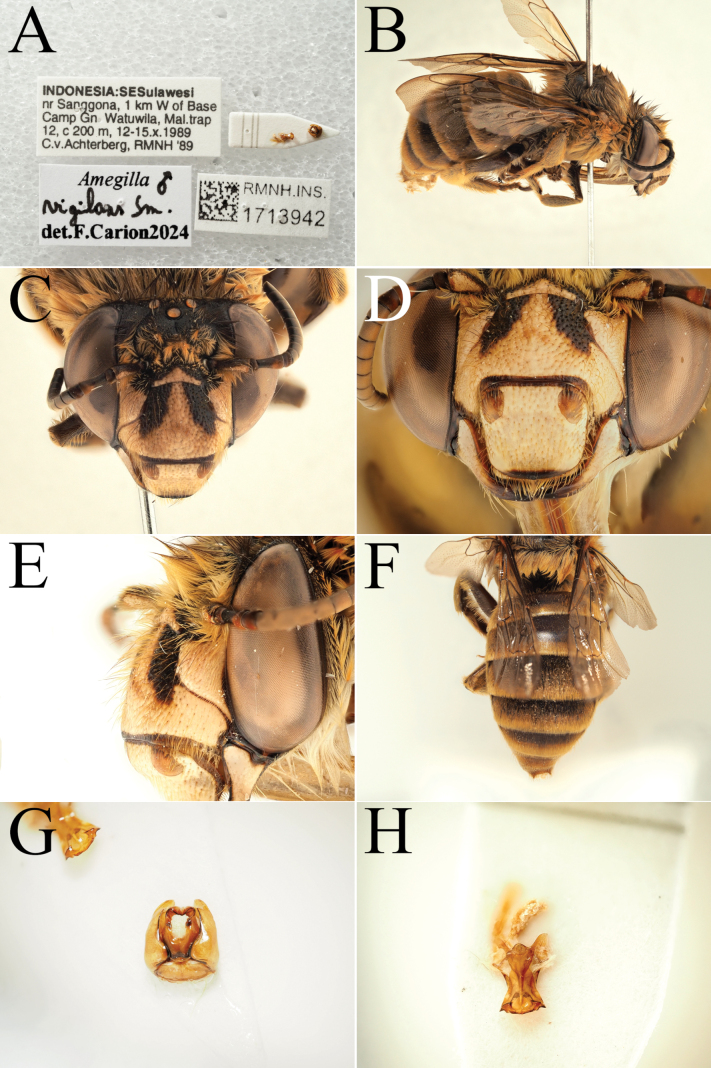
Pictures of the male of Amegilla (Glossamegilla) vigilans (Smith, 1860) (RMNH). A. Labels of the specimen; B. Habitus in profile view; C. Face in frontal view; D. Labrum in ventral view; E. Protuberance of the clypeus in profile view; F. Terga in dorsal view; G. Genitalia in frontal view; H. Sterna 7 and 8 in dorsal view.

**Figure 10.**
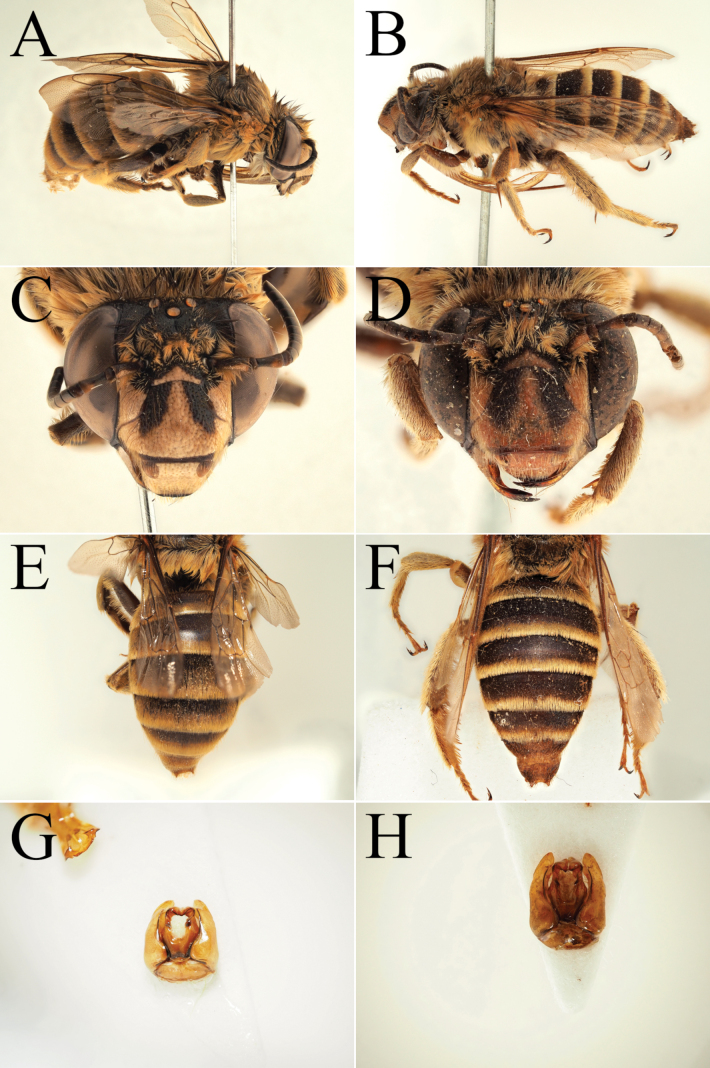
Morphological comparison between the male of both colour forms of Amegilla (Glossamegilla) vigilans (Smith, 1860) (RMNH). A., C, E, G *Amegilla vigilans* male pale form. B, D F, H *Amegilla vigilans* male dark form. A., B Habitus in profile view. C, D Face in frontal view. E, F Terga in dorsal view. G, H Genitalia in dorsal view.

**Figure 11.**
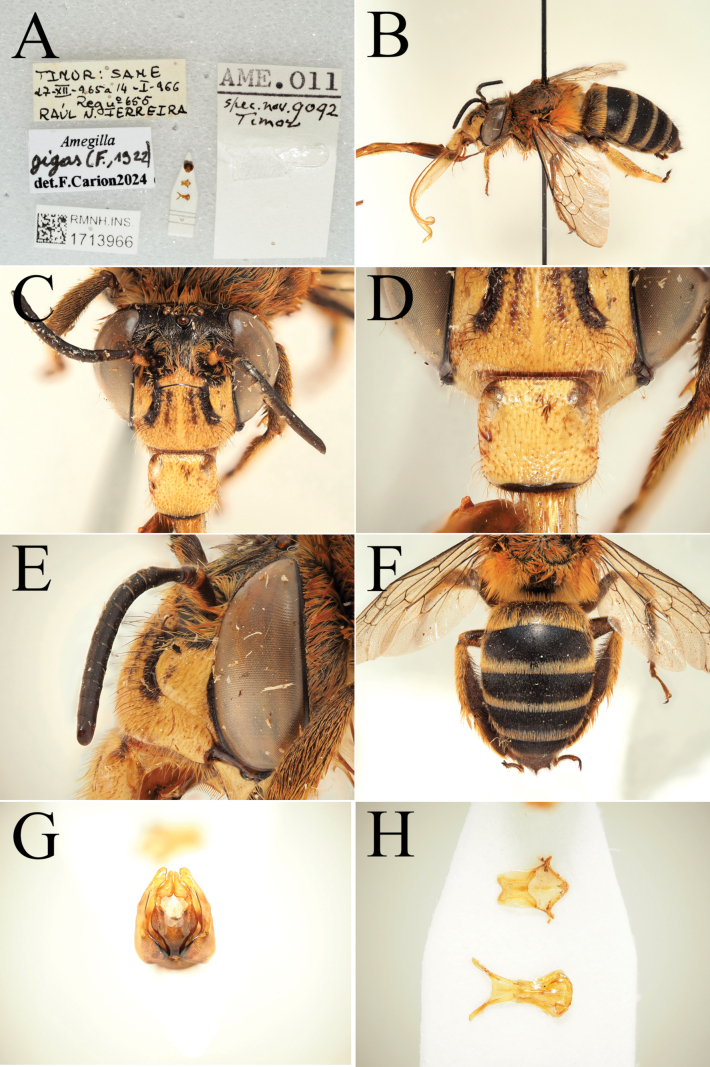
Pictures of the male of Amegilla (Glossamegilla) gigas (Friese, 1922) (RMNH). A. Labels of the specimen; B. Habitus in profile view; C. Face in frontal view; D. Labrum in ventral view; E. Protuberance of the clypeus in profile view; F. Terga in dorsal view; G. Genitalia in frontal view; H. Sterna 7 and 8 in dorsal view.

**Figure 12.**
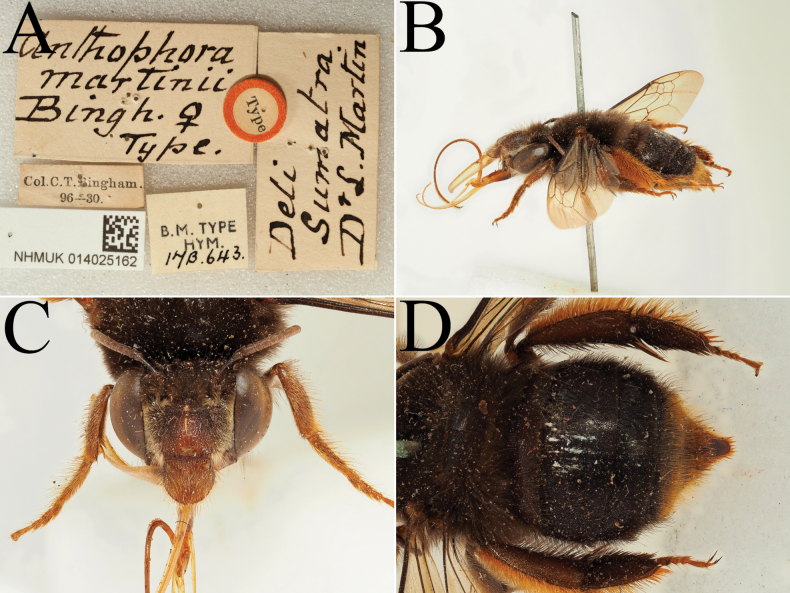
Holotype of *Amegilla amymone* (Bingham, 1896), initially published as *Anthophora amymone* Bingham, 1896 (NHMUK). A. Labels of the specimen; B. Habitus in profile view; C. Face in frontal view; D. Terga in dorsal view.

**Figure 13.**
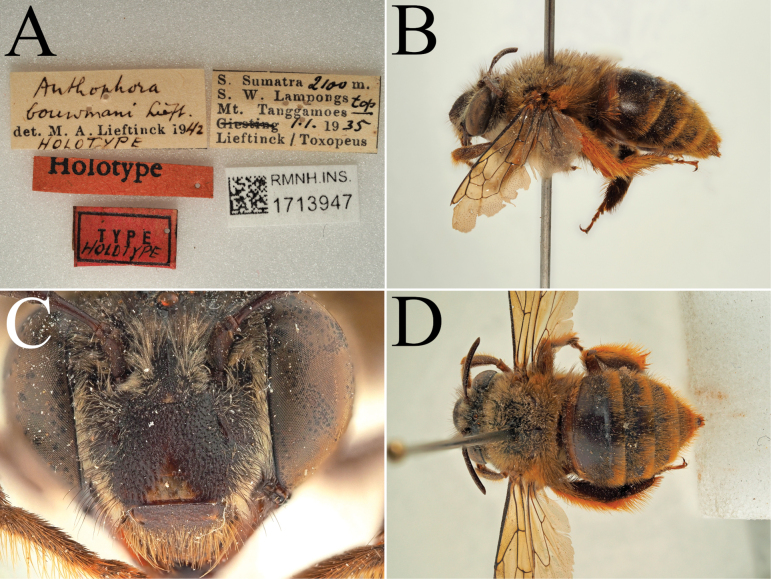
Holotype of *Amegilla bouwmani* (Lieftinck, 1944), initially published as *Anthophora bouwmani* Lieftinck, 1944 (RMNH). A. Labels of the specimen; B. Habitus in profile view; C. Face in frontal view; D. Terga in dorsal view.

**Figure 14.**
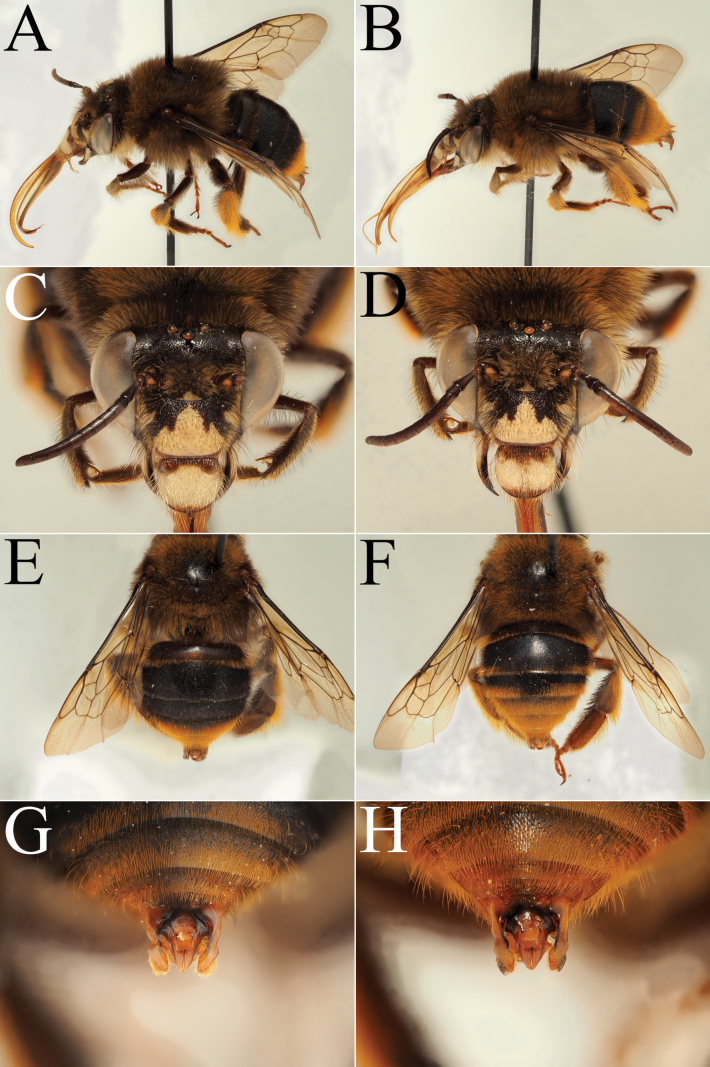
Morphological comparison between the males of Amegilla (Glossamegilla) bouwmani (Lieftinck, 1944) and Amegilla (Glossamegilla) amymone (Bingham, 1896) (RMNH). A, C, E, G. *Amegilla amymone* male. B, D, F, H. *Amegilla bouwmani* male. A, B. Habitus in profile view. C, D. Face in frontal view. E, F. Terga in dorsal view. G, H. Genitalia in dorsal view.

If pale paraocular marks are absent, *Glossamegilla* can be distinguished from *Asaropoda* by the biogeography (*Glossamegilla* have an Indo-Malayan distribution, mainly in the Indo-Malayan region while (*Asaropoda*) is restricted to Australia) and the pale clypeal marks that are an inverted T-shape or the clypeus can also be entirely to almost entirely black for *Glossamegilla* (Figs 7C, 12C, 13C) (while *Asaropoda* have a clypeus almost entirely pale, except for the small dark mark at the anterior tentorial pits). *Glossamegilla* can be differentiated from *Ackmonopsis* (partim), *Micramegilla* (partim) and *Amegilla* sensu stricto (partim) by the absence of hair bands on the apical margin of some terga and/or with some or all terga covered by dense and appressed pubescence (Figs 12D, 13D, 17F for example) (while the three other subgenera have all the terga with a hair band on the apical margin, these hair bands can be white to ochraceous). *Glossamegilla* can be differentiated from *Megamegilla* and *Ackmonopsis* by the pubescence of the mesosoma that can be ochraceous, fulvous, orange, bright orange, brown or even black with more or less black hairs intermixed (while the two other subgenera have a mesosoma with brown to orange hairs with more or less black hairs intermixed) (Fig. 7B) as well as terga with hair bands on the apical margins, basally covered by black pubescence on the discs, or entirely covered by pale pubescence of various colour on at least T2-4 (Fig. 7F) (while the two other subgenera have black hairs on almost all the terga except T3-5 that have portion of the apical margins with appressed white hairs). Finally, *Glossamegilla* can be distinguished from *Micramegilla*, *Amegilla* sensu stricto (partim), *Zebramegilla*, *Megamegilla* (partim) and *Aframegilla* (partim) by the Indo-Malayan distribution (this group of species are mainly distributed in India while the other subgenera are distributed in the Palaearctic and in Africa) and by the clypeus entirely black except sometimes with a small mark basomedially (while the other subgenera have more extended pale clypeal marks compound of an inverted T-shape or a median longitudinal line, only the species from Cape Verde Islands have an entirely black clypeus).

If pale paraocular marks are present (Figs 5C, 8C, 9C, 17C, 18C, 19C, 20C, 21C, 22C, 23C, 24C), the *Glossamegilla* can be separated from *Zebramegilla* (partim), the *Micramegilla* (partim) and the *Dizonamegilla* (partim) by their Indo-Malayan distribution while the other subgenera are distributed in Africa and Western Palaearctic. T1-2 can be either entirely black haired without pale hair bands on the apical margins nor appressed brown hairs (sometimes sides of the T2 can be covered by tuft of white hairs) (Fig. 21F) or banded with pale hairs on the apical margins (sometimes the T2 have poorly developed hair bands) (Figs 5F, 7F, 10F, 18F, 19F, 20F, 23F, 24F), sometimes most of T1-2 are covered by adpressed brown hairs that can be sometimes very sparse or absent medially.

If T1-2 are entirely black haired without pale hair bands on the apical margins nor appressed brown hairs (sometimes sides of the T2 can be covered by tuft of white hairs), *Glossamegilla* can be separated from the *Dizonamegilla* (partim) by the terga being entirely black haired, except for tufts of white hairs on the sides of T5.

If T1-2 are banded with pale hairs on the apical margins (sometimes the T2 have poorly developed hair bands and sometimes most of T1-2 are covered by adpressed brown hairs that can be sometimes very sparse or absent medially), the *Glossamegilla* can be differentiated from the *Zonamegilla* (partim) and *Zebramegilla* (partim) by (i) a body length of at least 12 mm (while the two other subgenera have a length of ≤ 9 mm), (ii) the mesosoma with ochraceous to bright orange hairs with more or less or without black hairs intermixed (Figs 5B, 7B, 10B, 18B, 19B, 20B, 23B, 24B) (while the two others subgenera have a mainly white haired mesosoma with some black hairs intermixed) and (iii) with pale hair bands on the apical margins of the terga that are generally brown (but can also be ochraceous to bright orange) while the discs is at least partially black haired or terga entirely covered by ochraceous to fulvous or brown pubescence that is sometimes denser apically, leading to slightly contrasting hair bands (Figs 5F, 7F, 8F, 9F, 10F, 17F, 18F, 19F, 20F, 22D, 23F, 24F) (while the two others subgenera have pale hair bands on the apical margins of the terga).

As previously said, the females are more difficult to determine at the subgenus-level as mainly colouration-based characters are used and these characters vary substantially. However, the biogeographic regions and the size of the galea (that reaches at least the middle of the hind coxa when resting, with the galea ~3 × as long as the foretibia when measured from the apex to the maxillary palpus) are powerful diagnostic characters.

#### ﻿ Amegilla (Glossamegilla) floresiana

Taxon classificationAnimaliaHymenopteraApidae

Carion & Wood sp. nov.

A9A7F5E0-9452-56A3-A367-3A42DBF0750B

https://zoobank.org/8A452C0C-D945-44A6-9331-F0BF8EF40198

Figs 5, 6

##### Type material.

***Holotype*: Indonesia** • ♀; West Flores, Rana Mese; 1300 m a.s.l.; Apr. 1958; A.M.R. Wegner leg.; RMNH, RMNH.INS.1713957. ***Paratypes*: Indonesia** • 5♀♀, 3♂♂; same collection data as for holotype; RMNH, RMNH.INS.1713958 to RMNH.INS. 1713965 • 1♀; Ruteng; 1200 m a.s.l.; 17 Feb. 1961; J.M.A. v. Groenendael leg.; RMNH, ZMA.INS.5187867.

##### Diagnosis.

Male: The male of *A. floresiana* sp. nov. is a species with contrasting pale hair bands on the metasoma (sometimes a little obscured by the surrounding pale pilosity) and the tergal discs usually partially covered by black pilosity. This produces an effect of hair bands that are poorly defined but clearly present. This species differs from the group of *A. jacobi*/*A. sumatrana* by the wider and more ochraceous to fulvous hair bands, the tergal discs being less covered by black pilosity than in these comparison species. *Amegilla floresiana* sp. nov. can be differentiated from the group of *A. feronia* and *A. cyrtandrae* by the larger size (~18 mm for *A. floresiana* sp. nov. while *A. feronia* and *A. cyrtandrae* are ~15–17 mm) and a more protuberant clypeus which in profile view equals or exceeds the width of the compound eye (while the clypeus is less protuberant in *A. feronia* and *A. cyrtandrae*, with a protuberance smaller than the width of the compound eye); the pale pubescence of mesosoma and metasoma also varies from ochraceous to fulvous for *A. floresiana* sp. nov. (while it varies from fulvous to bright orange for *A. feronia* and *A. cyrtandrae*). Finally, this species differs from *A. gigas* and the dark form of *A. vigilans* by the labrum slightly wider than long (in the two comparison species with the labrum as wide as long), a fine (not coarse) punctation of the clypeus with large interspaces of at least the diameter of a puncture (while the two comparison species have a coarse and not very sparse punctation with rather smaller interspaces of at most the diameter of a puncture but generally less), wider and very poorly defined hair bands with a small parts of the tergal discs with black hairs or no black hairs at all as well as, sometimes, a brighter pilosity (from ochraceous to bright orange-fulvous) (while *A. gigas* and the dark form of *A. vigilans* have narrower and more sharply defined hair bands with a larger black haired area on the discs that is always present as well as a less bright pilosity (from ochraceous to very slightly orange)).

Female: As for the male, the female of *A. floresiana* sp. nov. is a species with contrasting pale hair bands on the metasoma (sometimes a little obscured by the surrounding pale pilosity) and the tergal discs partially covered by black pilosity. This produces an effect of hair bands that are poorly defined but clearly present. This species differs from the group of *A. jacobi*/*A. sumatrana* by the wider and more colourful hair bands, the tergal discs being less covered by black pilosity than in these species. *Amegilla floresiana* sp. nov. can be differentiated from the group of *A. feronia*, *A. cyrtandrae* and *A. suzanneae* Carion & Wood, sp. nov. by the larger body size (~17–18 mm for *A. floresiana* sp. nov. while *A. feronia*, *A. cyrtandrae* and *A. suzanneae* sp. nov. are ~13–16 mm) and a more protuberant clypeus which in profile view equals or exceeds the width of the compound eye (while the clypeus is less protuberant in *A. feronia* and *A. cyrtandrae*, with a protuberance smaller than the width of the compound eye). *Amegilla floresiana* sp. nov. differs from *A. gigas* by a sharper and more protruding clypeal carina that thus does not appear flat (while in *A. gigas* the clypeal carina is very flat, not sharp at all, and does not protrude very much or not at all), more extended and ivory-yellow to yellow clypeal marks and entirely punctuate paraocular areas (while *A. gigas* have less extended and yellowish, tending to dark yellow, pale clypeal marks, the face is mainly dark; *A. gigas* have restricted ivory-white spots on the paraocular areas as well as a clear shining area between the punctation of the paraocular areas and the transition with the clypeus). Finally, *A. floresiana* sp. nov. can be distinguished from the dark form of *A. vigilans* by rather poorly defined hair bands at the apex of the terga that are wider than for *A. vigilans* with pale pilosity covering the majority of the discs of the terga and almost the entirety of T4 (while *A. vigilans* have rather sharply defined and narrower hair bands with the discs of the terga more covered by black pubescence), the pale clypeal marks less extended and ivory-yellow to yellow (while *A. vigilans* have more extended yellow-orange pale clypeal marks), a finer punctation of the clypeus with larger interspaces approximately the diameter of the punctures (*A. vigilans* have a clypeus with a coarser, less defined punctation with smaller interspaces of generally ≤ 1/2 the diameter of a puncture), paraocular areas of the same colour than the clypeal marks but the colouration is less extended than in *A. vigilans* (*A. vigilans* also has the paraocular areas of the same colour than the clypeal marks but the colouration is more extended) and a pubescence usually brighter, more fulvous to orange but can sometimes also be ochraceous, similarly to *A. vigilans* which have a pubescence ochraceous to slightly fulvous.

##### Description.

**Male**: Length of 15.5–16 mm. Interalar width of ~3.3–3.8 mm (shorter distance between the base of the tegulae).

***Head***: Labrum rectangular, slightly wider than long (17:16–19:18, 1.7 × 1.6 mm – 1.9 × 1.8 mm, thus sometimes hardly visible, can be considered square without measurements) with two slightly protruding brown areas at the base, remaining labrum entirely yellow except for the black transverse carina at the apex of the labrum and a brownish to black narrow bands at the base. Labrum entirely, evenly, and densely punctate with interspaces of generally ≤ 1/2 the size of a puncture but sometimes more. Labrum entirely covered by not very dense but very pale ochraceous pubescence approximately as long as the diameter of an ocellus. Mandibles bidentate, both teeth blunt, not strongly sharp, the secondary tooth sometimes less developed but still visible. Mandibles ivory-yellow on the basal 1/2 and deep brown on the apical 1/2. Clypeal marks variable, clypeus either more yellow than dark or more dark than yellow but the two subrectangular black clypeal marks always present. The pale clypeal marks on the clypeus are compound of a yellow inverted T-shape that is more extended on the area between the paraocular area and the labrum. Clypeus entirely, finely, and relatively densely punctate with interspaces generally of approximately the diameter of a puncture. Clypeal carina slightly protruding but not very much, rather sharp and impunctate) vertical bar of the yellow T). Clypeus entirely, evenly, and sparsely covered by a mixture of ochraceous and black pubescence with a length of at least the diameter of an ocellus. Paraocular areas slightly protruding and yellow. Paraocular areas evenly and relatively densely punctate with interspaces of at most the diameter of a puncture, presence of an impunctate area on the side of the area, near the margin with the clypeus (area of the protrusion) but separated from this margin by some punctures. Clypeus not strongly protuberant, in lateral view protuberance of the apical margin of the clypeus of approximately the diameter of the eye (sometimes less). Scape anteriorly yellow and posteriorly black except a narrow orange band at the apex of the scape (sometimes restricted to a small paler area or even absent). Scape entirely covered by an ochraceous to slightly fulvous pubescence approximately as long as the width of the scape and that is denser on the sides. A2 basally black and apically reddish. Anterior surface of A3 basally black (2/3) and apically reddish (1/3), posterior surface black. A4-12 paler on the anterior surface (reddish or brownish) and black posteriorly, sometimes the difference of colouration is not well marked to absent. A3-12 entirely and evenly covered by very small, hardly visible, white pilosity. A2 sparsely covered on the sides by ochraceous-fulvous or black pubescence that is longer than the pubescence of the next articles. A3 longer than wide, approximately as long as 4+5. A4 shorter than wide. A5-12 square (as long as wide) or slightly rectangular (slightly longer than wide). A13 not cylindrical, obliquely truncated at the apex. Frons to gena black (except a yellow triangle or band at the margin of the frons) and rather not evenly covered by an ochraceous to fulvous pubescence of various length that is denser near the apical insertions but not very dense in general. Some long black hairs intermixed in the pale pubescence near the ocelli. Presence of a relatively deep depression that goes from the middle ocellus to the pale mark of the frons, width slightly more than the diameter of a surrounding puncture, the carina hardly showing trough the pubescence. Genae with very long ochraceous hairs.

***Mesosoma***: Mesosoma entirely covered by ochraceous to fulvous pubescence, with some to many black hairs intermixed in the pilosity of the dorsum. Scutum and scutellum finely and closely but not entirely punctate with interspaces generally ≤ 1/2 the diameter of a puncture. Centre of the scutum with a wide impunctate area with only a few punctures in it. Centre of the scutellum with a sharp carina that goes from the base to ~2/3 of the scutellum, base of the carina surrounded by a small impunctate area. Cuticle of the mesosoma deep black under the pilosity. Tegulae hyaline deep brown, sparsely and shallowly punctate with interspaces of at least one diameter of a puncture. Tegulae relatively densely and entirely or partially covered by ochraceous to fulvous pubescence. Wings relatively sparsely covered by dark hairs on the veins and inside the cells with a denser pilosity on the veins (most parts of the cells covered by pubescence). Apex of the wings covered with very sparse and dark brown protuberances that are not similar to the hairs. Wings translucent but slightly yellow. Cuticle of the legs dark brown to black. All legs exteriorly covered by an ochraceous to fulvous pubescence and interiorly covered with a slightly darker to black pubescence, generally the hind basitarsi exteriorly have a mix of pale and dark hairs (proportions of both variable). Tarsal claws of all the legs bidentate with subapical tooth located medially. Tarsal claws very sharp, the secondary claws are very long, 2/3 the length of the main one, sometimes as long. Main claws arched, without any tubercles on its ventral surface. Arolium between claws absent. Apex of mid tibia with one long and thick deep brown spine which is crenulate on two sides; the spine is slightly curved at the apex. Apex of hind tibia with two long and thick deep brown spines which are crenulate on both sides; spines slightly curved at the apex.

***Metasoma***: At least the sides of T1 covered by long ochraceous to fulvous pubescence, sometimes also the centre, which are slightly denser on the apical margin, forming a not very contrasting hair band. Disc of T2-4 basally covered by black pubescence, the remainder covered by ochraceous to fulvous pubescence, forming a wide and contrasting (but poorly defined) hair band or T2-4 entirely covered by the pale pubescence that is denser apically, forming a contrasting hair band (sometimes a mix of the two depending on the tergum). T5 entirely covered by pale pubescence. T6 entirely covered by black pubescence. T1-5 entirely, evenly, and sparsely punctate with shallow punctation and interspaces of at least the diameter of one puncture. T6 entirely and evenly punctate but with a deeper and denser punctation with interspaces of less than the diameter of a puncture. T7 (pygidial plate) with two spine-like structures widely separated by a flat (non-curved) area. T1-5 with a black integument on the disc, sometimes the apical margins are dark brown. T6-7 with a brown to dark brown integument. S5-7 banded with ochraceous pubescence, sometimes S1-4 also banded with the same type of pubescence. Integument of all sterna generally brown to dark brown, sometimes with an ochraceous apical margin, sometimes all terga with a black to slightly brownish integument. S1-2 mainly impunctate with only some small areas with very close and shallow punctation on the centre of the sterna. Sides of S3 densely and shallowly punctate with interspaces of approximately the diameter of a puncture while the centre and the apical margin is very sparsely punctate with only a few punctures. Sides and apical margin of the S4 densely and shallowly punctate with interspaces of approximately the diameter of a puncture while the centre and the apical margin is very sparsely punctate with only a few punctures. S5-7 entirely, densely, and shallowly punctate with interspaces of approximately the diameter of a puncture. S8 with some very small yellow hairs on the base, spine of the spiculum rounded and not curved (Fig. 6G, H). Gonocoxae mainly glabrous with only a few sparse white hairs. Intern face of the apex of gonocoxae with a curved depression, the apex rounded. Genitalia dark brown to black at the base of the valves, orangish to yellow elsewhere (Fig. 6G).

**Female**: Length of 16–17.5 mm. Interalar width of ~3.8–4.3 mm (shorter distance between the base of the tegulae).

***Head***: Labrum square, as wide as long (1:1, 1.9 × 1.9 mm – 2 × 2 mm), with two slightly protruding deep brown to black areas at the base (sometimes paler on the centre), remainder of the labrum yellow (generally darkened) except for the black transverse carina at the apex preceded by a brownish area variable in length but not taking the all width of the labrum as well as a narrow black band at the base that can vary in width. Labrum entirely, evenly, and densely punctate with interspaces generally approximately or less than the diameter of a puncture (the pale colouration of the labrum makes it sometimes hard to see), sometimes the interspaces are wider (one specimen of the type series with wider interspaces). Labrum entirely covered by relatively dense fulvous pubescence, the setae are approximately or slightly longer than the diameter of an ocellus. Mandibles generally bidentate (sometimes not, on one specimen of the type series) and blunt (rounded appearance), ivory-yellow to yellow on the basal 1/2 and deep brown on the apical 1/2, ending in black at the apex (sometimes only the basal 1/3 pale and the remainder dark). Clypeus mainly black to slightly reddish with dark clypeal marks compound of two large sub-rectangular areas, the pale clypeal marks on the clypeus are resumed to a yellow inverted T-shape that is more extended on the area between the paraocular area and the labrum. The total surface of the pale clypeal marks varies a lot, sometimes not very extended but still present. Clypeus entirely, slightly coarsely and densely punctate with interspaces of at most the diameter of a puncture. Clypeal carina reduced, not on all the length of the clypeus, and only slightly protruding, rather blunt and impunctate. Clypeus entirely, evenly, and relatively densely covered by a fulvous pubescence of variable length. Paraocular slightly protruding and yellow only on the apical 1/2, near the clypeus. Paraocular areas evenly, entirely, and densely punctate with interspaces of ~1/2 the diameter of a puncture (sometimes difficult to see on the pale part due to the colouration. Clypeus not strongly protuberant, in lateral view protuberance of the apical margin of the clypeus less than the diameter of the eye. Scape black except on the apex of the anterior face that is slightly reddish. Scape anteriorly with a carina on the apical 1/2 to apical 1/3 of the scape. Sides of the scape covered by fulvous pubescence approximately as long as the width of the scape, less dense to absent on the anterior and posterior surface. A2 black at the base and more reddish at the apex. A3-12 slightly reddish to deep brown anteriorly and black posteriorly. Sometimes apex of A3-4 with a narrow, ivory-white line. A3-12 entirely and evenly covered by very small, hardly visible, white pilosity. A2 anteriorly and apically with a small tuft of very small (but larger than the pilosity on the other articles) black hairs, hairs sometimes ochraceous to fulvous. A3 longer than wide, equal to slightly longer than 4+5+6. A4-5 shorter than wide. A6-11 squared, approximately as long as wide. A12 not cylindrical, obliquely truncated at the apex. Frons to gena black (except an ivory-yellow to yellow-orange triangle, both shape and surface of triangle variable) and rather evenly covered by an ochraceous to fulvous pubescence of various length that is denser near the antennal insertions. Many long black hairs intermixed in the pale pubescence near the ocelli. Presence of a relatively shallow depression that goes from the middle ocellus to the pale mark of the frons, width of ~1/2 the diameter of a surrounding puncture, the depression hardly showing trough the pubescence. Genae with very long white to pale ochraceous hairs.

***Mesosoma***: Mesosoma entirely covered by an ochraceous to fulvous pubescence, with many black hairs intermixed in the pilosity of the dorsum. Scutum and scutellum entirely, finely, and closely punctate with interspaces generally ≤ 1/2 the diameter of a puncture. Presence of a depression, the width less or approximately the diameter of a surrounding puncture, on the centre of the scutum that does not go to the end of the scutum. Scutum with two small carinae, one on each side of the scutum, near the tegulae. Centre of the scutellum with a rather sharp carina that goes from the base to ~2/3 of the scutellum, base of the carina not surrounded by a small impunctate area but the punctation is less dense. Cuticle of the mesosoma deep black under the pilosity. Tegulae hyaline brown to hyaline deep brown, sparsely and shallowly punctate with interspaces of at least one diameter of a puncture. Tegulae sparsely and entirely covered by fulvous pubescence with sometimes some black hairs intermixed. Wings relatively sparsely covered by dark hairs on the veins and inside the cells with a denser pilosity on the veins (most parts of the cells covered by pubescence), some veins at the apex and at the base glabrous. Apex of the wings covered with very sparse and dark brown protuberances that are not similar to the hairs. Wings translucent but slightly yellow. Cuticle of the legs dark, dark brown to black but never orange. All legs exteriorly covered by an ochraceous to fulvous pubescence and interiorly covered with a slightly darker to black pubescence except on the exterior surface of the hind tibia that is covered by a mix of dark and pale pubescence. Tarsal claws of all the tarsi bidentate with the second tooth situated on the middle of the main claw. Tarsal claws very sharp but small, ~1/3 the length of the main one (sometimes 1/2 the length of the main one). Apex of mid tibia with one long and thick deep brown spine which is crenulate on two sides; the spine is curved at the apex. Apex of hind tibia with two long thick black spines which are crenulate on both sides; spines curved at the apex.

***Metasoma***: Disc of T1 entirely covered by long ochraceous to fulvous pubescence that is denser on the side and more sparsely distributed on the centre of the tergum, apical margin covered by denser and shorter pubescence of the same colour forming a slightly contrasting hair band. Discs of T2-3 at least basally and centrally covered by black pubescence while the remaining discs (sides) and the apical margin are covered by ochraceous to fulvous short pubescence that is denser apically, forming a contrasting but poorly defined hair band. T4-5 (sometimes also T3) entirely covered by ochraceous to fulvous pubescence, T5 with black hairs intermixed (on the discs generally but also sometimes a black hair band is present). T1-5 entirely, evenly, and sparsely punctate with shallow punctation and interspaces of at least the diameter of one puncture, apex of apical margin impunctate. Integument of the terga mainly black but the apical margin sometimes slightly paler, brown (hardly visible through the pubescence) it is therefore probable that the apical margins were originally pale in fresh specimen (similarly to *A. pendleburyi* and *A. feronia*) but the integument decoloured with the time. Sterna 1–6 with hair band that are denser from S1 to S6, all hairs ochraceous except on S6 where the hairs are darker, black (sometimes other hair bands also darker). Integument dark brown to black for all the sterna except S1-4 that are sometimes paler, more deep brown. Apical margin of sterna sometimes paler (ochraceous to brown). S1-3 with very sparse punctation with interspaces of more than the diameter of a puncture and wide impunctate areas. Apical margin and sides of S4 densely, entirely, and evenly punctuate with interspaces of at most the diameter of a puncture, sternal disc with sparser punctation (interspaces more than the diameter of a puncture). S5-7 densely, entirely, and evenly punctuate with interspaces of at most the diameter of a puncture.

##### Remarks.

As the description of the male is based on three specimens, workers should consider that intraspecific variation could occur for this species, especially for some characteristics like the clypeal marks and the colouration, or even the morphology of the mandibles. The description of the female is more precise for this species as seven specimens were studied but further intraspecific variation could exist.

One specimen from the island of Sumbawa (not considered to be part of the type series, hosted in RMNH) morphologically differs from the typic series of *A. floresiana* sp. nov. This specimen was previously labelled as *A. feronia*. However, this specimen does not correspond exactly to either *A. feronia* or *A. floresiana* sp. nov. Due to the more probable biogeography and some common morphological traits, this species was regarded as A. aff. floresiana sp. nov. but cannot currently be considered as a true *A. floresiana* sp. nov. because of the morphological and biogeographic differences. This specimen would require more examinations in order to accurately be associated with a species and check its relationship with both *A. feronia* and *A. floresiana* sp. nov.: we leave it undescribed until additional specimens can be located, and it is not included in the following identification key.

##### Etymology.

The combination of the island name of Flores, as the type series is the first species of *Glossamegilla* known from the island of Flores, with the Latin suffix -*iana* indicating a collection of things related to a specific place, hence *floresiana* sp. nov.

##### Distribution.

Species restricted, with the current information, to the island of Flores. The A. aff. floresiana is only known on the island of Sumbawa.

#### ﻿ Amegilla (Glossamegilla) suzanneae

Taxon classificationAnimaliaHymenopteraApidae

Carion & Wood sp. nov.

28335E84-21E7-5182-876D-07FE369BB017

https://zoobank.org/D5E11B6B-5F75-447E-A1F1-C8439917A0D5

Fig. 7

##### Type material.

***Holotype*: Malaysia** • ♀; South East Sabah, nr Danum Valley Field C.; 150 m a.s.l.; 20–22 Nov. 1987; C.v. Achterberg leg.; Malaise trap; RMNH, RMNH.INS.1663138. ***Paratypes*: Malaysia** • 1♀; Sabah, Kinabalu Park Head Q.; 1600 m a.s.l.; 8–11 Mar. 1987; C.v. Achterberg leg.; Malaise trap; RMNH, RMNH.INS.1663139 − 1♀; Sarawak, Lio matu [Lio Mato, also known as Lio Matoh], Baram River; 25 Oct. 1920; J.C. Moulton leg.; RMNH, RMNH.INS.1713955.

##### Diagnosis.

Male: Unknown.

Female: *Amegilla suzanneae* sp. nov. is morphologically close to *A. pendleburyi* but differs by a less protruding clypeal carina (while the clypeal carina protrudes more in *A. pendleburyi*), the scape anteriorly dark brown or ivory-white but never orange (*A. pendleburyi* with scape anteriorly orange) and the presence of black hairs on tergal discs, placing it in the group of species displaying hair bands. In the key, *A. suzanneae* sp. nov. is artificially put close to *A. feronia* due to the presence of hair bands, the usually paler hind tibiae, and the fact that they are both closely morphologically related to *A. pendleburyi*. *Amegilla suzanneae* sp. nov. differs from *A. feronia* by the pale clypeal marks ivory-yellow to yellow (while these marks are clearly yellow or even slightly orange to orange on *A. feronia*), the scape ivory-yellow to yellow also but sometimes dark brown (*A. feronia* have anteriorly orange scape) and the hair bands less sharply defined basally with the pale pilosity more extended and the black pilosity more restricted (*A. feronia* have wider black-haired areas on the tergal discs as well as narrower and basally more sharply defined hair bands on the apical margins). *Amegilla suzanneae* sp. nov. also generally have a less bright, fulvous to slightly orange, pilosity while *A. feronia* have a brighter, bright orange, pilosity.

*Amegilla suzanneae* sp. nov. can be distinguished from *A. sumatrana* and *A. jacobi* by the wider and more colourful (fulvous) hair bands, the tergal discs being less covered by black pilosity than in these species. *Amegilla suzanneae* sp. nov. can be distinguished from the trio *A. floresiana* sp. nov., *A. gigas* and *A. vigilans* dark form by being a smaller species of ~ 16 mm (while the trio is ~17–18 mm) and having a clypeus less protuberant, in profile view smaller than the width of the compound eye (while the trio have a clypeus more protuberant that, in profile view, equals or exceeds the width of the compound eye). *Amegilla suzanneae* sp. nov. differs from *A. cyrtandrae* by the ivory-yellow to orange pale marks that are slightly less extended (while *A. cyrtandrae* have ivory-white to pale ivory-yellow clypeal marks that cover a slightly larger surface of the clypeus), a scape usually ivory-yellow to yellow anteriorly but that can also be dark brown (while *A. cyrtandrae* have a dark/black scape anteriorly) and a dorsal pilosity of the mesosoma bright (bright fulvous to bright orange) with fewer dark hairs intermixed (while *A. cyrtandrae* have a dorsal pilosity of the mesosoma pale with many black hairs intermixed).

##### Description.

**Male**: Unknown.

**Female**: Length of 16–17 mm. Interalar width of ~3.9–4 mm (shorter distance between the base of the tegulae).

***Head***: Labrum rectangular, wider than long (17:20–19:20, 1.7 × 2 mm – 1.9 × 2 mm, thus sometimes hardly visible), with two not really protruding brownish to almost black areas at the base, remaining labrum variable from entirely ivory-yellow to orange except for the black transverse carina at the apex of the labrum and sometimes a dark brown to black band that can vary in width just before the labral carina. Labrum entirely, evenly, and densely punctate with interspaces of approximately or less than the diameter of a puncture (the colouration of the labrum makes it sometimes difficult to see). Labrum entirely covered by relatively dense ochraceous pubescence, the setae are approximately or slightly longer than the diameter of an ocellus. Mandibles bidentate but take care that the second tooth is sometimes blunt and/or partially covered by the labrum leading to an hardly visible character. Mandibles pale (ivory-yellow to orangish yellow) on the basal 1/2 and black on the apical 1/2 (sometimes only black on the apical 1/3). Clypeus mainly black to reddish brown with dark clypeal marks compound of two large sub-rectangular areas, the pale clypeal marks on the clypeus are resumed to an ivory-yellow to yellow-orange inverted T-shape that is more extended on the area between the paraocular area and the labrum. Clypeus entirely, finely, and relatively densely punctate with interspace of at least the diameter of a puncture. Clypeal carina only slightly protruding, rather blunt (but not as much as in *A. gigas*) and impunctate (vertical bar of the pale T). Clypeus entirely, evenly, and relatively densely covered by an ochraceous pubescence with some black hairs intermixed (length of the hairs variable). Paraocular areas not protruding and pale (ivory-yellow to yellow) only on the apical 1/2, near the clypeus. Paraocular areas evenly, entirely, and densely punctate with interspaces of ~1/2 the diameter of a puncture (sometimes hardly visible through the pubescence). Clypeus not strongly protuberant, in lateral view protuberance of the apical margin of the clypeus less than the diameter of the eye. Scape pale on the anterior surface, sometimes with an ivory-yellow to yellow area surrounded by a dark brown integument or only a dark brown integument but never black and posteriorly dark with a black integument. Scape anteriorly with a carina around the centre of the anterior surface. Scape entirely covered by fulvous pubescence of at most the width of the scape, the pilosity is denser while going towards the sides of the scape but less dense on the anterior surface. A2 entirely black or sometimes at least partly brownish reddish anteriorly. Anterior surface of A3 basally black and paler (brown) on the apical 1/3, sometime presence of a very narrow ivory-white line at the apex of the article, posterior surface black. A4-12 brown-orange on anterior surface and posterior surface black. A3-12 entirely and evenly covered by very small, hardly visible, white pilosity. A2 anteriorly and apically with a small tuft of very small (but larger than the pilosity on the other articles) ochraceous to fulvous hairs. A3 longer than wide, equal to slightly longer than 4+5+6. A4-5 shorter than wide. A6-11 squared, approximately as long as wide. A12 not cylindrical, obliquely truncated at the apex. Frons to gena black (except an ivory-yellow to yellow-orange triangle or inverted T at the margin of the frons) and rather evenly covered by an ochraceous to fulvous pubescence of various length that is denser near the antennal insertions. Some long black hairs intermixed in the pale pubescence near the ocelli. Presence of a relatively shallow depression that goes from the middle ocellus to the pale mark of the frons, width of approximately the diameter of a surrounding puncture, the carina hardly showing through the pubescence. Genae with very long white to slightly yellow hairs.

***Mesosoma***: Mesosoma entirely covered by fulvous-orange pubescence, with some to many black hairs intermixed in the pilosity of the dorsum. Scutum and scutellum entirely, finely, and closely punctate with interspaces generally ≤ 1/2 the diameter of a puncture. Presence of a depression, the width less than the diameter of a surrounding puncture, on the centre of the scutum that does not go to the end of the scutum. Cuticle of the mesosoma deep black under the pilosity. Tegulae hyaline ochraceous to hyaline deep brown, sparsely and shallowly punctate with interspaces of at least one diameter of a puncture. Tegulae sparsely and entirely covered by ochraceous or black pubescence. Wings sparsely covered by dark hairs on the veins and inside the cells with a denser pilosity on the veins (the apical part of the cells is covered by hairs generally). Apex of the wings covered with very sparse and dark brown protuberances that are not similar to the hairs. Wings translucent but slightly yellow. Colours of the cuticle of the legs variable from brown to dark brown, some parts sometimes even black. All legs exteriorly covered by bright orange-fulvous pubescence and interiorly covered with a slightly darker to black pubescence. Tarsal claws of all the tarsi bidentate with the second tooth situated on the middle of the main claw. Tarsal claws very sharp, the secondary fore-claw is very long, 1/3 less than the main one while the other secondary claws are only 1/3 of the main one. Main claws arched, with a small tubercle on the ventral surface just above the secondary claw. Apex of mid tibia with one long and thick black spine which is crenulate on one both sides; the spine is curved at the apex. Apex of hind tibia with two long and thick black spines which are crenulate on both sides; spines curved at the apex.

***Metasoma***: T1 entirely covered by short ochraceous to fulvous pubescence except on the sides and on the base of the discs where there are longer hairs of the same colour. T2 almost entirely covered by short ochraceous to fulvous pubescence with at least a small zone of black hairs at the basal centre of the disc (that can be almost all the width of the tergum sometimes), leading to more or less hair bands at the apex of the tergum. T3-4 with wide ochraceous to fulvous short pubescence, pale pubescence a little bit more expanded on the sides of these terga, disc of the terga generally mainly black haired (with some pale hairs intermixed) except sometimes on the T4 where the centre of the tergum is widely covered by the pale pubescence (with some black hairs intermixed). T5 generally black haired, sometimes with ochraceous pubescence widely intermixed. T1-5 entirely, evenly, and sparsely punctate with shallow punctation and interspaces of at least the diameter of one puncture. Integument of the terga mainly black but the apical margin sometimes slightly paler or ochraceous on T1-4 it is therefore highly probable that the apical margins were originally deep brown to ochraceous in fresh specimen (similarly to *A. pendleburyi* and *A. feronia*) but the integument decoloured with the time. All sterna with hair bands on the apical margin, pilosity ochraceous on S1-5, black on S6 and brownish and very dense on S7. Sides of S3-5 with ochraceous hair tufts. Integument of S1-2 ochraceous-yellow, S3-5 brown, basal 1/2 of S6 brown, apical 1/2 and S7 black. All the sterna can also be deep brown to black with apical margin pale (ochraceous) or not. S1-2 mainly impunctate with only some small areas with very close and shallow punctation on the centre of the sterna. Sides and apex of S3-4 densely and evenly punctuate with interspaces of approximately the diameter of a puncture (but sometimes more and sometimes less), basal 1/2 of these sterna very sparsely punctuate with interspaces generally several times the diameter of a puncture. S5 densely punctuate but with some interspaces larger than the diameter of a puncture. S6-7 more densely, entirely, and evenly punctuate with interspaces of at most the diameter of a puncture.

##### Remarks.

This description is based on three specimens, workers should therefore consider that intraspecific variation could occur for this species, especially for some characteristics like the clypeal marks and the colouration, or even the morphology of the mandibles. These three specimens show some variations (especially in colouration, which mainly differs for the two first antennal articles and slightly differs for the pale mark of the clypeus) but also very slight differences in punctation (mainly on the clypeus, slight changes in term of density of punctation).

The male of this species is currently unknown but this species is, based on the currently recorded specimens, restricted to northern Borneo. As *A. suzanneae* sp. nov. is closely related to *A. pendleburyi*, workers should be careful concerning the identification of this group of closely related species in this region.

*Amegilla suzanneae* sp. nov. is currently considered to be closely related to *A. celineae* sp. nov. and *A. pendleburyi* due to the morphological and biogeographical proximity (Fig. 26). New collection and genetic studies should be performed in order to characterise the relationship between these three species and maybe also with *A. feronia* which is also morphologically closely related to *A. pendleburyi*. Moreover, new expeditions would allow us to gain a better understanding of *A. suzanneae* sp. nov., either biogeographically or ecologically as little is known due to the small number of specimens (only the three type specimens) currently available.

##### Etymology.

Based on the name of FC’s goddaughter, Suzanne.

##### Distribution.

*Amegilla suzanneae* sp. nov. is distributed in the Malaysian part of Borneo (northern Borneo), in the regions of Sabah and Sarawak.

#### ﻿ Amegilla (Glossamegilla) celineae

Taxon classificationAnimaliaHymenopteraApidae

Carion sp. nov.

389A45B1-43DF-5EFE-9439-B847CDE30A6E

https://zoobank.org/B36CA2C7-75F7-4E70-91C9-258B1A8C4870

Fig. 8

##### Type material.

***Holotype*: Indonesia** • ♀; S. Borneo [East Kalimantan], Gn. Pandjang, Tanggarang [Gunung Panjang]; 4 Jul. 1937; Mrs. M.E. Walsh leg.; RMNH, RMNH.INS.1713956.

##### Diagnosis.

Male: Unknown.

Female: *Amegilla celineae* sp. nov. is a species without typically contrasting hair bands on T2-4, except on T3-4 where the hair bands are slightly more contrasting (but not as much as in the banded species). *Amegilla celineae* sp. nov. can be differentiated from both colour forms of *A. amymone* by the pale clypeal mark more extensive, not restricted to a small triangular paler mark at the apex (while *A. amymone* have pale clypeal marks restricted to a small triangular paler mark at the apex, sometime with a narrow line of the same colour just above) and terga without apricot-orange pubescence (while *A. amymone* have apricot-orange pubescence on at least T4-6, sometimes only apically on T4). *Amegilla celineae* sp. nov. differs from *A. himalajensis* (Radoszkowski, 1882) by the pale yellow clypeal marks (while the marks are dark brown in *A. himalajensis*) as well as the presence of black hairs intermixed in the pubescence of the mesosoma (while *A. himalajensis* does not have any black hairs intermixed on the mesosoma). *Amegilla celineae* sp. nov. can be separated from the trio *A. insularis*, *A. pagdeni* Lieftinck, 1956 and *A. cinnyris* by a more protuberant clypeus, in profile view the clypeus equals or slightly exceeds the width of the compound eye (while the trio have a less protuberant clypeus that in profile view is smaller than the width of the compound eye, sometimes only a bit smaller) and the outside of the basitarsus III without black hairs, entirely covered by pale pubescence (while the trio have a basitarsus III at least partially covered by black pubescence). *Amegilla celineae* sp. nov. differs from *A. vigilans* by less extended yellow clypeal marks (while *A. vigilans* have more extended and momre orangish pale clypeal marks), a brighter pilosity (more bright fulvous to bright orange while *A. vigilans* is more ochraceous to slightly fulvous), terga entirely and more evenly clothed by fulvous to orange pubescence leading to not very contrasting hair bands at the apex of the terga, except on T3-4 where the hair bands are more visible (while *A. vigilans* have the terga entirely clothed with pale ochraceous pubescence that is slightly denser apically, giving slightly contrasting and poorly-defined hair bands, the hair bands on T3-4 do not contrast more than these on the other terga, hair bands more consistent) as well as hind tibiae orange to pale brown (while *A. vigilans* have usually dark hind tibiae, neither orange nor pale brown).

*Amegilla celineae* sp. nov. is therefore morphologically close to *A. pendleburyi* but differs by the mainly impunctate paraocular areas (only a very punctures visible while *A. pendleburyi* have entirely punctate paraocular areas), the labrum not entirely and evenly punctate with the presence of two impunctate areas on the sides of the labrum (below the two protuberances) (*A. pendleburyi* have a labrum entirely and evenly punctate), punctation of the clypeus coarser with smaller and hardly visible interspaces (while *A. pendleburyi* have a clypeus with finer punctation and broader well-visible interspaces), a metasoma with a more uneven pubescence and hair bands at the apex of the terga more contrasting (T3-4 basally covered by a small black haired area) (*A. pendleburyi* have a more even pubescence, the terga usually do not have any apical contrasting hair bands) as well as a larger size of ~19 mm (while *A. pendleburyi* is smaller, ~18 mm maximum).

##### Description.

**Male**: Unknown

**Female**: Length of 19 mm. Interalar width of ~3.8 mm (shorter distance between the base of the tegulae).

***Head***: Labrum rectangular, wider than long (8:7, 2.4 × 2.1 mm), with two slightly protruding brownish areas at the base, remaining labrum entirely brownish yellow except for the brown transverse carina at the apex of the labrum. Centre of the labrum punctate with a dense and deep punctation (interspaces of at most the diameter of a punctures but generally smaller). Sides of the labrum less punctate with impunctate areas just below the protuberances of the labrum as wells as a less dense punctation on remaining sides with interspaces of at least the diameter of a puncture (generally larger than this diameter). A band on the centre of the labrum covered by long fulvous setae of approximately the size of the diameter of an ocellus, apex of labrum also covered by a band of (denser) hairs that are of the same colour but smaller. Mandibles not bidentate (bi-dentation not visible) and very blunt, almost square, basal 1/2 darkened yellow, other 1/2 dark brown, and the apex black. Clypeus mainly reddish brown with dark clypeal marks compound of two large sub-rectangular areas, pale clypeal marks on the clypeus resumed to a yellow-orangish inverted T-shape that is a more extended on the area between the paraocular area and the labrum. Clypeus densely and entirely punctate with a coarse and deep punctation (interspaces of ≤ 1/2 the diameter of a puncture but generally smaller). Clypeal carina protruding, slightly blunt but not very blunt and impunctate (vertical bar of the yellow T). Clypeus entirely, evenly but sparsely covered by fulvous pubescence with the hairs slightly longer than the diameter of an ocellus. Paraocular areas protruding and yellow, mainly impunctate except at the base, near the insertions of the antennae (punctation similar to the clypeus). Protuberance of the apical margin of the clypeus of approximately the diameter of the eye (or slightly less). Scape anteriorly dark orange or slightly reddish, posteriorly dark brown to black and covered by fulvous to slightly orange pubescence that is longer than the width of the scape. A2 dark brown to slightly reddish. Anterior surface of A3 mainly orangish brown, basally with a paler orange area and apically with a narrow ivory-white line; posterior surface dark brown to black. A4-5 basally dark brown and apically orange, ending in a narrow ivory-white line on the anterior surface, posterior surface dark brown to black. A6-12 brown-orange on anterior surface and posterior surface dark brown to black. A3-12 entirely covered by very small, hardly visible, white pilosity. A2 anteriorly and apically with a small tuft of very small (but larger than the pilosity on the other articles) fulvous hairs. A3 longer than wide, slightly longer than 4+5+6. A4-7 shorter than wide. A8-11 squared, as long as wide or very slightly shorter. A12 not cylindrical, obliquely truncated at the apex. Frons to gena black (except a yellow triangle at the margin of the frons) and not evenly covered by fulvous pubescence of various length that is denser near the apical insertions. Presence of some long black hairs near the ocellus. Genae with very long white to slightly yellow hairs.

***Mesosoma***: Mesosoma entirely covered by bright fulvous-orange pubescence, without any black hairs intermixed in the pilosity of the dorsum. Scutum entirely, finely, and closely punctate with interspaces generally ≤ 1/2 the diameter of a puncture. Presence of a depression, the width less than the diameter of a surrounding puncture, on the centre of the scutum that does not go to the end of the scutum. Presence of two small but deep depression on the scutum, on each side, near the tegulae, symmetrical. Scutellum finely and densely punctate with interspaces of generally ≤ 1/2the diameter of a puncture. Presence of a small sharp carina at the centre of the scutellum that does not go on all the length of the scutellum. Cuticle of the mesosoma deep black under the pilosity. Tegulae hyaline brown-fulvous, sparsely and shallowly punctate with interspaces of twice (sometimes more) the diameter of a puncture. Wings sparsely covered by dark hairs on the veins and inside the cells with a denser pilosity on the veins. Apex of the wings covered with very sparse and dark brown protuberances that are not similar to the hairs. Wings translucent but slightly yellow. All legs with an brownish orange cuticle, except for the hind tibiae that are exteriorly a bit darker and the basitarsi that are deep brown. All legs exteriorly covered by bright orange-fulvous pubescence and interiorly covered with a slightly darker pubescence. Tarsal claws of all the basitarsi bidentate with the second tooth situated on the middle of the main claw. Apex of mid tibia with one long and thick almost hyaline brown spine which is crenulate on one side and smooth on the other side; the spine is curved at the apex. Apex of hind tibia with two long and thick almost hyaline brown spines which are crenulate on one side and smooth on the other side; spines curved at the apex.

***Metasoma***: T1 entirely and evenly covered by short fulvous pubescence except on the side where there are longer hairs of the same colour. T2 almost entirely covered by short fulvous pubescence, except a little patch of short black hairs on the centre. Discs of T3-4 at least partly covered by short black pubescence, the pale pubescence on the apical part forms hair bands that are wider at the centre of the terga. Some fulvous hairs are intermixed with the black hairs of T3-4, the hairs become longer when going towards the sides of T4. T5 mainly black-haired with short black hairs, laterally with two fulvous hair tufts composed of sparse hairs. T1-5 entirely, evenly, and sparsely punctate with shallow punctation and interspaces several times the diameter of one puncture. Integument of the terga mainly black but some parts of some apical margins are brown to ochraceous it is therefore highly probable that the apical margins were originally deep brown to ochraceous in fresh specimen (similarly to *A. pendleburyi* and *A. feronia*) but the integument decoloured with the time. All sterna mainly glabrous, hairs limited to sparse band at the apex of some sterna (2,4–6). Integument of all the sterna dark brown to orangish but not dark. S1-3 with only a few sparse punctures on the discs, the apical margins and sometimes the sides more densely punctate but with interspaces of approximately the diameter of a puncture. S4 more densely punctate at the apex and less densely at the base, interspaces become wider towards the base with only a few sparse punctures. S5 densely punctate at the apex and on the sides but interspaces become wider towards the base with only a few sparse punctures. S6-7 entirely, evenly, and densely punctate with interspaces of at most the diameter of a puncture (generally less).

##### Remarks.

This description is based on only one specimen, determinators should therefore consider that intraspecific variation could occur for this species, especially on some characteristics like the clypeal marks, the colourations, or even the morphology of the mandibles. The holotype seems to have lost pilosity on the mesosoma: the description about the pilosity of the mesosoma may therefore not be representative of the species. Consider the colour of the cuticle of the apical margin of the terga in fresh and collection specimens as decolouration can occur in collection specimens.

The male of this species is currently unknown but this species seems, based on our available specimen, to be distributed in South Borneo. However, as only one specimen is known, the distribution of this species on the island of Borneo cannot be known precisely. As *A. celineae* sp. nov. is morphologically close to *A. pendleburyi*, determinators should be careful regarding the identification of this group of closely related species in this region.

*Amegilla celineae* sp. nov. is currently considered as closely related to *A. suzanneae* sp. nov. and *A. pendleburyi* due to the morphological and biogeographical proximity (Fig. 26). New collection and genetic studies should be performed to better characterise the relationship between these three species and maybe also with *A. feronia* which is also morphologically closely related to *A. pendleburyi*. Moreover, new collections will permit to have a better understanding of *A. celineae* sp. nov., either biogeographically or ecologically as little is known due to a small number of specimens (only the holotype) currently available.

##### Etymology.

Based on the name of FC’s partner in life, Céline, who provides much support.

##### Distribution.

*Amegilla celineae* sp. nov. seems, based on our available specimen, distributed only on the island of Borneo: the collector of the holotype wrote “S. Borneo” on the label, but the name of the location provided leads to eastern Borneo, in the Indonesian part of the island. “S. Borneo” was therefore interpreted as being the Indonesian part of Borneo as a whole.

#### ﻿ Amegilla (Glossamegilla) vigilans

Taxon classificationAnimaliaHymenopteraApidae

(Smith, 1860)

45946ABF-5D01-5608-9766-6E526C14A1B4

Fig. 9

##### Material examined.

**Indonesia** • 1♂; South East Sulawesi, nr Sanggona, 1 km W of Base Camp Gn Watuwila [Sanggona]; 200 m a.s.l.; 12–15 Oct. 1989; C.v. Achterberg leg.; Malaise trap; RMNH, RMNH.INS.1713942 − 1♂; [labels unreadable]; RMNH, RMNH.INS.1689433.

##### Diagnosis.

**Male**: The male of *A. vigilans* usually do not have very contrasting hair bands at the apex of the terga, the tergal discs are entirely covered by pale ochraceous pubescence intermixed with sparse black hairs, the pale pubescence a little bit more dense at the apex of the terga but not forming a very contrasting hair bands (integument normally ochraceous at the apex, accentuating the pubescence, giving an impression of hair bands). *Amegilla vigilans* can be separated from *A. himalajensis* by the deep black dark clypeal marks that are sharply defined compared to the paler ivory-yellow to slightly salmon pale clypeal marks (while *A. himalajensis* have dark brown clypeal marks that does not contrast from each other), a dorsal pubescence of the mesosoma with black hairs intermixed (while *A. himalajensis* does not have any black hairs intermixed on the mesosoma) and all the terga entirely covered by ochraceous pubescence (while *A. himalajensis* only have T1-2 partly or wholly and sides of T3 covered by orange pubescence). *Amegilla vigilans* can be distinguished from both colour forms of *A. amymone* by the less extended pale clypeal marks that are either ivory-yellow or orange (but never ivory-white like in *A. amymone*), the two sub-rectangular dark clypeal marks (absent in *A. amymone*, dark clypeal marks different than two sub rectangular areas), an ochraceous pubescence of the mesosoma (black to dark brown with many black hairs intermixed in *A. amymone*) and an absence of apricot-orange pubescence on the terga (while *A. amymone* have apricot-orange pubescence at least on T4-6). *Amegilla vigilans* can be distinguished from the trio *A. insularis*, *A. pagdeni*, and *A. cinnyris* by a more protuberant clypeus of approximately the width of the compound eye (while the trio have a clypeus smaller than the width of the compound eye) and a basitarsus without any black pilosity, the pilosity entirely ochraceous (while the trio have at least some black hairs on the basitarsus III). Finally, *A. vigilans* can be separated from *A. pendleburyi* by the more extended, ivory-yellow to slightly salmon, pale clypeal marks (*A. pendleburyi* with clypeal mark less extended, especially the central line which is narrow, unlike in *A. vigilans* where the central mark is too thick to be labelled as a line), the pilosity that is paler, rather ochraceous to slightly yellow (while *A. pendleburyi* have a more fulvous-orange pilosity) as well as the terga entirely covered with a pale ochraceous pubescence that is denser apically, giving slightly contrasting and poorly defined hair bands (*A. pendleburyi* have terga entirely and more evenly covered by fulvous-orange pilosity, leading to the absence of contrasting hair bands).

The dark form of *A. vigilans* is a banded form of *Amegilla* close to *A. gigas* concerning the habitus but can be separated from this species by a less protuberant clypeus of, in profile view, approximately the width the compound eye (while *A. gigas* have, in profile view, a clypeus that exceeds the width of the compound eye), pale orangish clypeal mark that are less extended leading to two sub-rectangular dark clypeal marks (while *A. gigas* have yellow, very expanded, pale clypeal marks and dark clypeal marks restricted to two small black to slightly reddish marks and two thin brown lines), a pilosity less bright in general (more ochraceous-yellow for *A. vigilans* while the pilosity is more fulvous to slightly orange in *A. gigas*) as well as a dorsal pubescence of the dorsum of the mesosoma with more black hairs intermixed (fewer black hairs intermixed in the pilosity of the dorsum of the mesosoma in *A. gigas*).

##### Description.

**Male**: Length of 17.5–18.5 mm. Interalar width of ~3.3–3.8 mm (shorter distance between the base of the tegulae).

***Head***: Labrum rectangular, slightly wider than long (18:17–21:20, 1.8 × 1.7 mm – 2.1 × 2 mm, thus sometimes hardly visible, can be considered squared without measurements) with a protruding base (with two brown or yellow areas, one on each side of the base) giving a curved appearance to the labrum in lateral view. Remaining labrum yellow or brownish orange (dark form), except the black transverse carina at the apex of the labrum and a narrow deep brown band at the base, brown band at the base absent in the dark form. Labrum entirely, evenly, and densely punctate with interspaces of the size of a puncture or less. Labrum entirely covered by a not very dense and ochraceous pubescence that is equal or shorter than the diameter of an ocellus. Mandibles bidentate and blunt, giving teeth a rounded apex, yellow on the basal 2/3 and deep brown to black on the apical 1/3 (darkened yellow to brown on the base for the dark form). Clypeus mainly yellow or orange with two subrectangular dark marks that can be black to slightly reddish. Clypeus entirely, finely (not very coarsely but can be coarse sometimes) and densely punctate with interspace of at most the diameter of a puncture but generally less. Clypeal carina slightly protruding, not reduced, punctate and rather blunt. Clypeus entirely, evenly, and sparsely covered by ochraceous hairs of various length with some long darker hairs intermixed. Paraocular areas protruding, yellow or orange. Paraocular areas evenly and densely punctate with interspaces ~1/2 the diameter of a puncture (sometimes hardly visible due to the pale colouration of the integument). Clypeus not strongly protuberant, in lateral view, apical margin of the clypeus of approximately the size of the diameter of the eye. Scape anteriorly yellow or darkened orange and posteriorly black. Scape entirely covered by ochraceous pubescence of various length that is denser on the sides and less dense anteriorly and posteriorly. A2 black on the basal 1/2 and slightly orange on the apical 1/2. A3 black on the basal 2/3 and orange on the apical 1/3 anteriorly, posteriorly black. A4-13 paler (orange to brown) anteriorly and black posteriorly. A3-13 entirely and evenly covered by very small, hardly visible, white pilosity. A2 sparsely covered by ochraceous or black hair tuft that is longer than the pubescence of the next articles. A3 longer than wide, longer than 4+5 but shorter than 4+5+6. A4 shorter than wide. A5-10 square, as long as wide. A11-12 rectangular, slightly longer than wide (can be considered squared without measurements). A13 not cylindrical, obliquely truncated at the apex. Frons to gena black (except a yellow or orange triangle at the margin of the frons) and rather not evenly covered by ochraceous pubescence of various length that is denser near the antennal insertions. Long black hairs intermixed in the pale pubescence near the ocelli. Presence of a relatively deep depression that goes from the middle ocellus to the pale mark of the frons, width variable. The depression hardly showing through the pubescence. Genae with very long white to ochraceous hairs (especially long under the eyes).

***Mesosoma***: Mesosoma entirely covered by ochraceous and dense pubescence with a variable number of black hairs intermixed (but always with at least some black hairs present) in the pilosity of the dorsum. Scutum and scutellum finely and closely punctate with interspaces of generally at most the diameter of a punctures (but can be wider on some areas of scutum and scutellum). Cuticle of the mesosoma deep black under the pilosity. Tegulae hyaline deep brown, sparsely and shallowly punctate with interspaces of at least one diameter of a puncture. Tegulae relatively densely and entirely or partially covered by ochraceous pubescence. Wings relatively sparsely covered by dark hairs on the veins and inside the cells with a denser pilosity on the veins (most parts of the cells covered by pubescence). Apex of the wings covered with very sparse and dark brown protuberances that are not similar to the hairs. Wings translucent but slightly yellow. Cuticle of all the legs deep brown to dark brown, never pale. All legs exteriorly covered by ochraceous to slightly fulvous pubescence and interiorly covered with a slightly darker to black pubescence, hind basitarsi without black hairs exteriorly. Tarsal claws of all the basitarsi bidentate with the base of the second tooth situated on the middle of the main claw. Tarsal claws very sharp, the secondary claws are very long, 2/3 the length of the main one. The main claws arched but without any tubercles. Apex of mid tibia with one long and thick deep brown (dark form) to black (pale form) spine which is crenulate on both sides; the spine is slightly curved at the apex. Apex of hind tibia with two long and thick deep brown (dark form) to black (pale form) spines which are crenulate on both sides; spines slightly curved at the apex.

***Metasoma***: For the pale form, T1-6 entirely covered by short ochraceous pubescence that is denser on the apical margin for T1-5, leading to slightly contrasting hair bands on these terga. Absence of dark hairs on discs of T1-6 for the pale form. For the dark form, disc of T1 basally and centrally covered by short black pubescence, laterally and apically covered by ochraceous short pubescence that is denser apically creating a wide but poorly contrasted hair band. T2-5 covered by black pubescence on the disc and covered by ochraceous pubescence on the apical margin, leading to a strongly contrasted hair band. T6 entirely covered by brown pubescence. T1-6 entirely, evenly, and sparsely punctate with shallow punctation and interspaces of at least the diameter of one puncture. T7 (pygidial plate) with two spine-like structures. Integument of T1-6 deep brown to dark brown, sometimes the apical margins is paler (brown to ochraceous) it is therefore probable that the apical margins were originally deep brown to ochraceous in fresh specimen (similarly to *A. pendleburyi* and *A. feronia*) but the integument decoloured with the time. All the sterna entirely covered by short and relatively dense ochraceous pilosity (sometimes the S1-3 not entirely covered). Integument of sterna brown to deep brown with ochraceous apical margin (except sometimes on S1-2). S1-2 mainly impunctate with only some small areas with very close and shallow punctation on the centre of the sterna. Sides and apex of S3-4 densely and evenly punctuate with interspaces of approximately the diameter of a puncture (but sometimes more and sometimes less), basal 1/2 of these sterna very sparsely punctuate with interspaces generally several times the diameter of a puncture. S5-7 entirely, densely, and shallowly punctate with interspaces of approximately the diameter of a puncture. S8 with some very small yellow hairs on the base, spine of the spiculum rounded and not curved (Fig. 9H). Gonocoxae mainly glabrous with only a few sparse white hairs. Intern face of the apex of gonocoxae curved. Genitalia yellow to orange, not very dark (Fig. 9G).

##### Remarks.

This description is based on only two specimens; determinators should therefore consider that intraspecific variation could occur for this species, especially on some characteristics like the clypeal marks, the colourations, or even the morphology of the mandibles. However, two forms exist inside the species *A. vigilans*, namely the dark and the pale forms. These two forms do not show significant structural differences but differs on colourations and the hair bands of the metasoma (Fig. 10). Moreover, the genitalia of both forms are the same (Fig. 10G, H) and both forms are therefore considered as conspecific. The colour variation observed in this species fits a broad pattern of variation in pubescence seen across other bees on Sulawesi, such as *Xylocopa* Latreille, 1802 (van der Vecht 1953), though this would benefit from focused study.

A few specimens of this species (female of both forms and male of the dark form), hosted in the RMNH collection, were determined by Lieftinck in 1955 and 1956. It is therefore surprising that Lieftinck (1956) did not consider *A. vigilans*, even if the species was already known at the time; the reasons for the absence of this species in his work remain unknown.

##### Distribution.

*Amegilla vigilans* still appears to be restricted to the island of Sulawesi. However, the specimens studied in this paper extend the distribution of the species to the north of the island while the type was collected in the south.

#### ﻿ Amegilla (Glossamegilla) gigas

Taxon classificationAnimaliaHymenopteraApidae

Friese, 1922

9D24BD9C-DE65-5479-9F42-29AC033AE161

Fig. 11

##### Type material.

***Lectotype*: Asia Arch. [Indonesia**] • 1♀; Wetter [Pulau Wetar]; 3 Apr. 1901; Kühn leg.; ZMHB, http://coll.mfn-berlin.de/u/837c94. [images examined only]

##### Additional material examined.

**Timor [Timor-Leste**] • 1♂; Same; 27 Dec. 1965–14 Jan. 1966; R.N. Ierreira leg.;RMNH.INS.1713966.

##### Remarks on the type series of Friese.

Friese (1922: 61) mentioned two females in his type series, one from Wetar (Lesser Sunda islands) and one from Sumatra (Deli, eastern Sumatra). However, these two specimens are on each side of the Wallace Line which is, with the current information on the distribution of *Glossamegilla*, doubtful. Moreover, the ZMHB seems to only host the specimen from Wetar while the specimen from Sumatra was not found (P. Rasmont, pers. comm., 2 December 2024). Therefore, without further information, the specimen from Sumatra cannot be considered as conspecific with *A. gigas*, especially because of its doubtful locality compared to everything that is currently known on the subgenus Glossamegilla. The specimen from Wetar (March 1901, leg. Kühn) conserved in the ZMHB (examined by photograph) is considered to be the lectotype following an unpublished designation by P. Rasmont in 2024. This designation decisively fixes the locus typicus as the island of Wetar.

##### Diagnosis.

Male: The male of *A. gigas* shows strongly contrasting hair bands, with only black hairs on the discs T2-4 while T1 is entirely covered by pale fulvous pubescence. *Amegilla gigas* differs from *A. jacobi* and *A. sumatrana* by the wider and more colourful (fulvous in *A. gigas*, white to slightly ochraceous in *A. jacobi* and *A. sumatrana*) hair bands at the apex of the terga as well as a slightly smaller black-haired area. *Amegilla gigas* also differs from *A. feronia* and *A. cyrtandrae* by the more strongly protuberant clypeus of, in profile view, ~1.3 × bigger than the width of the compound eye (*A. feronia* and *A. cyrtandrae* have a less protuberant clypeus that is, in profile view, smaller than the width of the compound eye) and a larger body size of ~18 mm (while *A. feronia* and *A. cyrtandrae* are ~15–17 mm maximum). *Amegilla gigas* can be distinguished from *A. floresiana* sp. nov. by the labrum as long as wide (1:1, while *A. floresiana* sp. nov. have a labrum slightly wider than long; ~17:16–19:18), a coarser punctation of the clypeus that is not very sparse with interspaces of at most the diameter of a puncture (*A. floresiana* sp. nov. have a fine punctation that is also very sparse with large interspaces of at least the diameter of a puncture) as well as the tergal hair bands narrower and more sharply defined with the black-haired area on the tergal discs larger and more consistently present (*A. floresiana* sp. nov. have wider and poorly defined tergal hair bands at the apex of the T2-4 with a small black-haired basal part the discs, sometimes some or all the terga do not have a black-haired area and are entirely covered by the pale pubescence). Finally, *A. gigas* can be distinguished from the dark form of *A. vigilans* by a more protuberant clypeus of ~1.3 × bigger than the diameter of the eyes (1:1 in *A. vigilans*), yellow clypeal marks that are very extended with only a small dark mark and one narrow brown line on each side of the clypeal carina (*A. vigilans* have pale yellow to orange less extensive pale marks and two sub-rectangular black marks), a pilosity in general more bright (rather fulvous to slightly orange in *A. gigas*, ochraceous in *A. vigilans*) as well as a dorsal pubescence of the mesosoma without or with only few black hairs intermixed (while *A. vigilans* have more black hairs intermixed in the dorsal pubescence of the mesosoma).

##### Description.

**Male**: Length of 18 mm. Interalar width of ~3.6 mm (shorter distance between the base of the tegulae).

***Head***: Labrum square, as wide as long (1:1, 2 × 2 mm), with two slightly protruding pale brownish areas at the base, remaining labrum entirely lemon-yellow except for the dark brown to black transverse carina at the apex of the labrum. Labrum entirely but sparsely punctate with interspaces wider than the diameter of a puncture, the interspaces become narrower while going towards the apex of the labrum (interspaces of ~1/2 the diameter of the punctures). Labrum entirely covered by yellow pubescence longer than the diameter of an ocellus. Mandibles bidentate and slightly blunt (not very sharp) at the apex, the bases lemon-yellow, similarly to the labrum. Face mostly lemon-yellow, only the clypeus with four dark brown to black mark (two lines and two subrectangular areas) that resume the dark clypeal marks. Clypeus entirely and evenly punctate with relatively small interspaces of approximately the diameter of a puncture. Clypeal carina slightly protruding, blunt and impunctate. Clypeus mainly covered by black and very sparse hairs, except at the apex, near the labrum where the pubescence is yellow and denser. Paraocular areas protruding and yellow, almost entirely punctate (same punctation as on the clypeus) except some wider clear interspaces on the margin of the paraocular areas and on their centres. Clypeus protuberant, protuberance of the margin of the clypeus of ~1.3 × bigger than the diameter of the eyes (while *A. vigilans* have a clypeus of approximately the size of the eye). Scape anteriorly orangish yellow and posteriorly black, covered entirely by fulvous pilosity. A2-13 black or at least very dark and covered by very small white hairs that are hardly visible. A3 longer than wide, longer than 4+5 but slightly shorter than 4+5+6. A4-12 squared, approximately as long as wide or a little bit longer than wide. A13 not cylindrical, obliquely truncated at the apex. Frons to gena black (except a yellow triangle at the margin of the frons) and covered (but not evenly) by fulvous pubescence.

***Mesosoma***: Mesosoma entirely and evenly covered by fulvous to slightly orange pubescence, without any black hairs intermixed in the pilosity of the dorsum. As the pubescence is dense, the punctation underneath can sometimes not be seen. Scutum and scutellum finely and closely punctate (deep punctures) with very small interspaces (≤1/2 the diameter of a punctures). Cuticle under the pilosity deep black. Tegulae hyaline deep brown. Wings sparsely covered by dark hairs on the veins and inside the cells with a denser pilosity on the veins. Apex of the wings covered with very spars and dark brown protuberances that are not similar to the hairs. Wings translucent but slightly yellow. All legs with a dark brown cuticle, exteriorly covered by fulvous to slightly orange pubescence and interiorly covered by dark brown to black pubescence. Tarsal claws of all the basitarsi bidentate. Apex of mid tibia with one long and thick brown spine which is crenulate on one side and smooth on the other side. Apex of hind tibia with two long and thick brown spines which are crenulate on one side and smooth on the other side.

***Metasoma***: T1 entirely covered with long fulvous pubescence that is denser and shorter on the apical margin, forming a hair band that does not contrast very much in the surrounding pilosity. Discs of T2-4 dark haired with whitish to fulvous hair bands on the apical margins. Disc of T2 laterally with fulvous hair tufts covering all the length of the disc but that does not expand very much transversally. Discs of T3-4 laterally with small, not very expanded, fulvous hair tuft. T5 and following entirely black haired. Discs of T1-5 sparsely punctate with shallow punctations and interspaces generally between 1.5 to twice the diameter of a puncture. Apical margin of T1-5 impunctate. T6-7 entirely and less sparsely punctate, punctation of T6 as shallow as previous terga but punctation of T7 deeper. T7 ending in two protruding spines at the apex with a curve between the two spines. Integument of the terga entirely black except for T6-7 that have a dark brown integument. S1-6 mainly black haired. S1-4 with tuft of not very expanded ochraceous hairs on the sides of the apical margin, S5 black haired and S6 with a band of ochraceous hairs on the apical margin. S1-3 with a deep brown integument, S4-6 with a darker brown to black integument. S1-3 very sparsely punctate of the disc with only a few punctures except on the sides where there is a denser punctation under the hairs. S4-5 more densely punctuate with an evenly shallow punctation (interspaces of approximately the diameter of one puncture). S6 densely punctuate on the sides and with only a few punctures on the centre of the disc. S7-8 with some very small yellow hairs on the base, spine of the spiculum very sharp and backwardly curved (Fig. 11H). Gonocoxae mainly glabrous with only a few sparse white hairs. Intern face of gonocoxae crenulate. Genitalia brownish at the base, tending yellow while going toward the apex (Fig. 11G).

##### Remarks.

This description is based on only one specimen; determinators should therefore consider that intraspecific variation could occur for this species, especially on some characteristics like the clypeal marks, the colourations, or even the morphology of the mandibles. Moreover, some doubts exist concerning the sex association between the male and the female. Indeed, the male is similar to the lectotype and shows a close distribution, without being identical (take care that the unmatching distribution can be a bias from the undersampling in the region). The islands of Timor and Wetar are separated by a straight of ca 50 km, although the small island of Ataúro forms a potential intermediate step, separated by 26 km from Timor (at Dili) and 13 km from Liran island, just to the south-west of Wetar. Some morphological differences exist between these two specimens. These differences consist mainly of the paraocular area being yellow (ivory-white for the female), yellow covering a greater surface of the face (clypeus almost entirely yellow, big yellow mark between the antennae while the female has an almost entirely dark clypeus and a smaller yellow triangle between the antennae), a labrum entirely yellow (female has a partially dark labrum), A3 longer than 4+5 but slightly shorter than 4+5+6 (female has the A3 as long as 4+5+6+7), and a more protuberant clypeus of 1.3 the width of the compound eye (female has a clypeus of approximately the width of the compound eye) (females characters are based on images of the lectotype and the description of Friese 1922: 61). All these small differences are currently considered as intraspecific variations and dimorphism but bring some doubts to the pairing of both sexes. New collections and genetic studies should be performed in order to give a definitive answer for this case but, while waiting for these, the specimens from Timor is considered as the male of *A. gigas*.

##### Distribution.

With the discovery of the supposed male of *A. gigas*, the distribution of this species is enlarged to the island of Timor and more specifically the country of Timor-Leste. The species was originally known from the island of Wetar (Friese 1922). As discussed for the type series, the specimen from Sumatra is not considered as conspecific; therefore, this species is not distributed in Sumatra.

#### ﻿ Amegilla (Glossamegilla) anthracina

Taxon classificationAnimaliaHymenopteraApidae

(Gribodo, 1894)

2748FD23-E2DF-5770-B3F0-432237583E39

Anthophora violacea var. anthracina Gribodo, 1894a: 388, ♀ [Indonesia: Tanimbar, MSNG].

##### Material examined.

**Indonesia** • 1♀; Tenimber [Tanimbar]; [likely 1892]; [likely W. Doherty leg.]; MSNG (lectotype designated by Wood & Bossert in press) • 1♀; Tenimber [Tanimbar]; [likely 1892]; [likely W. Doherty leg.]; MSNG (paralectotype)

##### Remarks.

This “variety”, known from only two females from the Gribodo collection (MSNG), will be formally elevated to species status by Wood and Bossert (in press) based on morphological and strong biogeographical differences with *A. violacea* (Lepeletier, 1841). Considering the subgeneric placement of *A. violacea* (Brooks 1988), the similarity of A. violacea var. anthracina with *A. violacea*, the long tongue as well as the short velvety pubescence covering the metasoma, this “variety” should logically be considered as a *Glossamegilla* and is therefore included in our work in order to increase the utility of the identification key.

##### Distribution.

Amegilla var. anthranica is currently only known from the Tanimbar islands, in Indonesia (Gribodo 1894a).

#### ﻿ Amegilla (? Glossamegilla) tetrataeniata

Taxon classificationAnimaliaHymenopteraApidae

(Gribodo, 1894)

28396CAF-D841-596A-8399-0FABE2655F86

Anthophora tetrataeniata Gribodo, 1894b: 275, ♀ [Timor, MSNG]

##### Material examined.

**West Timor/Timor-Leste** • 1♀; Timor; [likely 1892]; [likely W. Doherty leg.]; MSNG (holotype) • 1♀; Timor [no further information]; M.A. Lieftinck det. 1966; RMNH.

##### Remarks.

This species is currently (to our knowledge) only known from two females which are the holotype (MSNG) and one undated specimen (RMNH). *Amegilla tetrataeniata* was placed into the subgenus Dizonamegilla by Brooks (1988), but this choice is confusing as the male is unknown and this subgenus is largely defined based on male characters. However, considering the biogeography of this species, the long tongue, the slightly swollen yellow-marked “tubercles” on the lower paraocular areas, as well as the presence of other *Glossamegilla* species in the region (the Lesser Sunda islands), a placement in the subgenus Glossamegilla seems more likely. *Amegilla tetrataeniata* is similar to *A. anthracina* but differs by the presence of white hairs on the gena, vertex, scutellum, and T3-4. Without males and further data for this species, this placement is currently uncertain. It is included in the identification key for utility and to facilitate its further study.

##### Distribution.

*Amegilla tetrataeniata* is currently known only from the island of Timor (Gribodo 1894b; Brooks 1988).

#### ﻿ Amegilla (Glossamegilla) amymone

Taxon classificationAnimaliaHymenopteraApidae

(Bingham, 1896)

3A57333A-D59B-5A52-8664-67C5C06D3EDB

Anthophora amymone Bingham, 1896: 196, ♀ [Indonesia: Sumatra, NHMUK, examined] (Fig. 12)
Anthophora bouwmani Lieftinck, 1944: 103, ♀ [Indonesia: Sumatra, RMNH, examined] (Fig. 13) syn. nov.

##### Remarks.

In the material donated to the RMNH collection in 2008 from R. Desmier de Chenon were 24 specimens of “*A. bouwmani*”, all identified by R. Desmier de Chenon as *A. amymone*, from Sumatra that were collected at the same time and place as another ten specimens of *A. amymone* (see map, Fig. 25). Only males of *A. bouwmani* were found by R. Desmier de Chenon while he collected both sexes of *A. amymone* at the same place. The sympatry of these two taxa, the collection of only males of *A. bouwmani* but both sexes of *A. amymone* led to the hypothesis that *A. amymone* and *A. bouwmani* may be conspecific. Indeed, *A. bouwmani* was originally described from a single female specimen, with the male unknown.

The comparison of these males of *A. bouwmani* with the holotype of *A. bouwmani* hosted at the RMNH showed similarities in terms of pilosity and structure but also a difference in its clypeal mark; this level of difference is similar to the one characterising both sexes of *A. amymone*.

However, when morphology was compared between the males of the two species, no clear differences were observed, with the clearly overlapping range. Indeed, most of the specimens have shared distributions with shared altitudes (both seem to be mountain species) as visible on the map (Fig. 25). The males have the same habitus but a paler pilosity is observed in *A. bouwmani* (Fig. 14A, B, E, F), the clypeal marks are approximately the same (same colour, approximately the same surface is covered, Fig. 14C, D), the labra are very similar (Fig. 14C, D), the punctation shows no clear differences and the genitalia are the same in both species (Fig. 14G, H). Concerning the females, similarly to the males, only the colour of the pubescence changes with a paler pubescence for *A. bouwmani*. Lieftinck (1944: 103–105) gave a full description of *A. bouwmani* as the original description but *A. amymone* was not considered in this paper. Later, Lieftinck (1956: 9) described *A. bouwmani* as “The female […] resembles that of *amymone* fairly closely in texture and colour of the body, but the pubescence is much lighter.”, suggesting that these two species are closely related (Lieftinck also put them as “near allies” in his paper of 1956). This is interpreted here as an additional argument to their synonymy.

**Figure 15.**
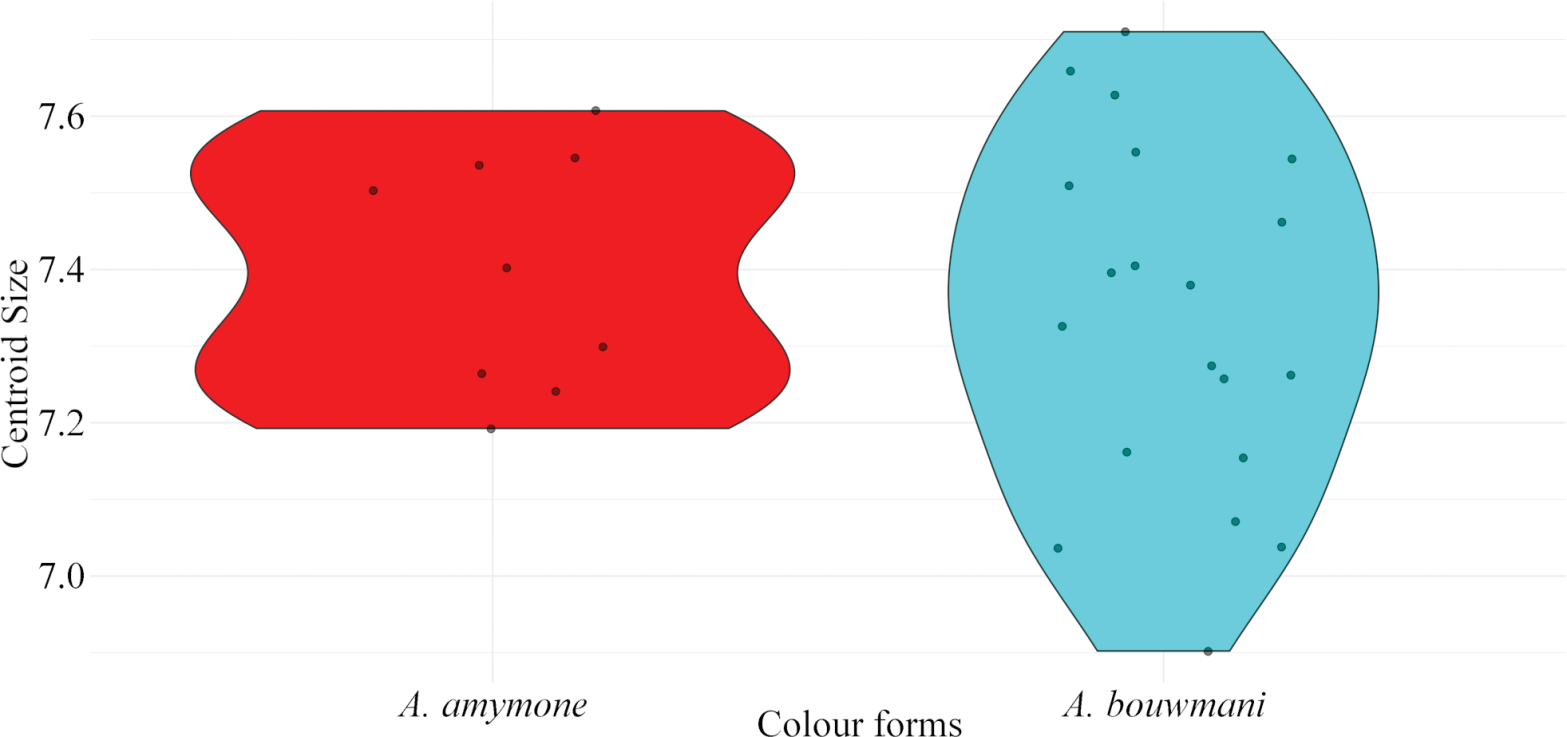
Distribution of the centroid sizes for the wings in the males of *A. bouwmani* and *A. amymone*. No significant differences.

**Figure 16.**
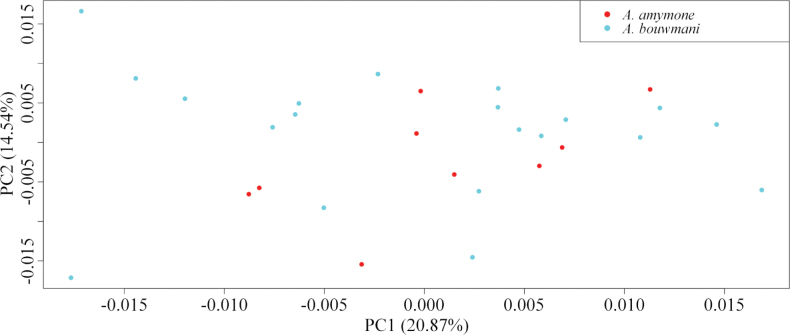
Ordination of *A. bouwmani* and *A. amymone* along the two first axes of the Principal Component Analyses (PCA) (explaining 20.87% and 14.54% of the variance, respectively).

Contrarily to most of the other species assessed, *A. amymone* and *A. bouwmani* cannot be differentiated based on wing size and shape. Concerning wing size, a substantial overlap occurs between both species when the distribution of size is examined. Wing size in *A. amymone* overlaps at 100% the size of the wing of *A. bouwmani*, the latter being more variable than the former (Fig. 15). However, differences in wing size is not statistically significant (p-value = 0.495; Table 6). Concerning wing shape, the PCA plot shows a complete overlapping between both species, without any distinct group differentiating. This absence of differentiation is confirmed by the result of the Procrustes ANOVA, the shape of the wing being indeed not significantly different (Fig. 16; p-value = 0.494; Table 7).

**Table 6.** Results of the ANOVA test for the differentiation of sizes of the centroids for the males of *A. bouwmani* and *A. amymone*. Df is the Degree of Freedom. Sum Sq is the sum of squared differences between observed data and averages. Mean Sq is the sum of squared divided by the corresponding degree of freedom. F value is the ratio of variance explained to residual variance. No significant p-value at a p < 0.05 threshold.

	Df	Sum Sq	Mean Sq	F value	P-value (>F)
Species	1	0.024	0.024	0.55	0.465
Residuals	27	1.182	0.044		

**Table 7.** Results of the Procrustes ANOVA for the males of *A. bouwmani* and *A. amymone*. Df is the Degree of Freedom. SS is the sum of squared differences between observed data and averages MS is the sum of squared divided by the corresponding degree of freedom. Rsq is the proportion of total variance explained by the statistical model. F is the ratio of variance explained to residual variance. Z is the difference between the means of the two groups being compared divided by the standard deviation of this difference. No significant p-value at a < 0.05 threshold.

	Df	SS	MS	Rsq	F	Z	P-value (>F)
Forms	1	0.000	0.000	0.034	0.943	-0.004	0.494
Residuals	27	0.011	0.000	0.966			
Total	28	0.011					

Evidence from all these arguments demonstrates that *A. amymone* and *A. bouwmani* are most likely the same species, as they only have colouration differences and no significant structural differences nor significant biogeographic differences. *Amegilla bouwmani* syn. nov. is therefore considered here as a pale form of *A. amymone*.

### ﻿Checklist of the Indo-Australian subgenus Glossamegilla

**Figure 17.**
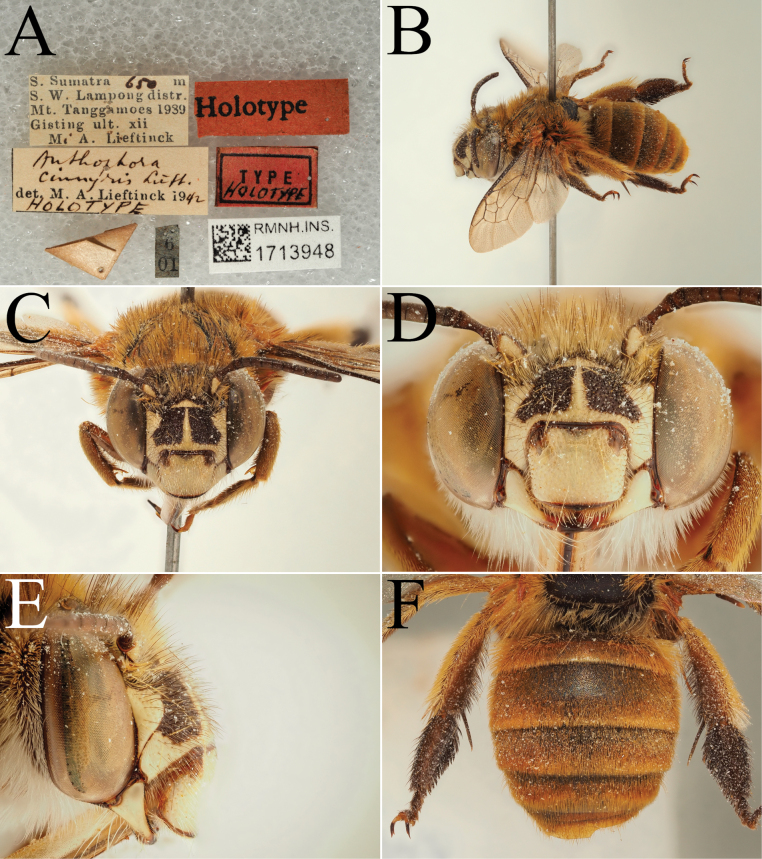
Holotype of *A. cinnyris* (Lieftinck, 1944) (RMNH). A. Labels of the specimen; B. Habitus in profile view; C. Face in frontal view; D. Labrum in ventral view; E. Protuberance of the clypeus in profile view; F. Terga in dorsal view.

**Figure 18.**
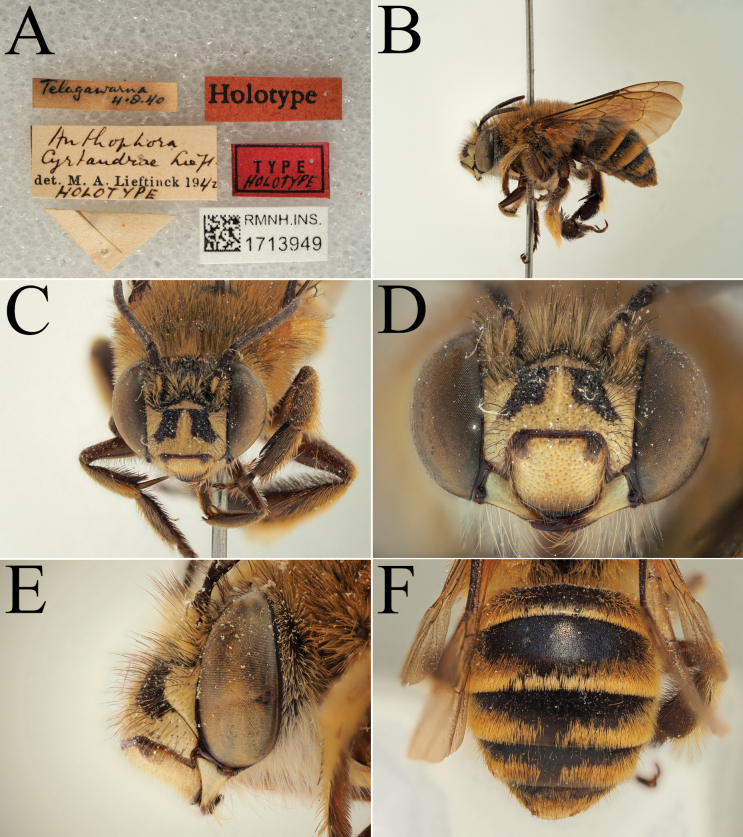
Holotype of *A. cyrtandrae* (Lieftinck, 1944) (RMNH). A. Labels of the specimen; B. Habitus in profile view; C. Face in frontal view; D. Labrum in ventral view; E. Protuberance of the clypeus in profile view; F. Terga in dorsal view.

**Figure 19.**
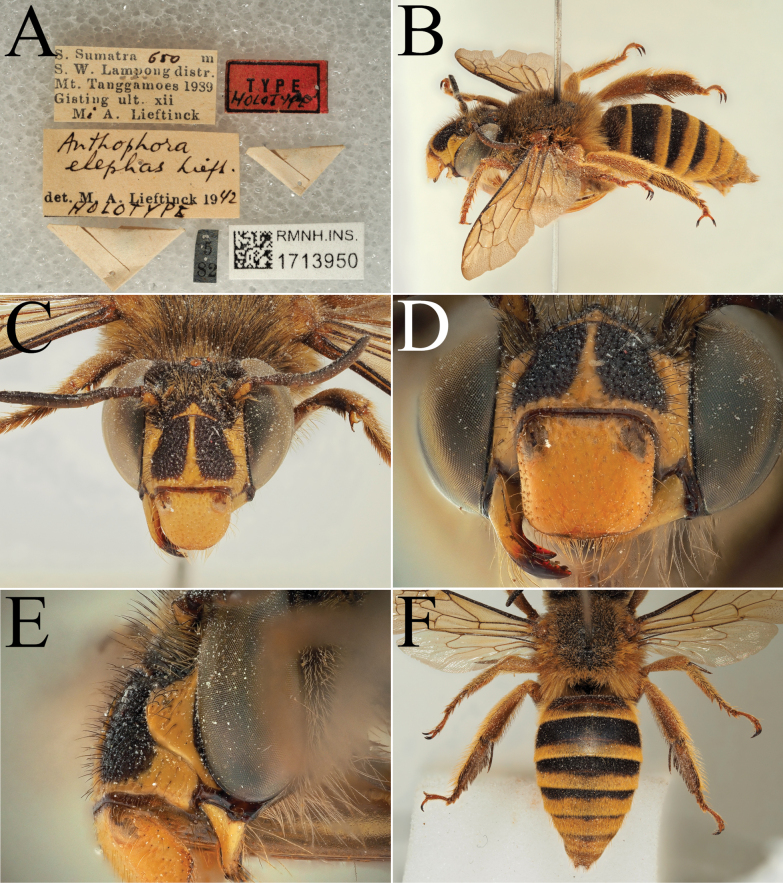
Holotype of *A. elephas* (Lieftinck, 1944) (RMNH). A. Labels of the specimen; B. Habitus in profile view; C. Face in frontal view; D. Labrum in ventral view; E. Protuberance of the clypeus in profile view; F. Terga in dorsal view.

**Figure 20.**
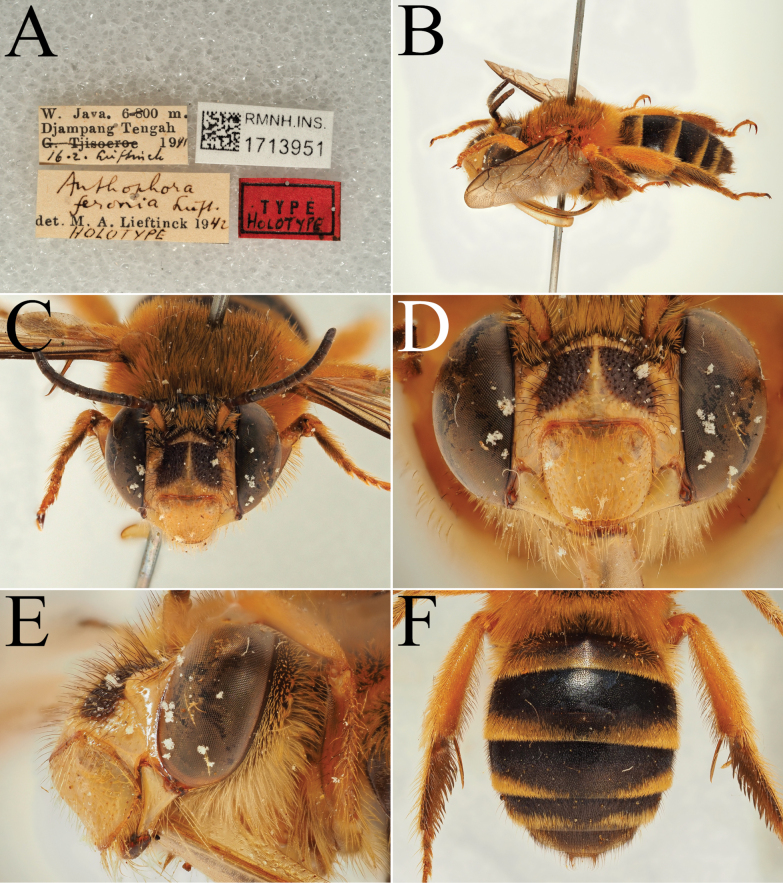
Holotype of *A. feronia* (Lieftinck, 1944) (RMNH). A. Labels of the specimen; B. Habitus in profile view; C. Face in frontal view; D. Labrum in ventral view; E. Protuberance of the clypeus in profile view; F. Terga in dorsal view.

1. Amegilla (Glossamegilla) amymone (Bingham, 1896) (= *A. bouwmani* (Lieftinck, 1944) syn. nov.)

2. Amegilla (Glossamegilla) anthracina (Gribodo, 1894)

3. Amegilla (Glossamegilla) celineae Carion, sp. nov.

4. Amegilla (Glossamegilla) cinnyris (Lieftinck, 1944) (Fig. 17)

5. Amegilla (Glossamegilla) cyrtandrae (Lieftinck, 1944) (Fig. 18)

6. Amegilla (Glossamegilla) elephas (Lieftinck, 1944) (Fig. 19)

7. Amegilla (Glossamegilla) feronia (Lieftinck, 1944) (Fig. 20)

8. Amegilla (Glossamegilla) floresiana Carion & Wood, sp. nov.

9. Amegilla (Glossamegilla) gigas (Friese, 1922)

10. Amegilla (Glossamegilla) hanitschi (Meade-Waldo, 1914)

11. Amegilla (Glossamegilla) himalajensis (Radoszkowski, 1882)

12. Amegilla (Glossamegilla) insularis (Smith, 1857)

13. Amegilla (Glossamegilla) jacobi (Lieftinck, 1944) (Fig. 21)

**Figure 21.**
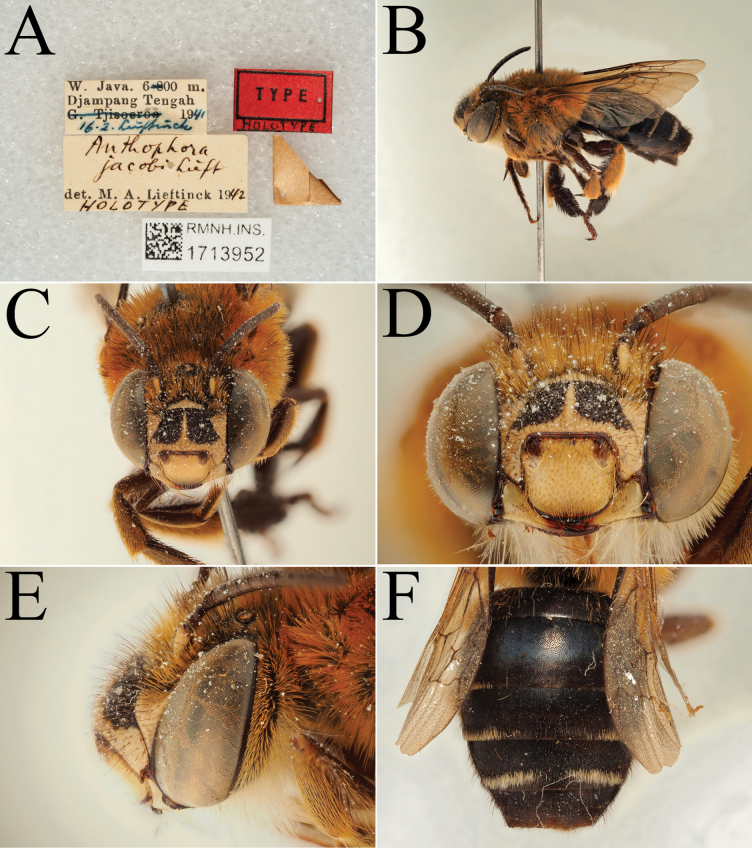
Holotype of *A. jacobi* (Lieftinck, 1944) (RMNH). A. Labels of the specimen; B. Habitus in profile view; C. Face in frontal view; D. Labrum in ventral view; E. Protuberance of the clypeus in profile view; F. Terga in dorsal view.

**Figure 22.**
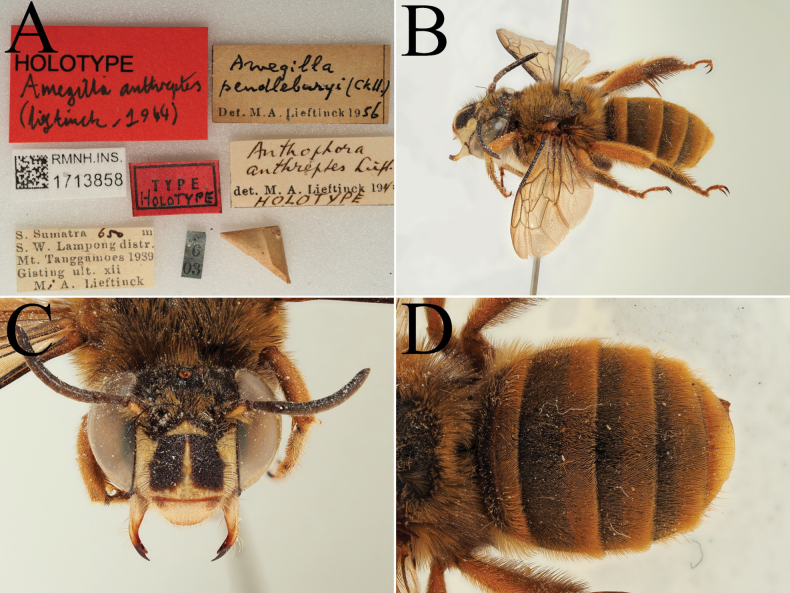
Holotype of *A. anthreptes* (Lieftinck, 1944), synonymised with *A. pendleburyi* (Cockerell, 1929) by Lieftinck (1956) (RMNH). A. Labels of the specimen; B. Habitus in profile view; C. Face in frontal view; D. Terga in dorsal view.

**Figure 23.**
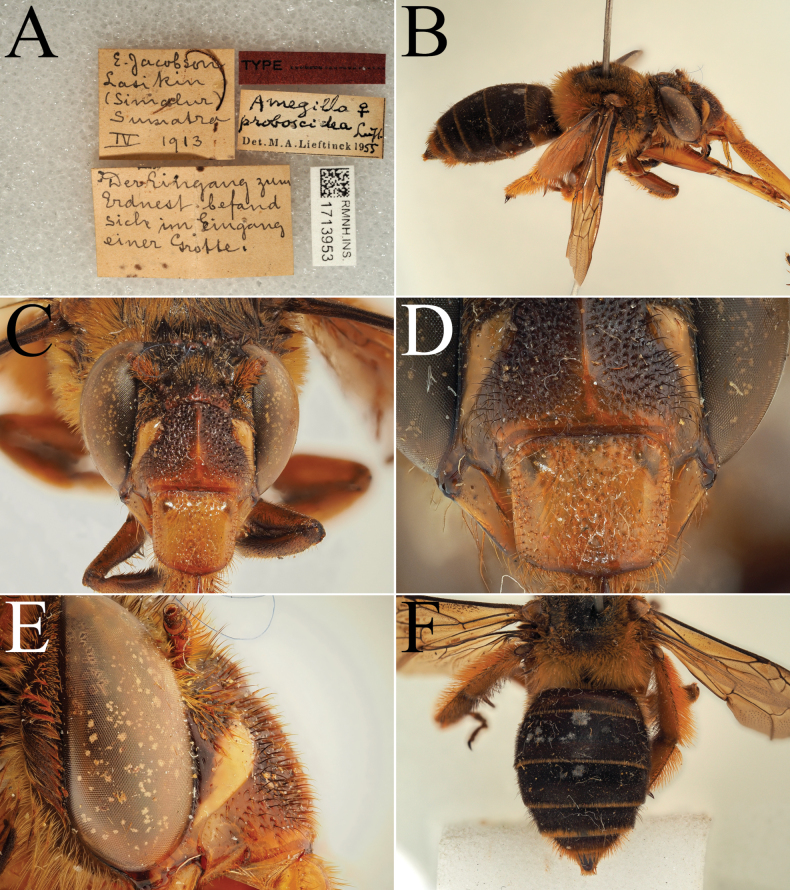
Holotype of *A. proboscidea* Lieftinck, 1956 (RMNH). A. Labels of the specimen; B. Habitus in profile view; C. Face in frontal view; D. Labrum in ventral view; E. Protuberance of the clypeus in profile view; F. Terga in dorsal view.

**Figure 24.**
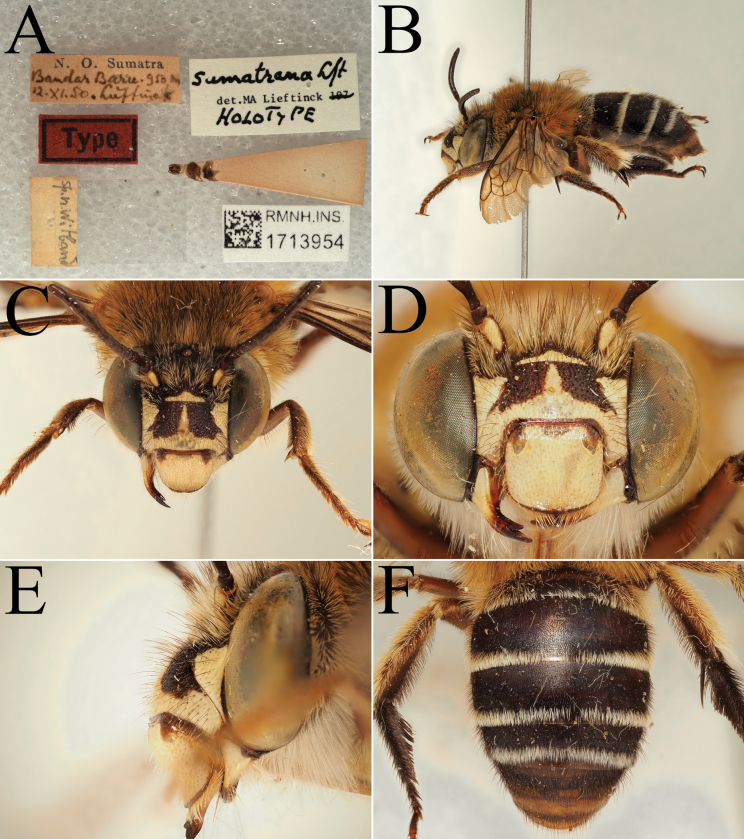
Holotype of *A. sumatrana* Lieftinck, 1956 (RMNH). A. Labels of the specimen; B. Habitus in profile view; C. Face in frontal view; D. Labrum in ventral view; E. Protuberance of the clypeus in profile view; F. Terga in dorsal view.

14. Amegilla (Glossamegilla) pagdeni Lieftinck, 1956

15. Amegilla (Glossamegilla) pendleburyi (Cockerell, 1929) (Fig. 22)

16. Amegilla (Glossamegilla) proboscidea Lieftinck, 1956 (Fig. 23)

17. Amegilla (Glossamegilla) sumatrana Lieftinck, 1956 (Fig. 24)

18. Amegilla (Glossamegilla) suzanneae Carion & Wood, sp. nov.

19. *Amegilla* (?*Glossamegilla*) *tetrataeniata* (Gribodo, 1894)

20. Amegilla (Glossamegilla) vigilans (Smith, 1860)

### ﻿Biogeography of the subgenus Glossamegilla in the Indo-Australian Archipelago

**Figure 25.**
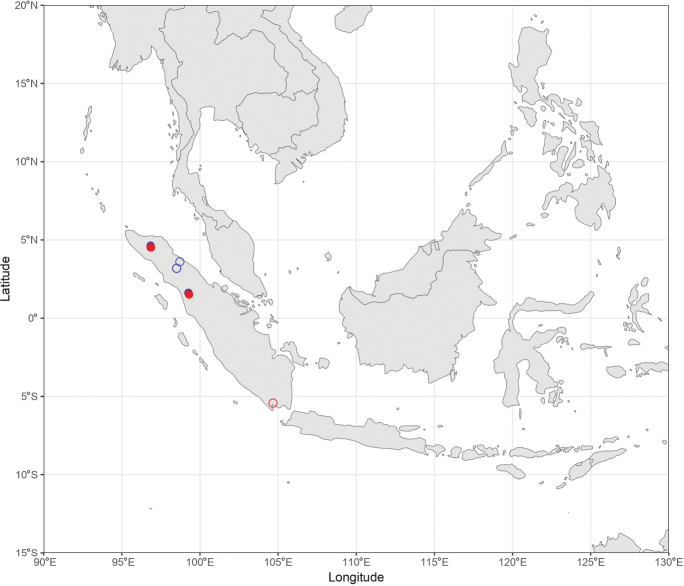
Distribution map for *A. amymone* (Bingham, 1896) in the Indo-Australian Archipelago. This map contains both colour forms of *A. amymone* with the dark form (true *amymone*) represented by the blue points and the pale form (*A. bouwmani* (Lieftinck, 1944) syn. nov.) represented by the red points. The empty points represent specimens collected before and in 1950 while the full points represent specimens collected in 1951 and thereafter.

As seen in Fig. 25, both colour forms *A. amymone* seem clearly restricted to the island of Sumatra. The majority of specimens were found in the north of the island while only one specimen of the pale form (holotype of *A. bouwmani*) was found in the south. This species is a mountain species with a minimum known elevation of 1400 meters (Table 8).

**Table 8.** Elevational range for each species of the subgenus Glossamegilla of the Indo-Australian Archipelago. In the third column, a + represents an expansion in the known altitude to a higher altitude compared to the altitudes given by Lieftinck in 1956, a – represents an expansion in the known altitude to a lower altitude, an = represents an absence of change in the known altitude and a / represent the fact that the species was not considered by the paper of Lieftinck (1956). The column “Distribution” revises the known distribution for each species by considering the islands/country where the species can be found.

Species	Elevation in meters	Elevational distribution relative to Lieftinck (1956)	Notes	Distribution
*Amegilla amymone* (Bingham, 1896)	1400-200	+	*Amegilla bouwmani* included.	Sumatra
*Amegilla anthracina* (Gribodo, 1894)	0-50	/	Not considered by Lieftinck (1956). Inferred from the elevational range of Tanimbar.	Tanimbar Islands
*Amegilla celineae* Carion, sp. nov.	0-100	/	Based only on the holotype. Altitude inferred from the locality.	Borneo (Indonesian part)
*Amegilla cinnyris* (Lieftinck, 1944)	350-650	+ & -		Sumatra
*Amegilla cyrtandrae* (Lieftinck, 1944)	550-1900	-		Java, Bali
*Amegilla elephas* (Lieftinck, 1944)	0-1400	+		Sumatra, Continental Malaysia
*Amegilla feronia* (Lieftinck, 1944)	0-800	=		Java
*Amegilla floresiana* Carion & Wood, sp. nov.	1200-1300	/	Based only on the type series.	Flores
*Amegilla gigas* (Friese, 1922)	450-600	/	Based on the only known male specimen. Species not considered by Lieftinck (1956).	Timor, Wetar
*Amegilla hanitschi* (Meade-Waldo, 1914)	600-1550	+		Sumatra, Continental Malaysia
*Amegilla himalajensis* (Radoszkowski, 1882)	0-1100	=		Laos, Thailand, Sumatra, Continental Malaysia
*Amegilla insularis* (Smith, 1857)	0-1550	+		Sumatra, Continental Malaysia, Borneo (all the island)
*Amegilla jacobi* (Lieftinck, 1944)	0-1000	+		Java
*Amegilla pagdeni* Lieftinck, 1956	0-1100	+		Sumatra, Continental Malaysia
*Amegilla pendleburyi* (Cockerell, 1929)	0-1100	=		Sumatra, Continental Malaysia, Borneo (all the island)
*Amegilla proboscidea* Lieftinck, 1956	0	=	Based only on the holotype. Altitude inferred by Lieftinck (1956).	Simalur
*Amegilla sumatrana* Lieftinck, 1956	950-1400	=		Sumatra
*Amegilla suzanneae* Carion & Wood, sp. nov.	150-1600	/	Based only on the type series (3 specimens).	Borneo (Malaysian parts)
*Amegilla tetrataeniata* (Gribodo, 1894)	0-3000	/	Not considered by Lieftinck (1956). Inferred from the elevational range of Timor, highly imprecise.	Timor
*Amegilla vigilans* (Smith, 1860)	0-1200	/	Not considered by Lieftinck.	Sulawesi

**Figure 26.**
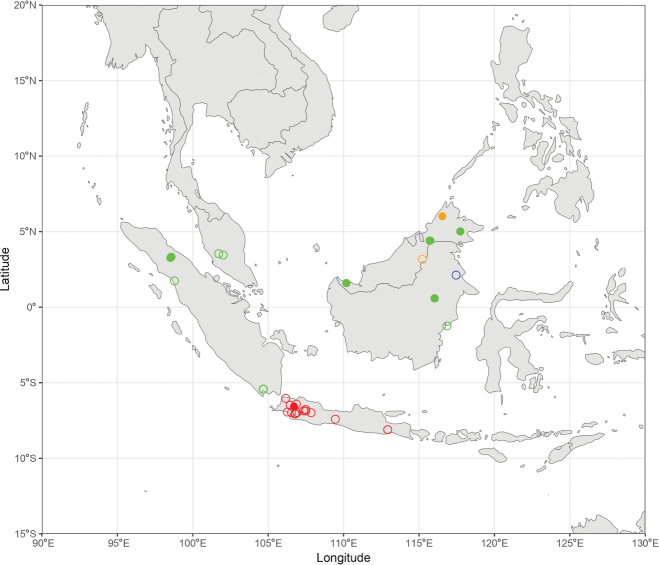
Distribution map for four morphologically close species in the Indo-Australian Archipelago: *A. celineae* Carion, sp. nov. (blue points), *A. feronia* (Lieftinck, 1944) (red points), *A. pendleburyi* (Cockerell, 1929) (green points), and *A. suzanneae* Carion & Wood, sp. nov. (orange points). The empty points represent specimens collected before and in 1950 while the full points represent specimens collected in 1951 and after.

*Amegilla pendleburyi* is the most widespread of its group of morphologically close species, all represented on Fig. 26. It is distributed in Sumatra, the continental part of Malaysia and in Borneo (both Malaysian and Indonesian parts of the island). On the island of Borneo, *A. suzanneae* sp. nov. is distributed in the northern part (Malaysian part of Borneo, in the region of Sabah and in the North of the region of Sarawak) while *A. celineae* sp. nov. is distributed in the Indonesian part of Borneo, in the East of the island. Finally, *A. feronia* seems restricted to the Island of Java and mainly distributed on the West of the island.

*Amegilla celineae* sp. nov. and *A. suzanneae* sp. nov. are, based on currently available information, both restricted to the island of Borneo and most likely occur in sympatry with *A. pendleburyi* (Fig. 26). It is currently unknown whether they occur sympatrically. Based on the morphological similarities between the three species, they are thought to be phylogenetically closely related and to come from a same ancestor that speciated probably in Borneo.

**Figure 27.**
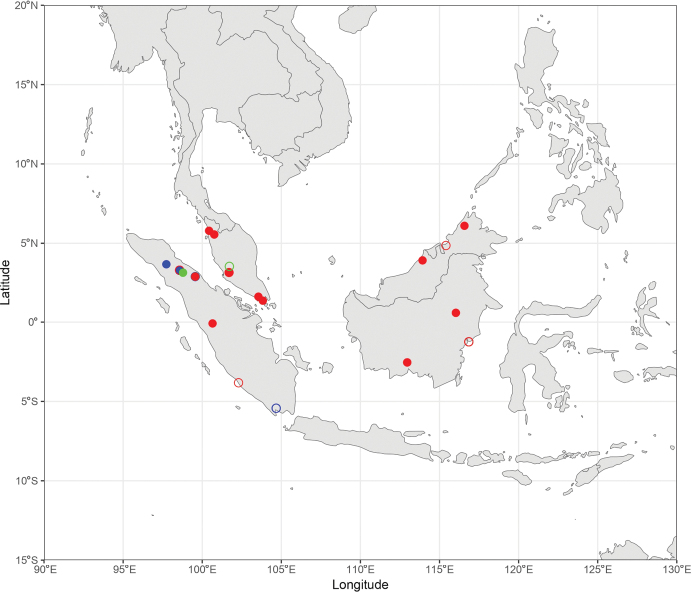
Distribution map for three morphologically close species in the Indo-Australian Archipelago: *A. cinnyris* (Lieftinck, 1944) (blue points), *A. insularis* (Smith, 1857) (red points) and *A. pagdeni* (Lieftinck, 1944) (green points). The empty points represent specimens collected before and in 1950 while the full points represent specimens collected in 1951 and thereafter.

In the group of morphologically close species containing *A. cinnyris*, *A. insularis*, and *A. pagdeni*, it is *A. insularis* is the most widespread with a distribution in Sumatra, Malaysia (continental), and in Borneo (Malaysian and Indonesian parts) (Fig. 27). *Amegilla pagdeni* is distributed in northern Sumatra and in Malaysia (continental) while *A. cinnyris* is restricted to the island of Sumatra (Fig. 27).

The known distribution of *A. pagdeni* is enlarged to the island of Sumatra compared to the distribution given by Lieftinck (1956) thanks to 12 specimens (RMNH) collected in 1996 by R. Desmier de Chenon. Moreover, this species was considered to be coming from Siam (Thailand) in the summary table of the biogeography of this group by Lieftinck (1956). However, the materials examined by Lieftinck in this same paper all come from continental Malaysia and Lieftinck’s listing is therefore erroneous.

**Figure 28.**
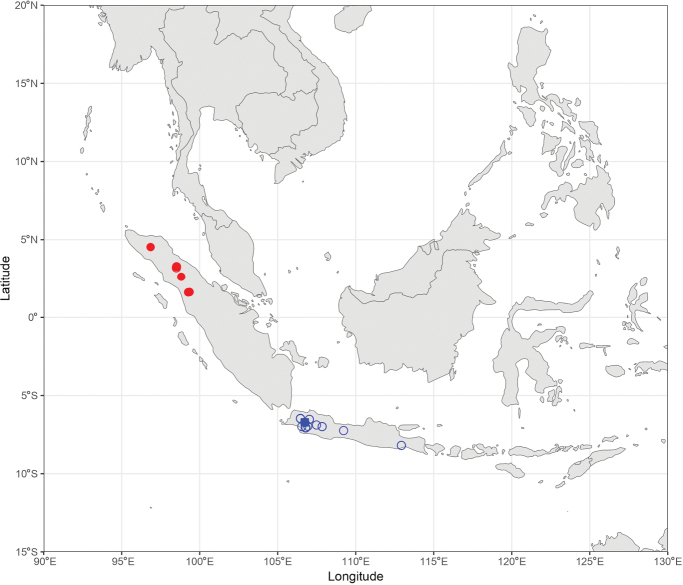
Distribution map for two morphologically close species in Indo-Australian Archipelago: *A. jacobi* (Lieftinck, 1944) (blue points) and *A. sumatrana* Lieftinck, 1956 (red points). The empty points represent specimens collected in 1950 and before, the full points represent specimens collected before and in 1951 and squares represent specimens with an unknown collection date.

In the group of *A. sumatrana* and *A. jacobi*, *A. sumatrana* seems to be restricted to north Sumatra while *A. jacobi* seems to be restricted to Java (mainly distributed in west Java (Fig. 28).

**Figure 29.**
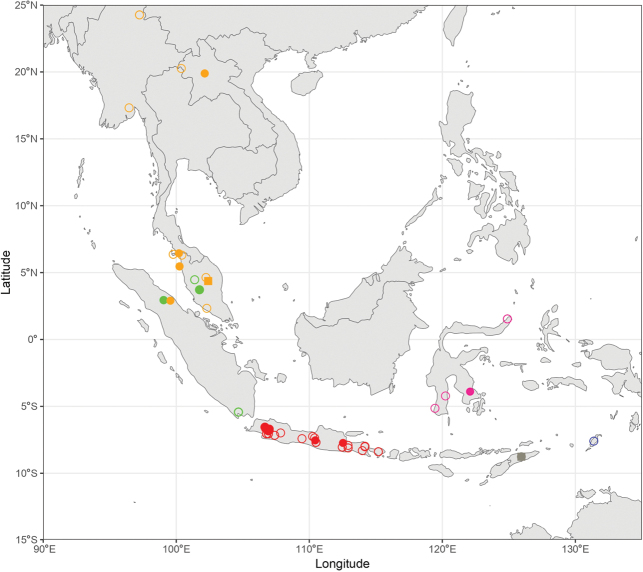
Distribution map for six species in the Indo-Australian Archipelago: *A. anthracina* (Gribodo, 1894) (blue points), *A. cyrtandrae* (Lieftinck, 1944) (red points), *A. hanitschi* (Meade-Waldo, 1914) (green points), *A. himalajensis* (Radoszkowski, 1882) (orange points), *A. tetrataeniata* (Gribodo, 1894) (grey points) and *A. vigilans* (Smith, 1860) (rose points). The empty points represent specimens collected before and in 1950 the full points represent specimens collected in 1951 and after, and squares represent specimens with an unknown collection date.

Fig. 29 shows the distribution of *A. himalajensis* a widespread species distributed from Laos and Thailand to northern Sumatra and including continental Malaysia. *Amegilla hanitschi* is distributed in continental Malaysia as well as in Sumatra (across all the island) (Fig. 29). Concerning *A. cyrtandrae*, this species is distributed mainly in Java but can also be found in Bali (to the east of Java) (Fig. 29). *Amegilla anthracina* seems, with the current information, restricted on the islands of Tanimbar while *Amegilla tetrataeniata* seems restricted to the island of Timor. Finally, *A. vigilans* is distributed in the island of Sulawesi, the terra typica, on the eastern side of the Wallace Line and seems to be found all over the island (Fig. 29; Smith 1860). Indeed, the type is known from Makassar, in the south-west of the island while the species seems to be more widespread overall based on the new data available (Fig. 29; Smith 1860). With the current biogeographic information of the species, *A. vigilans* can be considered as restricted to Sulawesi while also being the only known *Glossamegilla* of the island.

The known distribution of *A. himalajensis* is enlarged, similarly to *A. pagdeni*, to the island of Sumatra compared to the distribution given by Lieftinck (1956) thanks to one specimen (RMNH) coming from north-east Sumatra, near the Malaysian peninsula collected by R. Desmier de Chenon in 1996. Moreover, two other specimens, collected by Hagen on an unknown date were determined by Lieftinck in 1955 and 1956 (Lieftinck 1956: 21). The locality labels of the two specimens stated “?Sumatra”, re-enforcing the possibility of the presence of this species on the island and, therefore, in Indonesia. *Amegilla himalajensis*, as well as *A. pagdeni*, may have been overlooked in the past due to a lack of recording on Sumatra, the northern mountainous areas of which can be challenging to sample.

**Figure 30.**
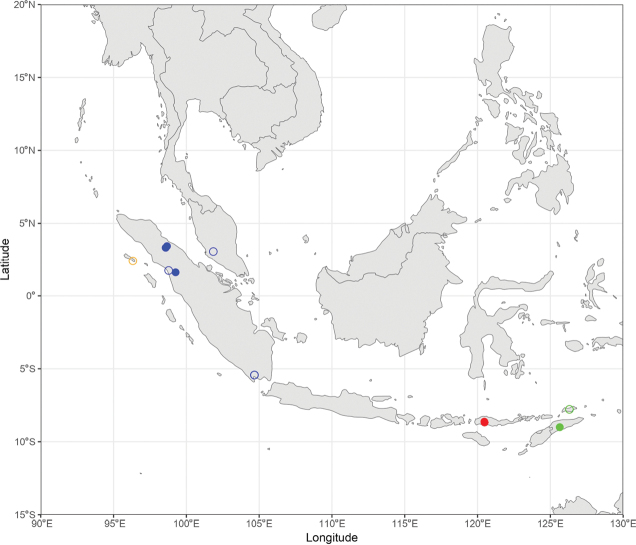
Distribution map for four species in the Indo-Australian Archipelago: *A. elephas* (Lieftinck, 1944) (blue points), *A. floresiana* Carion & Wood, sp. nov. (red points), *A. gigas* (Friese, 1922) (green points) and *A. proboscidea* Lieftinck, 1956 (orange points). The empty points represent specimens collected before and in 1950 while the full points represent specimens collected in 1951 and after.

*Amegilla proboscidea* is restricted to the island of Simalur, on the western side of Sumatra (Fig. 30) but *A. elephas* is distributed across Sumatra and in continental Malaysia (Fig. 30). On the eastern side of the Wallace Line, two more species can be found, *A. floresiana* sp. nov. which is mainly distributed on the island of Flores as well as *A. gigas* that can be found on the island of Timor (suspected male, Fig. 30) but also on the island of Wetar (for the lectotype previously designated; Friese 1922).

### ﻿Revision of Lieftinck’s (1956) key

13 antennal articles, 7 terga…Males

12 antennal articles, 6 terga…Females

#### ﻿Key to the males

**Table:** 

1	Body length of 19 mm or more. First 3 terga basally with black tomentum/pilosity and fulvous hair bands along the posterior margin (Fig. 19F). In profile view, anterior margin of clypeus projecting beyond anterior margin of compound eye by a distance greater than the diameter of the compound eye (Fig. 19E)	***A. elephas* (Lieftinck, 1944)**
–	Body length not exceeding 18 mm. Pubescence variable. Face variable, anterior margin of clypeus either projecting or clearly projecting by less than the diameter of the compound eye	**2**
2	Dorsal part of the metasoma with short emerald to turquoise pilosity evenly distributed on the terga. Dorsal pubescence of the mesosoma with black, grey, and pale green hairs. Labrum basally with a brown stripe, the remainder ivory white while the clypeal and mandibular markings are yellow	***A. hanitschi* (Meade-Waldo, 1914)**
–	Dorsal part of the metasoma without any green pilosity, the pubescence is generally pale (white to fulvous) to dark (dark brown to black) or a mix of both (Figs 6F, 9F, 10F, 11F, 12D, 13D, 17, 18F, 20F, 21F, 22F, 23F, 24F)	**3**
**3**	At least terga 2–4 with poorly to strongly defined pale hair bands on their apical margins, these contrasting the darker hairs of the tergal discs ; for some species the bands are incomplete in the centre (Fig. 21F); the hair bands can be wide or narrow; the tergal discs are generally clothed with black or darker pubescence and in some species, T1 mostly to entirely covered with pale pubescence (Figs 6F, 10F, 11F, 18F, 20F, 21F, 24F)	**4**
–	Terga without any hair bands on their marginal areas or if pubescent then this not strongly contrasting in the surrounding pilosity; either with an entirely (sometimes evenly) distributed pale pubescence on the first 4 terga or with other types of pubescence (Figs 14E, F, 17F, 22D)	**10**
4	Metasoma mainly black-haired with very narrow sharply defined white to slightly fulvous hair bands; the hair bands can be interrupted in the centre or not (Figs 21F, 24F)	**5**
–	Metasoma with broader hair bands and fewer dark hairs on the tergal discs; hair bands generally with darker colourations (fulvous to ochraceous-orange but never white) (Figs 6F, 10F, 11F, 18F, 20F)	**6**
5	Terga 1–4 apically with complete, narrow, and sharply defined bands of short hairs that can be whitish to slightly fulvous; T1 with longer hairs of the same colours that expand from the sides of the tergum to the centre (pubescence more dense on the side than on the centre); sides of T2-3 with tuft of whitish to fulvous hairs that do not expand (Fig. 24F)	***A. sumatrana* Lieftinck, 1956**
–	T2-4 apically with incomplete hair bands of whitish to fulvous hairs, except sometimes complete only on T4; hair band on T1 limited to only a few short pale hairs that are hardly visible; T1 with longer whitish to slightly fulvous hairs on the sides that do not expand very much; T2-3 without tuft of paler hairs (Fig. 21F)	***A. jacobi* (Lieftinck, 1944)**
6	Larger species, ~18 mm. Clypeus strongly protuberant; in profile view, anterior margin of clypeus projecting beyond anterior margin of compound eye by a distance of approximately the diameter of the compound eye or more (Figs 6E, 9E, 11E). Apical hair bands usually paler in colours, ochraceous, but sometimes fulvous to bright orange (Figs 6F, 10F, 11F)	**7**
–	Smaller species, with a maximum of 15–17 mm. Clypeus less protuberant; in profile view, anterior margin of clypeus projecting beyond anterior margin of compound eye by a distance smaller than the diameter of the compound eye (Figs 18E, 20E). Apical hair bands darker, fulvous to bright orange but never ochraceous (Figs 18F, 20F)	**9**
7	Labrum slightly wider than long (17:16–19:18); punctation of the clypeus fine, not coarse, and very sparse with large interspaces (at least the diameter of a puncture) (Fig. 6D). Hair bands on the apex of metasoma wider and very poorly defined with a small basal part with black hairs or black hairs absent (Fig. 6F). Pilosity variable, ochraceous to bright orange (Fig. 6B)	***A. floresiana* Carion & Wood, sp nov.**
–	Labrum as long as wide (1:1); punctation of the clypeus coarse and not very sparse, rather with smaller interspaces (at most the diameter of a puncture) (Figs 9D, 11D). Hair bands on the apex of metasoma narrower and more sharply defined with a larger basal part with black hairs that are always present (Figs 10F, 11F). Pilosity ochraceous to very slightly orange (Figs 10B, 11B)	**8**
8	Clypeus more protuberant; in profile view, anterior margin of clypeus projecting beyond anterior margin of compound eye by a distance greater than the diameter of the compound eye (~1.3 compound eye diameter) (Fig. 11E). Pale clypeal mark yellow and very expanded, 1 small black to slightly reddish mark and 1 thin brown line on each side of the carina that resume the black clypeal marks (Fig. 11C). Pilosity in general more bright, fulvous to slightly orange (Fig. 11B, F). Dorsal pubescence of the mesosoma without or with only few black hairs intermixed (Fig. 31A)	***A. gigas* (Friese, 1922)**
–	Clypeus less protuberant; in profile view, anterior margin of clypeus projecting beyond anterior margin of compound eye by a distance of approximately the diameter of the compound eye (Fig. 9E). Pale clypeal marks more orangish and less expanded with the presence of 2 subrectangular dark clypeal mark (1 on each side of the carina) (Fig. 10D). Pilosity in general less bright, rather ochraceous (Fig. 10B, F). Dorsal pubescence of the mesosoma with more black hairs intermixed (Fig. 31B)…(partim, dark form)	***A. vigilans* (Smith, 1860)**
9	Pale clypeal marks ivory-white and covering a larger surface of the clypeus (Fig. 18C). Paraocular areas less protruding (Fig. 18E). Labrum square-shaped, as long as wide (Fig. 18D). Tergal hair bands broad, thus dark hairs occupying relatively small area of terga (Fig. 18F). Tergal margins predominantly black with the rim narrowly paler hyaline-brown, never extensively coloured orange or pale brown, any colouration obscured by and barely showing through the pubescence. Dorsal pubescence of the mesosoma paler and with many black hairs intermixed (Fig. 32A)	***A. cyrtandrae* (Lieftinck, 1944)**
–	Pale clypeal marks yellow and covering a smaller surface of the clypeus (Fig. 20C). Paraocular areas more protruding (Fig. 20E). Labrum rectangular-shaped, clearly longer than wide (Fig. 20D). Tergal hair bands relatively narrow, thus dark hairs appearing abundant on the tergal discs (Fig. 20F). Tergal margins normally extensively paler orangish to pale brown, visible through and around the pubescence (the integument can be decoloured in collection specimen). Dorsal pubescence of the mesosoma less pale, more orange, with fewer black hairs intermixed (Fig. 32B)	***A. feronia* (Lieftinck, 1944)**
10	Dark clypeal marks reddish to orangish, not very sharply defined compared to the orangish yellow pale marks and never deep black (Fig. 33A). Dorsal pubescence of mesosoma bright orange-rufous, without black hairs intermixed (Fig. 33B). First 2 terga partly or wholly covered with fulvous hairs (sometimes the sides of the T3 with small tuft), the remaining terga black-haired	***A. himalajensis* (Radoszkowski, 1914)**
–	Dark clypeal marks neither reddish nor orangish, rather deep black to only very slightly reddish, with their outer margins sharply defined (Figs 14C, D, 17C, 22C). Dorsal pubescence of mesosoma generally less bright with at least some black hairs intermixed (Figs 14A, B, 17B, 22B)	**11**
11	Metasoma with the apical terga (from T4 onwards) entirely with apricot-orange pubescence (sometimes T4 only 1/2 covered by this pubescence), T1-3 variable in pubescence but always abundant black hairs on the discs of T1-3 (Fig. 14E, F). Dorsal pubescence of the mesosoma black to very dark brown or even slightly ochraceous (Fig. 14A, B). Clypeus mainly ivory-white and with the pale median marking large, without defined sub-rectangular dark marks (Fig. 14C. D). Restricted to Sumatra	**12**
–	Terga typically without distinctive apricot-orange pubescence on T4-6, either because all terga are uniformly covered with orange pubescence, or pale pubescence is more sombre or pale (Figs 9F, 17F, 22D). Dorsal pubescence of the mesosoma paler, never black or dark brown and generally brighter than ochraceous (Figs 9B, 17B, 22B, 31B). Clypeus with well-defined sub-rectangular dark marks, the central medial marking therefore smaller and more restricted (Figs 9C, 17C, 22C). Distribution variable	**13**
12	T1-3 predominantly black-haired, with only hints of apricot-orange pubescence (Fig. 14E). Dorsal pubescence of mesosoma rather black to very dark brown (Fig. 14A). Generally T4 basally clothed with black pilosity and apically with apricot-orange pubescence (Fig. 14E)… dark form	***A. amymone* (Bingham, 1896)**
–	T1-3 apically with apricot-orange pilosity and generally all basally covered with black pubescence (Fig. 14F). Dorsal pubescence of mesosoma brighter, mixture of black, brown, and ochraceous hairs (Fig. 14B). T4 and after entirely covered with apricot-orange pubescence (Fig. 14F)… pale form (*bouwmani*)	***A. amymone* (Bingham, 1896)**
13	Clypeus more protuberant; in profile view, anterior margin of clypeus projecting beyond anterior margin of compound eye by a distance of approximately the diameter of the compound eye (Fig. 9E). Basitarsus III without any black pilosity, entirely clothed with pale pubescence that can be either ochraceous/slightly yellow or fulvous/slightly orange (Figs 9B, 22B)	**14**
–	Clypeus less protuberant; in profile view, anterior margin of clypeus projecting beyond anterior margin of compound eye by a distance smaller (at least slightly) than the diameter of the compound eye (Fig. 17E). Basitarsus III at least partly, if not entirely, covered by black pubescence (Fig. 17B)	**15**
14	Pale clypeal marks more extended and slightly salmon compared to the next species (Fig. 9C). Pilosity in general paler, rather ochraceous to slightly yellow (including the outside of basitarsus III) (Fig. 9B). Terga entirely clothed with pale ochraceous pilosity that is denser apically, giving slightly contrasting poorly-defined hair bands (Fig. 9F). Restricted to Sulawesi... (partim, pale form)	***A. vigilans* (Smith, 1960)**
–	Pale clypeal marks less extended and yellow (Fig. 22C). Pilosity brighter, more fulvous-orange (including the outside of basitarsus III) (Fig. 22B). Terga entirely and more evenly clothed with a bright fulvous-orange pilosity, leading to not really contrasting hair bands (Fig. 22D). Distributed on Sumatra, continental Malaysia, and all across Borneo	***A. pendleburyi* (Cockerell, 1929)** (NB, take care, as specimens on Borneo may belong to 1 of the 2 newly described species morphologically close to *A. pendleburyi*)
15	Clypeus less protuberant, anterior margin of clypeus projecting by ~1/2 the diameter of the eye (Fig. 34A). Pale clypeal marks ivory-yellow and covering a slightly smaller surface of the clypeus compared to the next 2 species (Fig. 34B). Transverse carina at the apex of the labrum narrowly emarginate medially, laterally with 2 small protruding tubercles (Fig. 34C). Pale pubescence covering entirety if T1-5, hairs on remaining terga deep black. General appearance wider and more robust	***A. insularis* (Smith, 1857)**
–	Clypeus more protuberant, anterior margin of clypeus projecting by slightly less than the diameter of the eye but more than 1/2 the diameter (Fig. 17E). Pale clypeal marks ivory-white or maize-yellow to buff-yellow and covering a slightly larger surface of the clypeus compared to *insularis* (Figs 17C, 34F). Transverse carina at the apex of the labrum without a central depression, if 1, not surrounded by 2 protruding teeth (Fig. 34D, E)	**16**
16	Pale face marks maize-yellow or buff-yellow (Fig. 34F). T3-4 partly, the succeeding terga entirely, covered with black hairs (sometimes hardly visible on T3 but generally well visible on T4)	***A. pagdeni* Lieftinck, 1956**
–	Pale face marks ivory-white (Fig. 17C). T1-5 entirely covered with orangish pubescence, the succeeding terga, occasionally also part of the T5, black-haired (generally T6 and succeeding hardly visible because they are hidden by T5) (Fig. 17F)	***A. cinnyris* (Lieftinck, 1944)**

**Figure 31.**
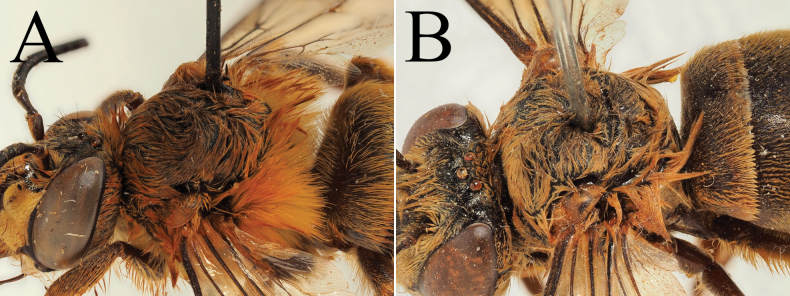
Dorsal pubescence of the mesosoma seen in profile view. A. Mesosoma of one *Amegilla gigas* (Friese, 1922) male (RMNH); B. Mesosoma of one *Amegilla vigilans* (Smith, 1860) male (RMNH).

**Figure 32.**
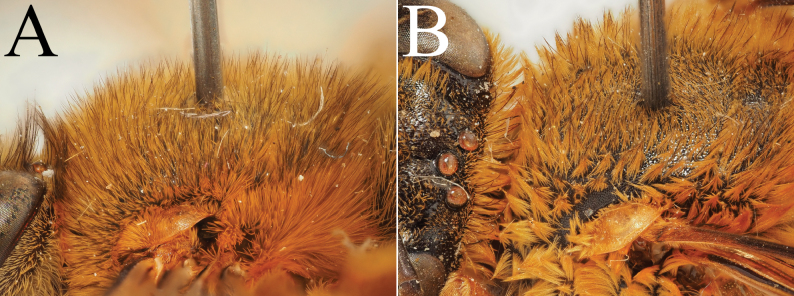
Dorsal pubescence of the mesosoma seen in profile view. A. Mesosoma of one *Amegilla cyrtandrae* (Lieftinck, 1944) female (RMNH); B. Mesosoma of one *Amegilla feronia* (Lieftinck, 1944) female (RMNH).

**Figure 33.**
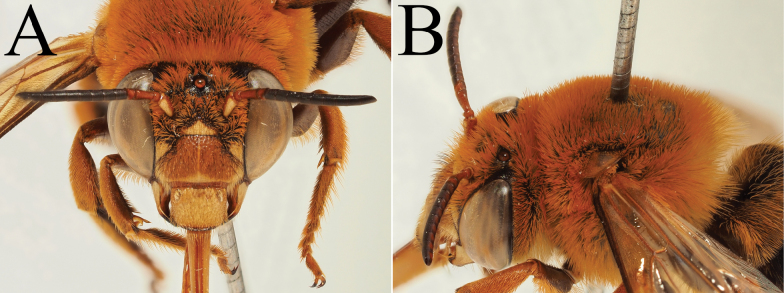
Two general views of one *Amegilla himalajensis* (Radoszkowski, 1882) male (RMNH) to illustrate key characteristics of the species. A. Face of the specimen seen in frontal view; B. Dorsal pubescence of the mesosoma seen in profile view.

**Figure 34.**
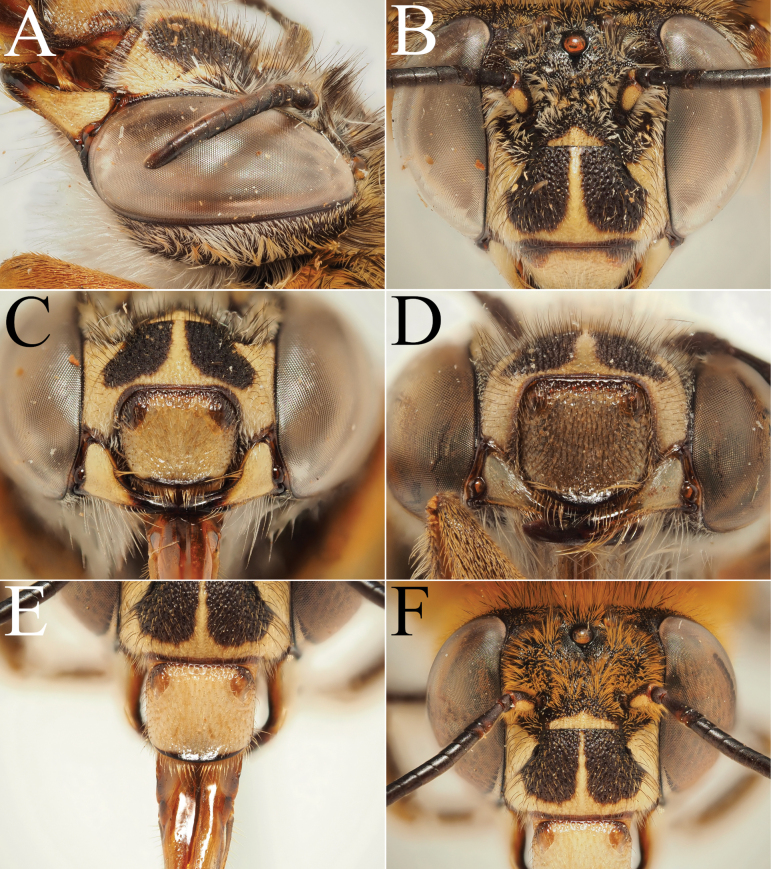
Several views of one *Amegilla insularis* (Smith, 1857) male (RMNH), one *Amegilla cinnyris* (Lieftinck, 1944) female (RMNH), and one *Amegilla pagdeni* Lieftinck, 1956 male (RMNH) to illustrate key characteristics of these species. A, B, C. Images of *A. insularis*; D. Image of *A. cinnyris*. E, F. Images of *A. pagdeni*; A. Head in profile view showing the protuberance of the clypeus. B, F. Face in frontal view showing the clypeal marks. C, D, E. Labrum in ventral view showing the difference of emargination on the carina at the apex.

#### ﻿Key to the females

**Table:** 

1	Body length of 21 mm or more	**2**
–	Body length not exceeding 19 mm	**3**
2	Pale clypeal marks lemon-chrome, sometimes slightly orangish, dark clypeal marks slightly brownish/reddish black to only black and sharply defined (Fig. 19C). T1-4 basally clothed with black hairs and apically clothed with wide yellow to slightly orange hair bands (more extended at the centre on T3-4), remaining terga entirely covered by the yellow-orange pubescence (Fig. 19F). Distributed in Malaysia and Sumatra	***A. elephas* (Lieftinck, 1944)**
–	Pale clypeal marks cinnamon-rufous, dark clypeal marks brownish reddish and poorly defined (Fig. 23C). Terga mainly black-haired, except for narrow orange hair bands at the apex of the first 4 terga and an apical orange hair fringe at the apex of T5 (Fig. 23F). Restricted to Sumatra (only known from Lasikin on the island of Pulau Simeulue)	***A. proboscidea* Lieftinck, 1956**
3	Dorsal surface of metasoma evenly covered by short bright green pilosity (emerald to golden green) that tend to yellow in collection due to decolouration. Pale clypeal mark restricted to only an inverted T-shaped yellow mark. Pubescence of the tibia III 1/2 ochraceous-orange (upper part) and 1/2 black (lower part) on the outer surface of the tibia. Green hairs are present on the dorsal part of the mesosoma and on the head, mixed with hairs of other colours	***A. hanitschi* (Meade-Waldo, 1914)**
–	Dorsal part of the metasoma without any green pilosity, the pubescence is generally pale (white to fulvous) to dark (dark brown to black) or a mixture of both type of colouration; hair bands present or not (Figs 5F, 7F, 8F, 9F, 10F, 12D, 13D, 17F, 18F, 20F, 21F, 22D, 24F)	**4**
4	At least T2-4 with very contrasting poorly-defined to sharply-defined hair bands on the posterior margin; for some species the bands are incomplete in the centre; the hair bands can be wide or thin; the terga are generally basally clothed with black or darker pubescence and sometime T1 is entirely clothed with pale pubescence (Figs 5F, 7F, 8F, 10F, 18F, 20F, 21F, 24F)	**5**
–	Terga without any hair bands on their posterior margins, sometimes most terga entirely covered by pale pubescence that is slightly denser apically, leading to hair bands that are not very contrasted compared to the surrounding pilosity; metasoma either with an entirely (sometimes evenly) distributed pale pubescence on the first 4 terga or with other type of pubescence (Figs 8F, 9F, 12D, 13D, 17F, 22F)	**12**
**5**	Metasoma mainly black-haired with very thin sharply defined white to slightly fulvous hair bands; hair bands can be interrupted in the centre or not depending on the species (Figs 21F, 24F)	**6**
–	Metasoma with fewer black hair, with wider and more poorly-defined hair bands at the apex; hair bands generally with darker colours (fulvous to ochraceous-orange but never white) (Figs 5F, 7F, 10F, 18F, 20F)	**7**
6	First 4 terga with complete white to slightly fulvous hair bands; T1 entirely covered with sparse and long fulvous hairs; sides of T2 (and occasionally T3) with a fulvous hair tuft (Fig. 24F). Sometimes the habitus looks wider than in *A. jacobi* but both species are generally similar in width	***A. sumatrana* Lieftinck, 1956**
–	T1-4 with incomplete white to slightly fulvous hair bands at the apex, sometimes complete to nearly complete on the T4 and restricted to only few sparse hairs on the T1; T1 with sparse long fulvous hairs that are interrupted at the centre of the tergum; absence of fulvous hair tuft on the side of T2-3 (Fig. 21F). Sometimes the habitus is less wide than in *A. sumatrana* but both species are generally similar in width	***A. jacobi* (Lieftinck, 1944)**
7	Larger species, ~17–18 mm. Clypeus a bit more strongly protuberant; in profile view, anterior margin of clypeus projecting beyond anterior margin of compound eye by a distance of approximately or slightly greater than the diameter of the compound eye (Figs 5E, 9E)	**8**
–	Smaller species, restricted ~13–16 mm. Clypeus less strongly protuberant; in profile view, anterior margin of clypeus projecting beyond anterior margin of compound eye by a distance smaller (sometimes slightly) than the diameter of the compound eye (Figs 7E, 18E, 20E)	**10**
8	Carina of the clypeus more blunt, nearly flat, creating a large, impunctate and shining line at the centre of the clypeus. Pale clypeal marks less extended and yellowish (tending to dark yellow), face mainly dark. Paraocular areas with restricted ivory-white spots as well as a clear shining area between the punctation of the paraocular areas and the transition with the clypeus	***A. gigas* (Friese, 1922)**
–	Carina of the clypeus sharper and not flat; pale clypeal marks more extended and colouration vary from ivory-yellow to yellow-orange; paraocular areas variable in colouration but entirely punctuate (without a clear shining area) (Figs 5C, D, 35)	**9**
9	Hair bands rather sharply defined and slightly narrower, terga more covered by black pubescence (Fig. 10F). Pale clypeal marks more extended and yellow-orange; punctation of the clypeus coarser, less defined with smaller interspaces (generally ≤ 1/2 the diameter of a puncture); paraocular areas of the same colour than the clypeal marks and more extended (Fig. 35). Pubescence paler, ochraceous to slightly fulvous (Fig. 10B)…(partim, dark form)	***A. vigilans* (Smith, 1860)**
–	Hair bands rather poorly defined and wider with pale pubescence covering the majority of the terga (almost entirely for T4) (Fig. 5F). Pale clypeal marks less extended and ivory-yellow to yellow (Fig. 5C, D). Punctation of the clypeus finer, more defined with larger interspaces (generally the diameter of a puncture) (Fig. 5D). Paraocular areas of the same colour than the clypeal marks and less extended (Fig. 5C, D). Pubescence usually brighter, rather fulvous to orange (Fig. 5B)	***A. floresiana* Carion & Wood, sp. nov.**
10	Pale clypeal marks ivory-white to ivory-yellow and covering a slightly larger surface of the clypeus; scape dark (black) anteriorly (Fig. 36A). Dorsal pilosity of the mesosoma pale with many black hairs intermixed (Fig. 32A). Posterior margin of the terga black or at least very dark, never orangish or pale brown and hardly showing through the pubescence	***A. cyrtandrae* (Lieftinck, 1944)**
–	Pale clypeal marks ivory-yellow to orange and covering a slightly smaller surface of the clypeus; scape usually pale anteriorly, either orange or ivory-yellow, sometimes darker (dark brown) but never black (Figs 7C, D, 36B). Dorsal pilosity of the mesosoma bright (fulvous to bright orange generally) and with fewer dark hairs intermixed (Figs 7B, 32B). Posterior margin of the terga can be orangish to pale brown, accentuating the pubescence (can be decoloured in collection specimen)	**11**
11	Pale clypeal marks yellow, sometime even slightly orange to orange; scape orange anteriorly (Fig. 36B). Hair bands more sharply defined basally, black pilosity more extended (Fig. 20F). Restricted to Java	***A. feronia* (Lieftinck, 1944)**
–	Pale clypeal marks ivory-yellow to yellow; scape ivory-yellow to yellow also but sometimes dark (Fig. 7C, D). Hair bands less sharply defined basally, pale pilosity more extended and black pilosity more restricted (Fig. 7F). Restricted to Borneo	***A. suzanneae* Carion & Wood**, sp. nov.
12	All terga entirely and evenly covered by short, velvety, black to very dark brown pubescence. Pale clypeal mark darkened yellow on a deep black background. Metatibia covered by dark brown pubescence on its external surface. Restricted to Tanimbar	***A. anthracina* (Gribodo, 1894)**
–	Terga never entirely covered by black pubescence, at least some terga partly covered by coloured pubescence (Figs 8F, 9F, 12D, 13D, 17F, 22D). Clypeal mark variable (Figs 8C, 9C, 12C, 13C, 17C, 22C, 33A). Pubescence of the metatibia variable. Distribution variable but never found on Tanimbar	**13**
13	T3-4 apically with white, slightly wide, hair bands while the discs are covered by black and short pubescence. T1-2 and 5 entirely covered by black to very dark brown pubescence. Most of the head, scutellum, propodeum, external surface of protibia, 3/4 of mesotibia and dorsal 1/2 of the external surface of metatibia covered by white pubescence. Restricted to Timor	***A. tetrataeniata* (Gribodo, 1894)**
–	T3-4 without white hair bands or with no hair bands at all; pilosity of T1-5 variable (Figs 8F, 9F, 12D, 13D, 17F, 22D). No white hairs at all, except sometimes the tuft under the eye can be whitish. Distribution variable	**14**
14	Pale clypeal marks restricted to a small triangular paler mark at the apex (sometime with a narrow line of the same colour just above), face mostly dark (Figs 12C, 13C). Apricot-orange pubescence at least on T4-5 (Figs 12D, 13D). Restricted to Sumatra	**15**
–	Pale clypeal marks different, always more extensive (Figs 8C, 9C, 17C, 22C, 33A). Terga without apricot-orange pubescence, other types of pubescence (Figs 8F, 9F, 17F, 22D). Distribution variable	**16**
15	T1-3 almost entirely black-haired, at most with scattered apricot-orange hairs on tergal margins (Fig. 12D). Dorsal pubescence of mesosoma rather black to very dark brown (Fig. 12B). Generally T4 basally clothed with black pilosity and apically with apricot-orange pubescence, T5 entirely clothed by apricot-orange pubescence (Fig. 12D)…dark form	***A. amymone* (Bingham, 1896)**
–	T1-3 with apricot-orange pilosity, basally intermixed with black pubescence (Fig. 13D). Dorsal pubescence of mesosoma paler, rather ochraceous with black hairs intermixed (Fig. 13B). T4-5 entirely clothed with apricot-orange pilosity (Fig. 13D)…pale form (*bouwmani*)	***A. amymone* (Bingham, 1896)**
16	Dark clypeal marks red/brown, pale clypeal marks slightly paler yellow, thus these not strongly contrasting (Fig. 33A). Dorsal pubescence of the mesosoma bright orange to rufous, without black hairs intermixed (Fig. 33B). T1-2 partly or wholly covered with fulvous hairs (sometimes the sides of the T3 with small tuft), the remaining terga black-haired	***A. himalajensis* (Raoszkowski, 1882)**
–	Dark clypeal marks neither reddish nor orangish, rather deep black to only very slightly reddish; pale clypeal marks ivory-white to yellow, both type of marks hence strongly contrasting each other (Figs 8C, 9C, 17C, 22C). Dorsal pubescence of mesosoma generally less bright and always with at least some black hairs intermixed (Figs 8B, 9B, 17B, 22B). Terga different, at least entirely covered by pale pubescence of various colours on the first four segments (Figs 8F, 9F, 17F, 22D)	**17**
17	Clypeus more protuberant; in profile view, anterior margin of clypeus projecting beyond anterior margin of compound eye by a distance of approximately or slightly greater than the diameter of the compound eye (Figs 8E, 9E). Outside face of the hind basitarsus without black hairs, entirely covered by pale pubescence that can be ochraceous to slightly fulvous	**18**
–	Clypeus less protuberant; in profile view, anterior margin of clypeus projecting beyond anterior margin of compound eye by a distance smaller (sometimes slightly) than the diameter of the compound eye (Figs 17E, 34A). Outside face of the hind basitarsus at least partially, if not entirely, covered with black hairs	**20**
18	Pale clypeal marks more extended and more orangish than in the 2 next species (Fig. 10C, D). Pilosity in general paler, rather ochraceous to slightly yellow (including the outside of basitarsus III) (Fig. 9B). Terga entirely clothed with pale ochraceous pilosity that is denser apically, giving slightly contrasting poorly-defined hair bands (Fig. 9F). Hind tibiae usually dark, neither orange nor pale brown. Specimens generally ~19 mm…(partim, pale form)	***A. vigilans* (Smith, 1860)**
–	Pale clypeal marks less extended and yellow (Figs 8C, 22C, 37A). Pilosity brighter, more fulvous-orange (including the outside of basitarsus III) (Figs 8B, 22B). Terga usually entirely and more evenly clothed with a bright fulvous-orange pilosity, leading to not really contrasting hair bands but sometimes the hair bands are more visible on T3-4 (Figs 8F, 22D). Hind tibiae usually orange or pale brown, hardly visible with the pilosity. Specimens generally ~18–19 mm	**19**
19	Paraocular areas more punctuate (Fig. 37A). Labrum entirely and evenly punctuate (Fig. 37B). Punctations of the clypeus thinner with broader well-visible interspaces (Fig. 37A). Metasoma with a more even pubescence, apex of the terga usually without any contrasting hair bands (Fig. 22D). Smaller species (~18 mm). Mandibles bidentate and not very blunt	***A. pendleburyi* (Cockerell, 1929)**
–	Paraocular areas mainly impunctate, only a few punctures visible (Fig. 8E). Labrum not entirely punctuate, presence of some impunctate areas on the sides of the labrum, below the 2 protuberances (Fig. 8D). Punctations of the clypeus coarser with smaller, hardly visible, interspaces (Fig. 8C, D). Metasoma with a less even pubescence, hair bands at the apex of the terga more contrasting, T3-4 basally with black hairs (Fig. 8F). Larger species (~19 mm). Mandibles very blunt, bidentation hardly visible (beware, only 1 specimen found, can be variable)	***A. celineae* Carion, sp. nov.**
20	Larger species, between 15.0 and 17.5 mm. Clypeus less protuberant; in profile view, anterior margin of clypeus projecting beyond anterior margin of compound eye by a distance of ~1/2 the diameter of the compound eye (Fig. 34A). Pale clypeal marks ivory-yellow and covering a slightly smaller surface of the clypeus (especially compared to *A. cinnyris*) (Fig. 38). Transverse carina at the apex of the labrum narrowly emarginate medially, laterally with 2 small protruding tubercles (Fig. 34C). Pale pubescence entirely covering T1-5, hairs on remaining terga deep black	***A. insularis* (Smith, 1857)**
–	Smaller species, between 14 and 15 mm. Clypeus more protuberant; in profile view, anterior margin of clypeus projecting beyond anterior margin of compound eye by a distance slightly smaller than the diameter of the compound eye (Fig. 17E). Pale clypeal marks ivory-white or maize yellow to buff-yellow and covering a slightly larger surface of the clypeus (especially in *A. cinnyris*) (Fig. 39A, B). Transverse carina at the apex of the labrum without a central depression, if 1, not surrounded by 2 protruding tubercles (Fig. 34D, E)	**21**
21	Pale face marks maize yellow or buff-yellow (Fig. 39B). T1-3 entirely covered by fulvous pilosity, the T4 only apically covered by this pilosity and basally covered by black pilosity; remaining terga black-haired	***A. pagdeni* Lieftinck, 1956**
–	Pale face marks ivory-white (Fig. 39A). T1-4 entirely and evenly covered by fulvous pilosity, remaining terga black-haired	***A. cinnyris* (Lieftinck, 1944)**

**Figure 35.**
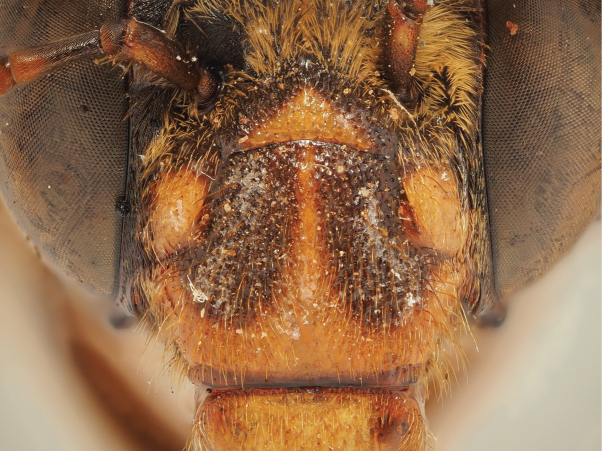
Face of a female of *Amegilla vigilans* (Smith, 1860) dark form (RMNH) showing the punctuation of the face.

**Figure 36.**
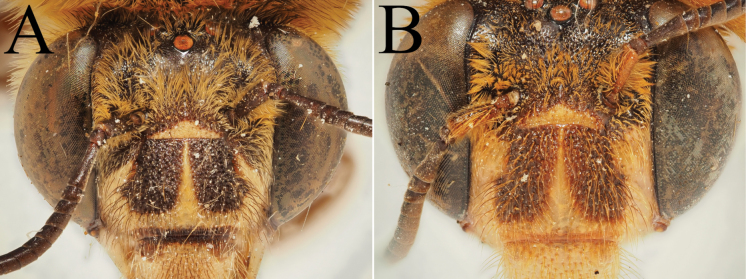
Face of two female species in frontal view. A. Face of *Amegilla cyrtandrae* (Lieftinck, 1944) (RMNH); B. Face of *Amegilla feronia* (Lieftinck, 1944) (RMNH).

**Figure 37.**
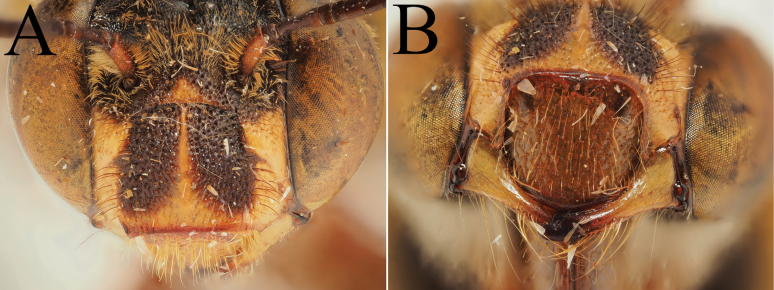
Two views of one *Amegilla pendleburyi* (Cockerell, 1929) female (RMNH) to illustrate key characteristics of the species. A. Face of the specimen with the punctuation of the clypeus and the paraocular areas; B. Labrum of the specimen with its punctuation.

**Figure 38.**
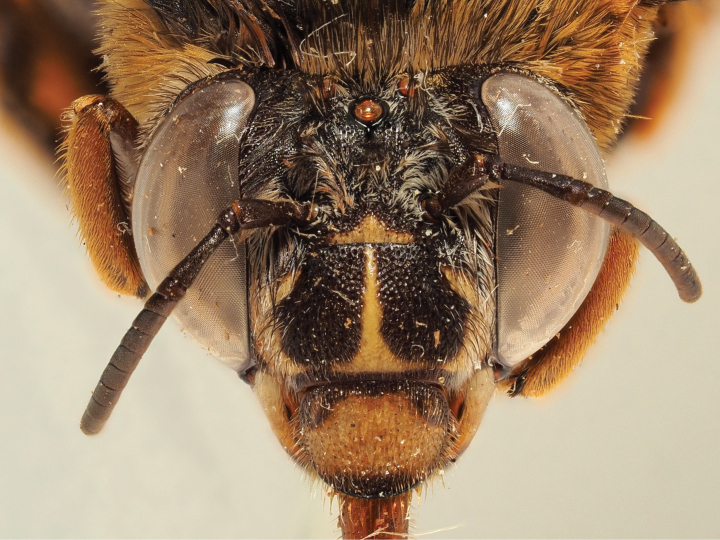
Face of one *Amegilla insularis* (Smith, 1857) (RMNH) to illustrate the clypeal marks of the females.

**Figure 39.**
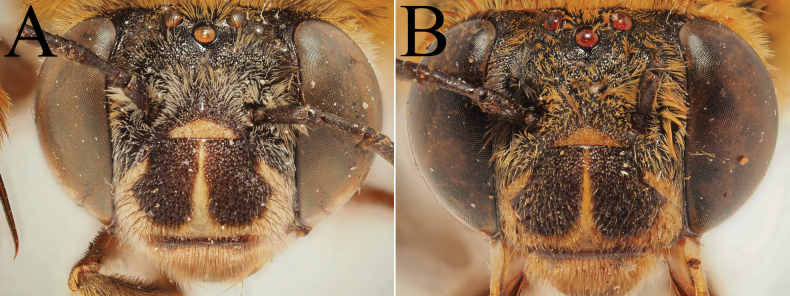
Face of two female species in frontal view. A. Face of *Amegilla cinnyris* (Lieftinck, 1944); B. Face of *Amegilla pagdeni* Lieftinck, 1956.

## ﻿Discussion

Compared to the baseline of Lieftinck (1956), the revisions presented here add four overlooked or alternatively classified species, three newly described species, two new country records, and one new synonymy, raising the number of species in the subgenus Glossamegilla known from the Indo-Australian Archipelago to 20.

**Figure 40.**
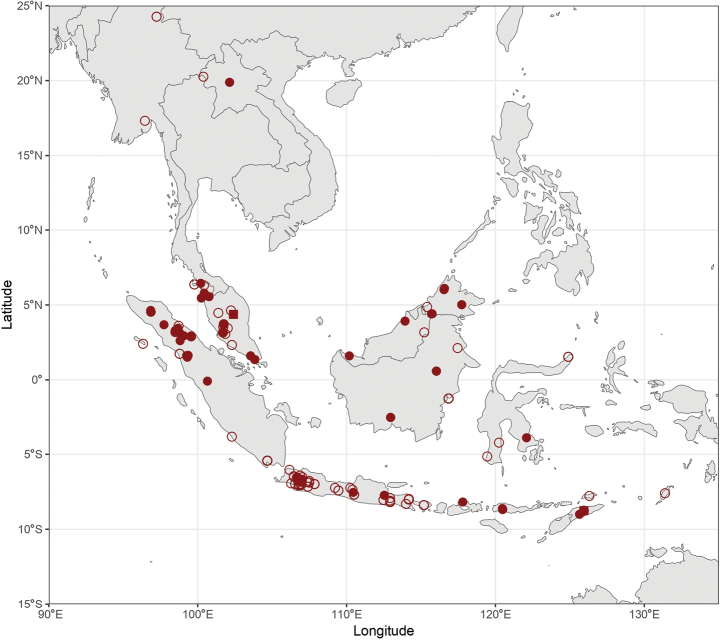
Distribution map with all the occurrences of the studied specimens from the subgenus Glossamegilla. This map allows one to have a better understanding of the distribution limits of the subgenus as well as the potential sampling biases. The empty points represent specimens collected before and in 1950, the full points represent specimens collected in 1951 and after, and squares represent specimens with an unknown collection date.

As seen on the maps of the species of *Glossamegilla* (Figs 25–30), only five species (namely *A. gigas*, *A. floresiana* sp. nov., *A. vigilans*, *A. anthracina*, and *A. tetrataeniata*) are found to the east of the original Wallace Line drawn in 1863. Given that the centre of diversity of Glossamegilla is to the west, this means that the subgenus most likely crossed the Lombok and Makassar straights at least once. However, even if the subgenus is present on both sides of the line, each individual *Glossamegilla* species seems to be restricted to only one side, meaning that members of the subgenus do not currently appear to display gene flow over this division. Moreover, the subgenus shows an eastern range limit on the islands of Tanimbar (with *A. anthracina*), meaning that, based on current information, *Glossamegilla* did not cross the Lydekker Line (Fig. 40), the eastern known line of separation between the Indo-Malayan and Australasian realms (Ali and Heaney 2021). The first line in the exact spot of Lydekker’s Line was drawn by Heilprin (1887), ten years before the publication of Lydekker (1896), both interpretations differ by the inclusion (for Heilprin) or not (for Lydekker) of Misool (Ali and Heaney 2021). The subgenus Glossamegilla is, however, restricted under both Heilprin’s and Lydekker’s interpretations of Lydekker’s Line.

The production of maps provides a complement to the work of Lieftinck (1956) who only provided a table of country/islands where the species were distributed. The maps allow more intuitive delineation of the distribution of the *Glossamegilla* in this complex biogeographic region containing the transition area between the Indo-Malayan and Australasian realms (Fig. 40). The maps also permitted to unveiled what seems to be an exchange zone between the Malaysian Peninsula and Sumatra with the cases of *A. himalajensis* (newly recorded from Sumatra), *A. elephas*, *A. insularis*, *A. pagdeni* (also newly recorded from Sumatra), *A. hanitschi*, and *A. pendleburyi* (Figs 26, 27, 29, 30). This exchange zone can probably be explained by the narrow Straits of Malacca that are typically ca 45 km wide, with many islands around Singapore which could facilitate movement of specimens via 5–10 km “jumps”. Despite having relatively scarce occurrences, jumps over large water bodies, such as the English Channel or the straits between Africa mainland and Madagascar, are already known in bees (Cross 2002; Fuller et al. 2005; Dellicour et al. 2014). Moreover, the sea in this region is also shallow with its level being highly variable during the Pleistocene leading to episodes of land merging or separating depending on the sea level, facilitating terrestrial dispersal between islands that are currently separated (Lohman et al. 2011). This information means that the boundary between Malacca and Sumatra is relatively porous over evolutionary time.

Concerning the range of known elevations occupied by the species, five species considered by Lieftinck (1956) have the same range of known elevation while the eight other species have an expanded range of known elevation (Table 8). The species without range change can be explained either by the presence of only the holotype (for *A. proboscidea*), an absence of more recent records since Lieftinck (1956) (for *A. feronia*) or an absence of more recent records with a differing altitude (for *A. himalajensis*, *A. pendleburyi*, and *A. sumatrana*). On the other hand, for the species showing a change in known altitudinal range, sampling bias cannot be distinguished from distribution changes as this region was, and still is, undersampled.

The diversity of *Glossamegilla* was overlooked in Borneo and the Lesser Sunda islands with three new species described, namely *A. celineae* sp. nov. (Borneo: East Kalimantan), *A. floresiana* sp. nov. (Flores), and *A. suzanneae* sp. nov. (Borneo: Sabah and Sarawak). Museum collections of *Glossamegilla* of the Indo-Australian Archipelago probably contain more overlooked species that need to be studied in order to increase the knowledge about the diversity of this subgenus in the region. Moreover, everything discussed concerning the biogeography of *Glossamegilla* requires a modern collecting effort in order to characterise more accurately the current distribution of these species, as the most modern specimen record considered in this article is from 2003, which is more than 20 years ago. Some species even have a majority of specimens collected in 1950 or before, namely *A. celineae* sp. nov., *A. cyrtandrae*, *A. elephas*, *A. feronia*, *A. hanitschi*, *A. jacobi*, *A. proboscidea*, and *A. vigilans* (Figs 26, 28–30).

Based on the results obtained previously, wing morphometrics seems to be an efficient method that can reliably determine the studied species of the subgenus Glossamegilla in Indonesia. However, with an accuracy of ~87.1% for the males and ~94.2% for the females, the males having a higher chance of being misassigned than the females solely based on wing morphometrics, the method is currently not perfect and needs to be used in combination with other morphological or biogeographical features in order to present better results, as suggested for other groups (Dehon et al. 2019; Gérard et al. 2020). For example, in both males and females, it is the pair *A. cyrtandrae*-*A. sumatrana* that cannot be significantly separated from one another using morphometrics, but these two species show strong morphological differences (the metasomal hair bands are clearly visually distinct) and they do not overlap in term of distribution, with *A. sumatrana* restricted to the island of Sumatra and *A. cyrtandrae* restricted to the islands of Java and Bali (Figs 28, 29). The same combination can be used on other overlapping group such as *A. feronia*-*A. pendleburyi* where *A. feronia* have contrasting hair bands on the metasoma and is restricted to Java while *A. pendleburyi* does not have contrasting hair bands on the metasoma and is widely distributed in Sumatra, continental Malaysia, and Borneo (Figs 20, 22, 26). On the other hand, *A. insularis* can be significantly differentiated from *A. cinnyris* (either males or females) based on wing morphometrics, these two species are very close morphologically, the males are almost cryptic and can be mistaken. Moreover, they can be both found in sympatry on the island of Sumatra (Fig. 27; Lieftinck 1956). These encouraging results can lead to a cheaper and less time-consuming approach for broadly supporting the species-level classification of *Glossamegilla* compared to genetic approaches that require more expensive equipment and consumables (Kozmus et al. 2011).

Moreover, the study of centroids size unveiled that the wings of the males are more likely to have similar sizes than for the females (Fig. 2, Tables 2, 3, Suppl. materials 1, 2). This tendency seems to relatively match with body size of the tested species, the males being in general more similar in terms of body size compared to the females (Lieftinck 1944; 1956).

Overall, the number of specimens assessed ranged from 13 to 20 per species and per sex, which means that some groups were below an optimal minimum of 20 but still above the 10 specimens, which is the threshold under which inaccuracies begin to be especially impactful (Cardini et al. 2015). Despite our encouraging results, further collections and collaborations with other museums should be conducted in order to increase the number of specimens studied to limit even more the inaccuracies as well as to expand the number of species studied.

Similarly to *A. cyrtandrae* and *A. sumatrana*, both forms of *A. amymone* are not significantly different based on wing morphometrics (Table 7). We were able to evaluate nine males of the dark form, though this is low for geometric morphometrics (Cardini et al. 2015). The females were not assessed as their number was even lower. Therefore, new collections and work on other museum collections should be done in the future in order to have stronger statistical results on the wing shape of both forms of this species, even if the current tests support the hypothesis of a synonymy. Modern collections of specimens and genetic studies would be ideal to gain a better understanding of this species and its colour variations.

To conclude with the morphology of *A. amymone*, the males appear to have more colour variation compared to females, which is the opposite of that observed in most Anthophorine bees species (e.g., Brooks 1983; Wood and Praz 2024). Indeed, just one female of the pale form has been found (the holotype of *A. bouwmani*, Lieftinck 1944; 1956), while numerous males of the pale form were found with females and males of the dark form.

## ﻿Conclusions

Geometric morphometrics can help to determine the tested species of *Glossamegilla* in the Indo-Australian Archipelago but needs to be coupled with other characters such as the other morphological features or the biogeography. This paper expands the list of known species in the Indo-Australian Archipelago by seven, permitting the elaboration of a new revised and currently complete key. We conclude that the Wallace Line is not a biogeographic border for the subgenus, contrarily to the Lydekker Line, but is a border for the species individually, increasing the knowledge about these “lines of separation” in this archipelago.

## Supplementary Material

XML Treatment for Glossamegilla

XML Treatment for Amegilla (Glossamegilla) floresiana

XML Treatment for Amegilla (Glossamegilla) suzanneae

XML Treatment for Amegilla (Glossamegilla) celineae

XML Treatment for Amegilla (Glossamegilla) vigilans

XML Treatment for Amegilla (Glossamegilla) gigas

XML Treatment for Amegilla (Glossamegilla) anthracina

XML Treatment for Amegilla (? Glossamegilla) tetrataeniata

XML Treatment for Amegilla (Glossamegilla) amymone

## ﻿Additional information

### Use of AI

No use of AI was reported.

### Author ORCIDs

Frédéric Carion https://orcid.org/0009-0001-4944-4353

Maxence Gérard https://orcid.org/0000-0002-2485-0662

Guillaume Ghisbain https://orcid.org/0000-0003-2032-8081

Thomas J. Wood https://orcid.org/0000-0001-5653-224X

### Data availability

All of the data that support the findings of this study are available in the main text or Supplementary Information.

## Supplementary materials

Supplementary material 1 Results of the Tukey multiple comparisons of means (95% family-wise confidence level) test for the differentiation of female’s centroid sizes from the subgenus Glossamegilla in Indonesia Data type docx https://binary.pensoft.net/file/1440747This dataset is made available under the Open Database License (http://opendatacommons.org/licenses/odbl/1.0/). The Open Database License (ODbL) is a license agreement intended to allow users to freely share, modify, and use this Dataset while maintaining this same freedom for others, provided that the original source and author(s) are credited.Frédéric Carion, Maxence Gérard, Guillaume Ghisbain, Thomas J. Wood

Supplementary material 2 Results of the Tukey multiple comparisons of means (95% family-wise confidence level) test for the differentiation of male’s centroid sizes from the subgenus Glossamegilla in Indonesia Data type docx https://binary.pensoft.net/file/1440748This dataset is made available under the Open Database License (http://opendatacommons.org/licenses/odbl/1.0/). The Open Database License (ODbL) is a license agreement intended to allow users to freely share, modify, and use this Dataset while maintaining this same freedom for others, provided that the original source and author(s) are credited.Frédéric Carion, Maxence Gérard, Guillaume Ghisbain, Thomas J. Wood

Supplementary material 3 Results of the pairwise comparisons to determine among female’s wing shape from the subgenus Glossamegilla in Indonesia Data type docx https://binary.pensoft.net/file/1440752This dataset is made available under the Open Database License (http://opendatacommons.org/licenses/odbl/1.0/). The Open Database License (ODbL) is a license agreement intended to allow users to freely share, modify, and use this Dataset while maintaining this same freedom for others, provided that the original source and author(s) are credited.Frédéric Carion, Maxence Gérard, Guillaume Ghisbain, Thomas J. Wood

Supplementary material 4 Confusion matrix of the LDA for the discrimination of female *Glossamegilla* species, including the hit-ratios Data type docx https://binary.pensoft.net/file/1440749This dataset is made available under the Open Database License (http://opendatacommons.org/licenses/odbl/1.0/). The Open Database License (ODbL) is a license agreement intended to allow users to freely share, modify, and use this Dataset while maintaining this same freedom for others, provided that the original source and author(s) are credited.Frédéric Carion, Maxence Gérard, Guillaume Ghisbain, Thomas J. Wood

Supplementary material 5 Results of the pairwise comparisons among male’s wing shape from the subgenus Glossamegilla in Indonesia Data type docx https://binary.pensoft.net/file/1440750This dataset is made available under the Open Database License (http://opendatacommons.org/licenses/odbl/1.0/). The Open Database License (ODbL) is a license agreement intended to allow users to freely share, modify, and use this Dataset while maintaining this same freedom for others, provided that the original source and author(s) are credited.Frédéric Carion, Maxence Gérard, Guillaume Ghisbain, Thomas J. Wood

Supplementary material 6 Confusion matrix of the LDA for the discrimination of female *Glossamegilla* species, including the hit-ratios Data type docx https://binary.pensoft.net/file/1440751This dataset is made available under the Open Database License (http://opendatacommons.org/licenses/odbl/1.0/). The Open Database License (ODbL) is a license agreement intended to allow users to freely share, modify, and use this Dataset while maintaining this same freedom for others, provided that the original source and author(s) are credited.Frédéric Carion , Maxence Gérard , Guillaume Ghisbain , Thomas J. Wood
