# Supplementary material for: ﻿Unravelling Amegilla (Glossamegilla) diversity across the Wallace Line: new species, wing morphometrics, and biogeographic boundaries (Hymenoptera, Apidae)
Source: Zookeys. 2025 Oct 16;1256:1–79. doi: 10.3897/zookeys.1256.162903 (PMC12550509; doi:10.3897/zookeys.1256.162903)
Supplement: Supplementary material 1 — Results of the Tukey multiple comparisons of means (95% family-wise confidence level) test for the differentiation of female’s centroid sizes from the subgenus Glossamegilla in Indonesia [file zookeys-1256-001_article-162903__-s001.docx]

**Supplementary Material 1.** Results of the Tukey multiple comparisons of means (95% family-wise confidence level) test for the differentiation of female’s centroid sizes from the subgenus *Glossamegilla* in Indonesia. Diff is the mean estimate of the difference between the two compared groups. Lwr is the lower limit of a confidence interval. Upr is the upper limit of a confidence interval. * indicates p < 0.05, ** indicates p < 0.01.

| **Species compared** | **Diff** | **Lwr** | **Upr** | **P-value** |
| --- | --- | --- | --- | --- |
| *cyrtandrae-cinnyris* | 0.387 | 0.181 | 0.594 | <0.001** |
| *feronia-cinnyris* | 0.922 | 0.693 | 1.150 | <0.001** |
| *insularis-cinnyris* | 1.709 | 1.503 | 1.916 | <0.001** |
| *pendleburyi-cinnyris* | 1.098 | 0.891 | 1.304 | <0.001** |
| *sumatrana-cinnyris* | 0.721 | 0.504 | 0.939 | <0.001** |
| *feronia-cyrtandrae* | 0.534 | 0.323 | 0.746 | <0.001** |
| *insularis-cyrtandrae* | 1.322 | 1.134 | 1.510 | <0.001** |
| *pendleburyi-cyrtandrae* | 0.710 | 0.522 | 0.898 | <0.001** |
| *sumatrana-cyrtandrae* | 0.334 | 0.135 | 0.533 | <0.001** |
| *insularis-feronia* | 0.788 | 0.576 | 0.999 | <0.001** |
| *pendlevuryi-feronia* | 0.176 | -0.036 | 0.387 | 0.161 |
| *sumatrana-feronia* | -0.200 | -0.422 | 0.021 | 0.100 |
| *pendleburyi-insularis* | -0.612 | -0.799 | -0.425 | <0.001** |
| *sumatrana-insularis* | -0.988 | -1.187 | -0.789 | <0.001** |
| *sumatrana-pendleburyi* | -0.376 | -0.575 | -0.177 | <0.001** |
