# Supplementary material for: ﻿Unravelling Amegilla (Glossamegilla) diversity across the Wallace Line: new species, wing morphometrics, and biogeographic boundaries (Hymenoptera, Apidae)
Source: Zookeys. 2025 Oct 16;1256:1–79. doi: 10.3897/zookeys.1256.162903 (PMC12550509; doi:10.3897/zookeys.1256.162903)
Supplement: Supplementary material 2 — Results of the Tukey multiple comparisons of means (95% family-wise confidence level) test for the differentiation of male’s centroid sizes from the subgenus Glossamegilla in Indonesia [file zookeys-1256-001_article-162903__-s002.docx]

**Supplementary Material 2.** Results of the Tukey multiple comparisons of means (95% family-wise confidence level) test for the differentiation of male’s centroid sizes from the subgenus *Glossamegilla* in Indonesia. Diff is the mean estimate of the difference between the two compared groups. Lwr is the lower limit of a confidence interval. Upr is the upper limit of a confidence interval. * indicates p < 0.05, ** indicates p < 0.01.

| **Species compared** | **Diff** | **Lwr** | **Upr** | **P-value** |
| --- | --- | --- | --- | --- |
| *cyrtandrae-cinnyris* | 0.423 | 0.187 | 0.659 | <0.001** |
| *feronia-cinnyris* | 0.895 | 0.649 | 1.141 | <0.001** |
| *insularis-cinnyris* | 1.185 | 0.946 | 1.424 | <0.001** |
| *pendleburyi-cinnyris* | 1.138 | 0.902 | 1.374 | <0.001** |
| *sumatrana-cinnyris* | 0.681 | 0.446 | 0.917 | <0.001** |
| *feronia-cyrtandrae* | 0.472 | 0.226 | 0.718 | <0.001** |
| *insularis-cyrtandrae* | 0.762 | 0.523 | 1.001 | <0.001** |
| *pendleburyi-cyrtandrae* | 0.714 | 0.479 | 0.950 | <0.001** |
| *sumatrana-cyrtandrae* | 0.258 | 0.022 | 0.494 | 0.023* |
| *insularis-feronia* | 0.290 | 0.041 | 0.539 | 0.013* |
| *pendlevuryi-feronia* | 0.243 | -0.003 | 0.489 | 0.056 |
| *sumatrana-feronia* | -0.214 | -0.460 | 0.032 | 0.127 |
| *pendleburyi-insularis* | -0.048 | -0.287 | 0.191 | 0.992 |
| *sumatrana-insularis* | -0.504 | -0.743 | -0.265 | <0.001** |
| *sumatrana-pendleburyi* | -0.456 | -0.692 | -0.220 | <0.001** |
