# Supplementary material for: ﻿Unravelling Amegilla (Glossamegilla) diversity across the Wallace Line: new species, wing morphometrics, and biogeographic boundaries (Hymenoptera, Apidae)
Source: Zookeys. 2025 Oct 16;1256:1–79. doi: 10.3897/zookeys.1256.162903 (PMC12550509; doi:10.3897/zookeys.1256.162903)
Supplement: Supplementary material 3 — Results of the pairwise comparisons to determine among female’s wing shape from the subgenus Glossamegilla in Indonesia [file zookeys-1256-001_article-162903__-s003.docx]

**Supplementary Material 3.** Results of the pairwise comparisons to determine among female’s wing shape from the subgenus *Glossamegilla* in Indonesia. Results for the females. d is the mean estimate of the difference between the two compared groups. UCL (95%) is the 95^th^ percentile (upper critical value) of the null distribution of Procrustes distances obtained by permutation of group labels, representing the threshold for statistical significance at the 0.05 level. Observed distances “d” greater than this threshold indicate significant shape differences. Z is the difference between the means of the two groups being compared divided by the standard deviation of this difference. * indicates p < 0.05, ** indicates p < 0.01.

| **Species compared** | **d** | **UCL (95%)** | **Z** | **P-value** |
| --- | --- | --- | --- | --- |
| *cinnyris-cyrtandrae* | 0.018 | 0.013 | 2.658 | 0.004** |
| *cinnyris-feronia* | 0.037 | 0.015 | 3.985 | 0.001** |
| *cinnyris-insularis* | 0.026 | 0.013 | 3.199 | 0.001** |
| *cinnyris-pendleburyi* | 0.040 | 0.013 | 3.887 | 0.001** |
| *cinnyris-sumatrana* | 0.016 | 0.014 | 2.016 | 0.025* |
| *cyrtandrae-feronia* | 0.032 | 0.013 | 4.148 | 0.001** |
| *cyrtandrae-insularis* | 0.025 | 0.012 | 3.693 | 0.001** |
| *cyrtandrae-pendleburyi* | 0.034 | 0.012 | 4.519 | 0.001** |
| *cyrtandrae-sumatrana* | 0.010 | 0.012 | 0.929 | 0.181 |
| *feronia-insularis* | 0.034 | 0.013 | 4.060 | 0.001** |
| *feronia-pendleburyi* | 0.014 | 0.013 | 1.715 | 0.034* |
| *feronia-sumatrana* | 0.034 | 0.014 | 3.955 | 0.001** |
| *insularis-pendleburyi* | 0.039 | 0.012 | 4.968 | 0.001** |
| *insularis-sumatrana* | 0.023 | 0.013 | 3.373 | 0.001** |
| *pendleburyi-sumatrana* | 0.036 | 0.013 | 4.313 | 0.001** |
