# Supplementary material for: ﻿Unravelling Amegilla (Glossamegilla) diversity across the Wallace Line: new species, wing morphometrics, and biogeographic boundaries (Hymenoptera, Apidae)
Source: Zookeys. 2025 Oct 16;1256:1–79. doi: 10.3897/zookeys.1256.162903 (PMC12550509; doi:10.3897/zookeys.1256.162903)
Supplement: Supplementary material 6 — Confusion matrix of the LDA for the discrimination of female Glossamegilla species, including the hit-ratios [file zookeys-1256-001_article-162903__-s006.docx]

**Supplementary material 6.** Confusion matrix of the LDA for the discrimination of female *Glossamegilla* species, including the hit-ratios. The species predicted by the LDA are compared to the actual species by the confusion matrix. The percentage of correct classifications are represented by the hit-ratios.

| Predicted species  Actual species | *Amegilla cinnyris* (Lieftinck, 1944) | *Amegilla cyrtandrae* (Lieftinck, 1944) | *Amegilla feronia* (Lieftinck, 1944) | *Amegilla insularis* (Smith, 1857) | *Amegilla pendleburyi* (Cockerell, 1929) | *Amegilla sumatrana* Lieftinck, 1956 | Hit-ratio (%) |
| --- | --- | --- | --- | --- | --- | --- | --- |
| *Amegilla cinnyris* (Lieftinck, 1944) | 16 | 0 | 0 | 0 | 0 | 2 | 80 |
| *Amegilla cyrtandrae* (Lieftinck, 1944) | 0 | 16 | 0 | 0 | 0 | 1 | 80 |
| *Amegilla feronia* (Lieftinck, 1944) | 0 | 0 | 15 | 0 | 2 | 0 | 88.24 |
| *Amegilla insularis* (Smith, 1857) | 0 | 0 | 0 | 19 | 0 | 0 | 100 |
| *Amegilla pendleburyi* (Cockerell, 1929) | 0 | 0 | 2 | 0 | 18 | 0 | 90 |
| *Amegilla sumatrana* Lieftinck, 1956 | 4 | 4 | 0 | 0 | 0 | 17 | 85 |
